# Mapping the evidence supporting FDA-authorized digital therapeutics, before and after authorization

**DOI:** 10.1038/s41746-026-02737-9

**Published:** 2026-05-20

**Authors:** Melissa Amyx, Farzaneh Alebouyeh, Ngan Thi Thuy Phi, Philippe Ravaud, Viet-Thi Tran

**Affiliations:** 1 Université Paris Cité and Université Sorbonne Paris Nord, INSERM, INRAE, Center for Research in Epidemiology and Statistics (CRESS), Paris, France; 2 Centre d’Epidemiologie clinique, AP-HP, Hôpital Hôtel Dieu, Paris, France; 3 Department of Epidemiology, Mailman School of Public Health, Columbia University, New York City, NY USA

**Keywords:** Drug discovery, Health care, Medical research

## Abstract

We aimed to describe the totality of evidence for United States Food & Drug Administration (FDA)-authorized digital therapeutics (DTx). We identified FDA-authorized DTx with a decision from 2019-2022, then identified studies evaluating these DTx. For the initial indication of each DTx, we classified: 1) development/pilot studies; 2) pivotal randomized controlled trials (RCTs); and 3) real-world evidence [RWE]/post-market studies. We separately grouped all studies assessing DTx indication expansion/product modifications. For 45 DTx, we identified 436 studies (median 7, range 0–73 per DTx), spanning over 2 decades (2001–present). The totality of evidence varied from extensive evidence dossiers, with studies from each category for 31.1% DTx, to none for 11.1%. Half (46.7% DTx) had a pivotal RCT. For 13.3% DTx, the lag between study start and/or publication and FDA-authorization was >10 years; half (53.3%) had no available results prior to authorization. The totality of evidence for FDA-authorized DTx varied, reflecting the heterogeneity in DTx characteristics, development, regulation, and evaluation. Given some DTx entered the market with limited or no publicly available data, more transparent, timely dissemination of results and consistent evidence standards, appropriate to the level of risk of the individual DTx, are needed to inform clinical and regulatory decision-making.

## Introduction

In recent years, the availability and use of digital technologies in healthcare has increased dramatically^1–3^. Among these digital healthcare products, digital therapeutics (DTx) use software to both generate and deliver an intervention for preventing, treating, or managing a disease or health condition^4^. Yet, many commercially available DTx lack accessible, rigorous scientific evidence of their efficacy or effectiveness^5–8^ or enter the market without appropriate regulatory authorization, leading to the United States (US) Food & Drug Administration (FDA) issuing Warning Letters^9,10^. Moreover, rigorous evaluation of DTx is vital due to the potential for harm, as found for an online digital training for patients with suicidal thoughts which was found to increase self-harm^11^.

To ensure appropriate regulation and evaluation accounting for the specificities of DTx in comparison to other medical devices without software components (e.g., rapid, continual evolution and dynamic nature)^12^, the FDA has issued multiple guidance documents for digital tools (e.g., software as a medical device^13^, software/mobile applications^14^, artificial intelligence)^15^ and initiated a pilot program for software precertification^16^. Despite these initiatives, most DTx are still regulated through the same traditional regulatory pathways as medical devices without software components, which range from simplified processes for lower risk devices which can claim substantial equivalence to a predicate device and thus may not require clinical evidence (Premarket Notification (510(k))^17^ to more rigorous regulatory reviews requiring valid proof of safety and effectiveness from clinical investigations for higher risk devices (Pre-market approval [PMA])^18^.

Prior research on mapping the evidence supporting the development and evaluation of DTx has taken a bird’s-eye view of these processes. Studies either (1) examined all studies assessing DTx without linking them to individual products^19–22^ or (2) evaluated specific elements of these studies for individual DTx (e.g., availability of effectiveness/efficacy studies and their timing relative to commercial availability or FDA-authorization^7^; highest level evidence available)^23^. Therefore, as in other contexts^24–26^, examining the totality of evidence of DTx, that is integrating and appraising all available evidence and relevant clinical and regulatory factors^24,25^, will provide a better understanding of the current research processes used to develop and evaluate DTx across their full life cycles.

Our objective was to evaluate of the totality of evidence supporting FDA-authorized DTx with a decision in the period 2019–2022 (tracing their predicates and subsequent clearances), focusing on the purpose, characteristics, and timing of studies relative to the first FDA-authorization.

## Results

In this review, we identified FDA-authorized DTx with a decision from 2019-2022. Then, we searched ClinicalTrials.gov, PubMed, DTx websites, and FDA documentation to identify studies evaluating these DTx. Based on the initial FDA-authorization of each DTx, we identified: (1) development/pilot studies; (2) pivotal randomized controlled trials (RCTs); and (3) real-world evidence [RWE]/post-market studies; we separately grouped all studies assessing indication expansion and product modifications. Then, we described the totality of evidence supporting these DTx based on the availability and timing of the studies.

### Identification of DTx and predicate networks from FDA databases

From 12,353 approval and clearance documents retrieved in FDA databases, we identified 45 unique DTx (Figure S1). Most DTx required a prescription (73.3%) and combined hardware/software (71.1%), with the most frequent diseases/conditions targeted being musculoskeletal health (20.0%), diabetes (15.6%), and women’s health (15.6%; Table 1). The earliest FDA-authorization of included DTx was in 2002.

**Table 1:** Characteristics of included DTx at first FDA-authorization (*N* = 45)

DTx characteristics		n (%)
Type of use	Prescription required	33 (73.3)
Age of target population	Children only (0–12 years)	3 (6.7)
	Adults only^a^	21 (46.7)
	Children and adults^b^	7 (15.6)
	Adolescents and adults^c^	2 (4.4)
	Unclear	12 (26.7)
Target disease/s	Smoking cessation	2 (4.4)
	Musculoskeletal health^d^	9 (20.0)
	Women’s health^e^	7 (15.6)
	Respiratory conditions^f^	6 (13.3)
	Diabetes	7 (15.6)
	Arthritis or chronic pain (back, trunk, limbs)	3 (6.7)
	Child health (amblyopia, ADHD)	3 (6.7)
	Essential tremor	1 (2.2)
	Irritable Bowel Syndrome	2 (4.4)
	Sleep disorders (insomnia, nightmare)	2 (4.4)
	Cardiac diseases	2 (4.4)
	Migraine	1 (2.2)
DTx components	DTx with software and hardware components	32 (71.1)
	DTx software which connects/can connect to other hardware devices	5 (11.1)
	Software/mobile application only	8 (17.8)
Year initial FDA-authorization	2002–2004	2 (4.4)
	2005–2009	0 (0.0)
	2010–2014	4 (8.9)
	2015–2018	9 (20.0)
	2019	6 (13.3)
	2020	8 (17.8)
	2021	12 (26.7)
	2022	4 (8.9)

^a^Includes DTx targeting adults (limit not specified; at least 18 and older [no top limit]); where targeting “women”

^b^Includes DTx where lower age bound includes children and adults (from 2-12 years and older)

^c^Includes DTx where lower age bound includes adolescents and adults (from 13 years and older) or is intended for fertility/birth control purposes where age not specified

^d^Muscle weakness, pain, recovery, re-education, training; performance enhancement; injuries prevention and rehabilitation; joint mobility, functional pathologies

^e^Birth control or period and fertility trackers, incontinence or pelvic floor dysfunction (alone or in combination with other disorders)

^f^Prescribed metered dose inhaler (MDI) medication users; patients unable to cough or clear secretions/mobilization of secretions and lung expansion therapy

Attention deficit hyperactivity disorder (ADHD), digital therapeutic (DTx), United States Food & Drug Administration (FDA)

Over half of the DTx had at least one additional FDA-clearance related to a device modification after the initial authorization (*n* = 26, 57.8%; next additional authorization median 1.7, range 0.0-5.0 years following initial approval), for a total of 136 FDA-authorizations (median 2, range 1–11 per DTx; Table S1; full list of FDA-authorizations provided in Table S2).

### Systematic identification of studies evaluating these DTx

Electronic searches identified 3,484 records (Figure S2). After screening and de-duplication, we identified a total of 436 unique studies, representing a median of 7 studies per DTx (range 0–73), spanning over 2 decades (2001-present). No studies were identified for five DTx (11.1%; Table S3). While the majority of these studies were either randomized (*n* = 143, 32.8%) or non-randomized studies (*n* = 269, 61.7%) involving human subjects, a small number were animal (*n* = 8, 1.8%; testing of combined hardware/software devices for heart conditions or spinal cord stimulation) or modeling/simulation/in vitro (*n* = 16, 3.7%) studies.

Most DTx had at least 1 RCT in humans (*n* = 31, 68.9%; median 3, range 1-31), with varied sample size (median 82, range 6-1,500 patients; Table S4).

Among studies involving human subjects (Table 2), uncontrolled prospective studies (38.3%) and parallel RCTs (26.2%) were the most common designs. Physiological/clinical (40.0%), health related quality of life (10.4%), and pain (10.2%) outcomes were most frequently assessed.

**Table 2:** Characteristics of studies involving human subjects^a^ of FDA-authorized DTx, overall and by study classification (*N* = 412)

	Development/Pilot (*n* = 118)	Pivotal RCT (*n* = 21)	RWE/Post-market (*n* = 126)	DTx Indication Modification (*n* = 141)	Unclear (*n* = 6)	TOTAL
	(n, %)	(n, %)	(n, %)	(n, %)	(n, %)	(n, %)
**Study design**						
Randomized trials	45 (38.1)	21 (100.0)	18 (14.3)	58 (41.1)	1 (16.7)	143 (34.7)
Parallel	30 (25.4)	19 (90.5)	12 (9.5)	46 (32.6)	1 (16.7)	108 (26.2)
Cross-over	13 (11.0)	2 (9.5)	6 (4.8)	11 (7.8)	0 (0.0)	32 (7.8)
Factorial	2 (1.7)	0 (0.0)	0 (0.0)	1 (0.7)	0 (0.0)	3 (0.7)
Non-randomized studies	73 (61.9)	0 (0.0)	108 (85.7)	83 (58.9)	5 (83.3)	269 (65.3)
Controlled prospective^b^	11 (9.3)	0 (0.0)	14 (11.1)	6 (4.3)	0 (0.0)	31 (7.5)
Uncontrolled, prospective cohort^c^	49 (41.5)	0 (0.0)	55 (43.7)	52 (36.9)	2 (33.3)	158 (38.3)
Controlled retrospective^d^	0 (0.0)	0 (0.0)	2 (1.6)	1 (0.7)	0 (0.0)	3 (0.7)
Uncontrolled, retrospective^e^	4 (3.4)	0 (0.0)	26 (20.6)	6 (4.3)	0 (0.0)	36 (8.7)
Case-series/case report	5 (4.2)	0 (0.0)	1 (0.8)	8 (5.7)	3 (50.0)	17 (4.1)
Cross-sectional/survey	3 (2.5)	0 (0.0)	1 (0.8)	5 (3.5)	0 (0.0)	9 (2.2)
Mixed-methods/qualitative	1 (0.8)	0 (0.0)	9 (7.1)	5 (3.5)	0 (0.0)	15 (3.6)
**Population type**						
Patients only	117 (99.2)	21 (100.0)	118 (93.7)	135 (95.7)	6 (100.0)	397 (96.4)
Providers only	0 (0.0)	0 (0.0)	2 (1.6)	3 (2.1)	0 (0.0)	5 (1.2)
Mix (Patient + provider/partner/parent)	1 (0.8)	0 (0.0)	6 (4.8)	3 (2.1)	0 (0.0)	10 (2.4)
**Population size (all types)**; median (range)^f^	30 (1, 3268)	188 (59, 558)	201 (2, 144000)^g^	50 (1, 56715)	42.5 (1, 378694)	66 (1, 378694)^g^
**Primary outcome assessed**^h^						
Mortality/survival	1 (0.8)	0 (0.0)	2 (1.6)	1 (0.7)	0 (0.0)	4 (1.0)
Physiological/clinical	55 (46.6)	10 (47.6)	52 (41.3)	45 (31.9)	3 (50.0)	165 (40.0)
Pain	10 (8.5)	4 (19.0)	9 (7.1)	18 (12.8)	1 (16.7)	42 (10.2)
Function (physical/social/role; ADL)^i^	3 (2.5)	1 (4.8)	5 (4.0)	8 (5.7)	0 (0.0)	17 (4.1)
Psychosocial (emotional functioning/wellbeing)	0 (0.0)	0 (0.0)	1 (0.8)	0 (0.0)	0 (0.0)	1 (0.2)
Mental health (cognitive functioning)	4 (3.4)	1 (4.8)	0 (0.0)	9 (6.4)	0 (0.0)	14 (3.4)
HRQL (global quality of life, perceived health status)^j^	9 (7.6)	3 (14.3)	10 (7.9)	19 (13.5)	2 (33.3)	43 (10.4)
Compliance (including withdrawal from treatment)^k^	8 (6.8)	0 (0.0)	13 (10.3)	5 (3.5)	0 (0.0)	26 (6.3)
Satisfaction/delivery of care	5 (4.2)	0 (0.0)	9 (7.1)	11 (7.8)	0 (0.0)	25 (6.1)
Resource use (economic, hospital, operative, medication)	0 (0.0)	0 (0.0)	15 (11.9)	2 (1.4)	0 (0.0)	17 (4.1)
Adverse events^l^	3 (2.5)	0 (0.0)	3 (2.4)	15 (10.6)	0 (0.0)	21 (5.1)
Composite (mix of outcome types)^m^	1 (0.8)	2 (9.5)	2 (1.6)	0 (0.0)	0 (0.0)	5 (1.2)
Enrollment	2 (1.7)	0 (0.0)	0 (0.0)	5 (3.5)	0 (0.0)	7 (1.7)
Device/algorithm development/performance	17 (14.4)	0 (0.0)	5 (4.0)	3 (2.1)	0 (0.0)	25 (6.1)

^a^24 non-human studies were also included; 8 which were animal studies and 16 which were modeling/algorithm/in vitro studies

^b^Controlled prospective: Crossover (non-randomized); Prospective cohort (Multiple arms); Quasi/semi-randomized; Sequential Assignment; pre-test/post-test; before/after

^c^Prospective, no control: Prospective cohort (Single arm); Single Group Assignment; patient registry; case-only; panel study

^d^Controlled retrospective: Retrospective cohort (Multiple arms); case-control; retrospective controlled survey study

^e^Retrospective, no control: Retrospective cohort (Single arm)

^f^Where number of participants reported as “>” a number, took value reported; for studies with multiple participant types (e.g., patients and providers), where one type participant total specified and another type not, took value reported

^g^*n* = 1 RWE/Post-market study where # participants unclear not included in calculation of median (range)

^h^Categorization of first named primary outcome/s based on a published classification system^54^; if a primary outcome was not explicitly named, this was inferred by reviewing the title, abstract, objectives, and results

^i^Function includes: 6-minute walk distance (6MWD), TRG (Tremor Research Group) Essential Tremor Rating Assessment Scale (TETRAS) Subset Score, NYHA classification

^j^HRQL includes: Changes in COPD Assessment Test (CAT) burden category, Computerized Adaptive Test, Mental Health (CAT-MH®): dimensional severity measure of attention-deficit/hyperactivity disorder symptomatology for adults 18 and over, Disturbing Dreams and Nightmare Severity Index, Asthma Control Test (ACT), Subjects Global Assessment of Relief, Urogenital Distress Inventory, insomnia symptom severity (Insomnia Severity Index (ISI))

^k^Includes: engagement, usage/correct usage, retention/completion, adherence

^l^Classified as reported (i.e., hypoglycemia considered adverse event when study of safety, but not if efficacy study)

^m^Includes: IBS Symptom Severity Score, Western Ontario Shoulder Instability Index (WOSI)

digital therapeutic (DTx); United States Food & Drug Administration (FDA); randomized controlled trial (RCT); real world evidence (RWE); irritable bowel syndrome (IBS); health-related quality of life (HRQL); activities of daily living (ADL)

### Classification of studies by their purpose, characteristics, and timing

We classified studies as development/pilot (*n* = 132, 30.3%), pivotal RCT (*n* = 21, 4.8%), RWE/post-market (*n* = 127, 29.1%), or DTx indication expansion/modification (*n* = 145, 33.3%). Though the majority of studies could be classified, a small number were not (*n* = 11; Table S3). For classified studies involving human subjects, the patient population size varied between study types (development/pilot: median 30, range 1-3,268; RWE/post-market: median 199.5, range 2-144,000; DTx indication expansion/modification: median 50, range 1-56,715; pivotal RCT: median 188, range 59-558 [Table S5]).

Examining identified RCTs, half of the DTx (*n* = 22, 48.9%) had at least one RCT in the development/pilot category (median 1, range 1-12). Similarly, roughly half of the DTx (*n* = 21, 46.7%) had a pivotal RCT (one each based on category definition). About one quarter of DTx (*n* = 11, 24.4%) had at least one RCT in the RWE/post-market category (median 1, range 1-4; Table S4).

**Fig. 1:**
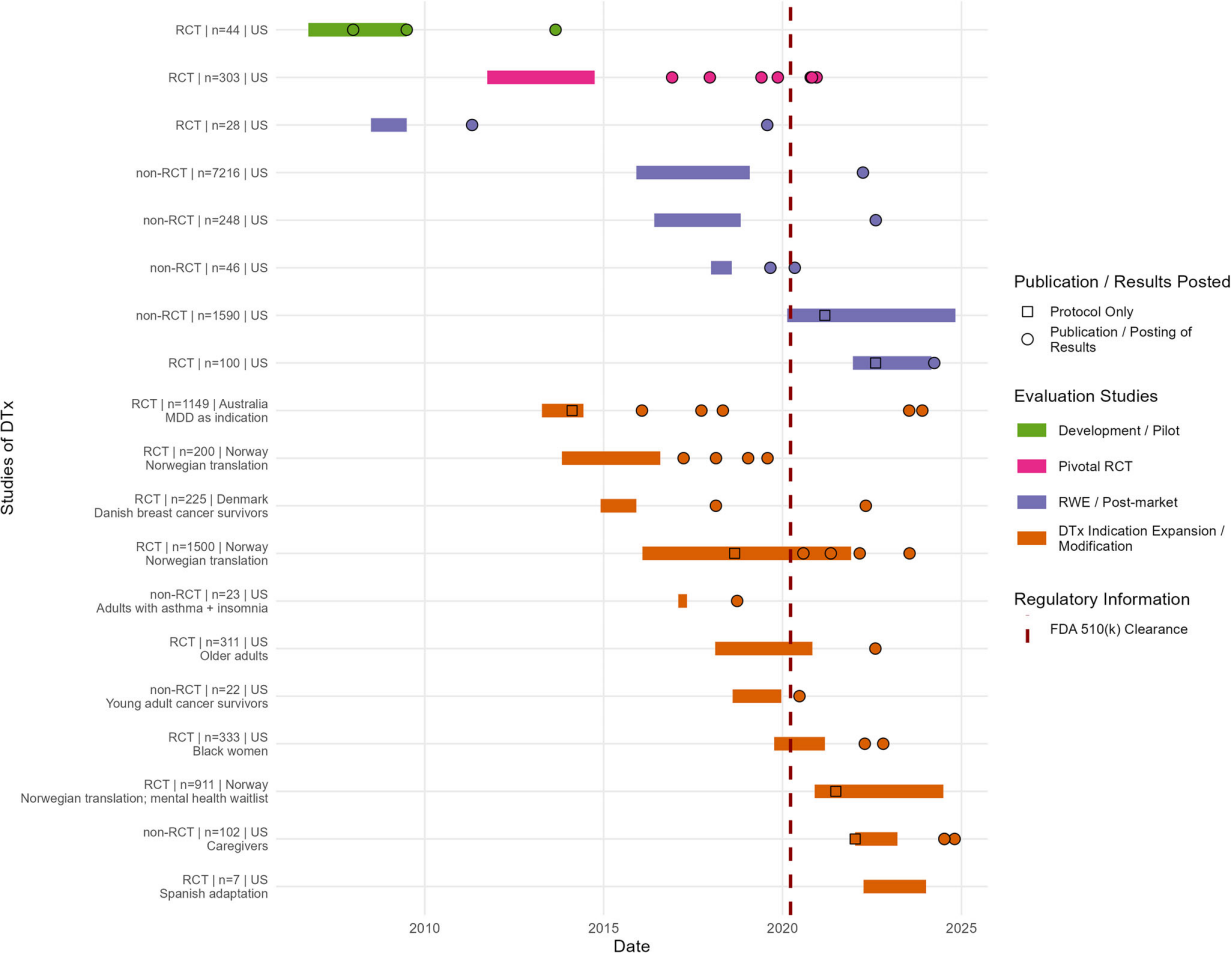
Totality of evidence for the DTx Somryst, including evaluation studies and FDA-authorization. Somryst, a DTx using computerized behavioral therapy based on an established face-to-face intervention to treat chronic insomnia and marketed by Pear Therapeutics, was cleared by 510(k) Premarket Notification on March 23, 2020 based on substantial equivalence to a different DTx (reSET) for substance use disorder from the same manufacturer. The timeline demonstrates the extensive evidence dossier for Somryst, including studies within each category. Spanning a period of almost 20 years, the earliest available evidence came from a small development/pilot study assessing the DTx’s feasibility and efficacy (RCT; *n* = 44; 13.5 years prior to first authorization), followed shortly after by the pivotal RCT (*n* = 303; 8.5 years prior to first authorization). RWE/post-market studies were dated both before and after FDA clearance (*n* = 6 studies [2 RCTs] in US with study start over 11 years ; range 28–7,216 participants). Of the 2 RWE/post-market studies with the latest start dates, the larger reflects a more traditional non-RCT RWE/Phase 4-type study of the efficacy in and engagement of real-world users, while the smaller was an RCT related to an implementation/healthcare integration study. The RWE/post-market studies preceding FDA clearance reflect the commercial availability prior to FDA clearance, with the two smaller studies being pilots conducted in specific populations (non-RCT in Appalachian women, RCT in cancer survivors) covered in the FDA clearance, the largest reflecting a more traditional non-RCT RWE/Phase 4-type pragmatic effectiveness study, and the final medium-sized non-RCT focusing on healthcare utilization. Similarly, both before and after the initial FDA clearance, DTx indication expansion/modification studies were identified (*n* = 11 studies in different populations conducted over a decade; range 7–1,500 participants), including both larger RCTs for these new populations/indications/versions not covered in the initial FDA clearance and non-RCTs of versions tailored for specific populations. digital therapeutic (DTx); United States Food & Drug Administration (FDA); randomized controlled trial (RCT); real world evidence (RWE); United States (US).

To illustrate the assessment of the totality of evidence for a given DTx, we provide a detailed example focusing on Somryst, a DTx using computerized behavioral therapy to treat chronic insomnia (Fig. 1). This DTx had an extensive evidence dossier including studies within each category spanning a period of almost 20 years. The earliest available evidence came from a small development/pilot study assessing its feasibility and efficacy to improve sleep (RCT; *n* = 44; 13.5 years prior to first FDA-authorization), followed shortly after by the pivotal RCT (*n* = 303; 8.5 years prior to first authorization). The RWE/post-market (*n* = 6 studies in US over 11 years; range 28-7,216 participants) category included larger non-RCT pragmatic effectiveness studies, as well as smaller pilot studies examining effectiveness in specific populations (e.g., non-RCT in Appalachian women; RCT in cancer survivors) covered in the FDA-clearance. DTx indication expansion/modification (*n* = 11 studies over 16 years; range 7-1,500 participants) studies were heterogeneous in timing and characteristics, including RCTs (*n* = 8) for new populations/indications/versions not covered in the initial FDA clearance (e.g., language translations and/or non-US populations, older adults) and non-RCTs (*n* = 3) of versions tailored for specific populations (caregivers, young adult cancer survivors, adults with asthma and insomnia).

**Fig. 2:**
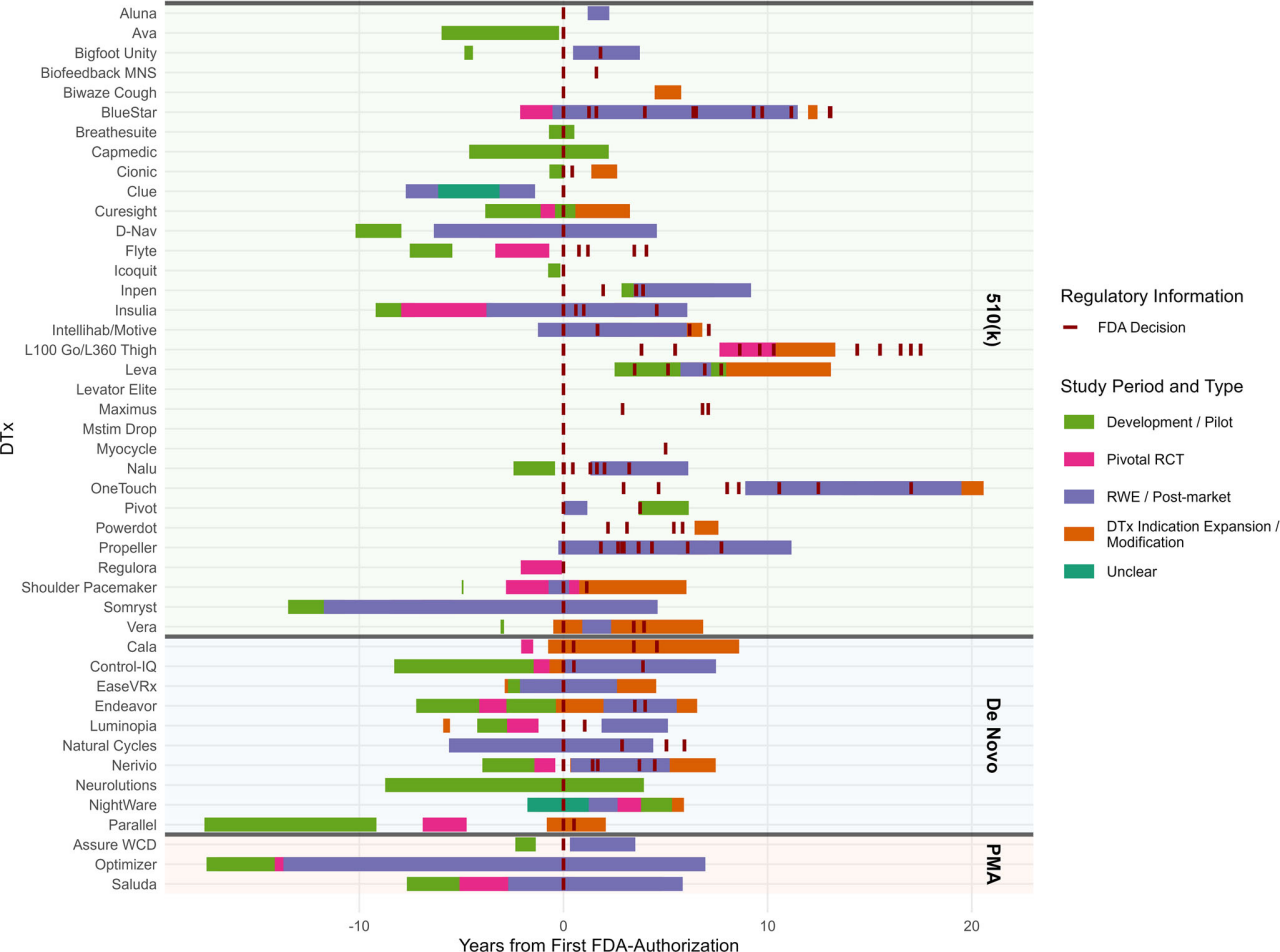
Summary of the totality of evidence for each DTx, by study classification (*N* = 45 DTx evaluated in *N* = 436 unique studies). The totality of evidence supporting FDA-authorized DTx is summarized based on study periods, in relation to first FDA-authorization (set as time 0), of a study evaluation classification framework. This classification has four categories, briefly: *Development/pilot*: in initial target population/indication per first FDA-authorization focusing on DTx development and/or pilot studies in preparation for the pivotal RCT. *Pivotal RCT*: in initial target population/indication per first FDA-authorization. *RWE/post-market*: in initial target population/indication per first FDA-authorization and using real-world data, focusing on evaluation of DTx in practice, and/or following commercial availability. *DTx indication expansion/modification*: outside initial target population/indication and/or a modified version of the DTx per first FDA-authorization. Study periods reflect the earliest study start date and latest study completion date for studies of DTx within each category (e.g., development/pilot study period starting with the earliest development/pilot start and ending with the latest completion). For simplification, where studies periods overlap, the subsequent period is interposed on the earlier period and study publication/results availability are not illustrated, resulting in some masking of data. For complete information regarding all studies/milestones for each DTx, see Document S1. Based on this figure, some DTx are supported by comprehensive bodies of evidence (each category represented within the totality of evidence; *n* = 14; 31.1%). However, other DTx lack studies of any type (*n* = 5; 11.1%) and, additionally, over half lack studies within at least one category (*n* = 26; 57.8%), though in some cases this reflects DTx with both development/pilot and pivotal studies prior to first authorization but without further RWE/post-market and/or DTx indication expansion/modification studies (*n* = 5; 11.1%). Length of time from first study start to first authorization varied ( > 10 years for *n* = 4; 8.9%; study start only following authorization for *n* = 8; 17.8%). digital therapeutic (DTx); United States Food & Drug Administration (FDA); randomized controlled trial (RCT); real world evidence (RWE).

The totality of evidence highlighted strong heterogeneity across DTx (Document S1; Fig. 2). For some, extensive evidence dossiers were compiled, with at least 1 study identified within each category (*n* = 14; 31.1%); other DTx had both development/pilot and pivotal studies prior to first authorization but without further RWE/post-market and/or DTx indication expansion/modification studies (*n* = 5, 11.1%). In contrast, some DTx lacked studies of any type (*n* = 5; 11.1%). Further, while some DTx had long periods of development/evaluation (first study start and/or publication/results available >10 years before first authorization: *n* = 6; 13.3%), for others, no evidence was available to demonstrate studies were conducted (*n* = 13; 28.9%) or had results published (*n* = 24; 53.3%) before first authorization.

Considering the timing by classification (Table S6), development/pilot study and pivotal RCT periods tended to start prior to first FDA-authorization (Figure S3). Timing of the start of RWE/post-market studies was heterogeneous, with some of these studies starting prior to authorization and others after due to simultaneous evaluation in multiple countries and commercial availability of the DTx prior to FDA-authorization; virtually all ended after first FDA-authorization (Figure S4). Trends for first publication/posting of results for each study category were similar for development/pilot and RWE/post-market studies, though for pivotal RCTs this was balanced and few DTx had pivotal RCT results available at time of first authorization (*n* = 9; 20.0%; Figure S5).

In the stratified analyses, more DTx first authorized through the 510(k) pathway (65.6% versus 23.1% PMA or De Novo pathway; Table S7; Figures S6-8) and with combined hardware/software (65.6% versus 23.1% software/mobile application [app]-only or with connection to hardware; Table S8; Figures S9-11) did not have a pivotal RCT. Though no differences were noted in pivotal RCT availability by year first FDA-authorization (no pivotal RCT: 2020-2022: 54.2%; 2002-2019: 52.4%), as would be expected, DTx with earlier first authorization were more likely to have initiated RWE/post-market studies than those (71.4%) with later authorizations (45.8%; Table S9; Figures S12-14). DTx authorized through the PMA or De Novo pathways (versus 510(k) premarket notification) or with more recent first authorization (2020-2022 versus 2002-2019) had studies which were started, completed, and had results published/available slightly earlier relative to their first authorization, for all study types related to first authorization (development/pilot, pivotal RCT, RWE/post-market). Similar trends were seen for software/app-only or with connection to hardware DTx (versus combined hardware/software DTx).

## Discussion

We identified 45 FDA-authorized DTx with a decision from 2019-2022 and 436 unique studies spanning over 2 decades (2001-present) supporting these DTx. By grouping these studies based on their purpose, characteristics, and timing, we provide a coherent portrayal of the current evaluation and regulatory processes of DTx. Reflecting the considerable variation in their characteristics, development, regulation, and evaluation, the totality of evidence supporting the DTx was heterogeneous: for some extensive evidence dossiers from their full life cycle were identified, while little to no evidence was found for others. Variations in the timing from study start/completion/publication to FDA-authorization were also found, as notably over 10% had long evaluation periods before first FDA-authorization ( > 10 years) while for over half, no publication of results was available prior to authorization.

Previous reviews have also found that evidence supporting some FDA-authorized DTx is lacking. Specifically, one found that fewer than half of DTx had results from an effectiveness or efficacy study available at approval/clearance^6^, while another found that very few (10%) had been evaluated in a rigorous, randomized, blinded study^23^. Yet, whether DTx lacking evidence were not evaluated or if their evaluations were unpublished is unknown, with the proportion of unpublished data potentially high, even for data provided in support of DTx authorizations (20–51% studies mentioned in FDA documentation unpublished, depending on the context)^27–30^. The lack of evidence for some DTx can also be attributed to their development by small tech companies which, unlike traditional pharmaceutical and medical device manufacturers, may lack the knowledge or funding to conduct clinical research. Further, the bankruptcy Pear Therapeutics^31^, a pioneer in digital health, demonstrates the financial difficulties of DTx development, marketing, and implementation into clinical practice and could deter DTx developers from investing in a complex evaluation of their devices if not strictly required.

Beyond FDA requirements for authorization, post-market evaluations are critical, but were only found for 26 of our DTx. Continual monitoring following initial DTx authorization is imperative, as some device recalls were identified for several included DTx in the course of the project, including one with Class 1 recalls. Additionally, the potential global reach of DTx complicates evaluation and regulation due to simultaneous development/commercialization in multiple countries which may have different evidence requirements (e.g., evidence requirements are less stringent and transparent in the European Union compared to the US)^27,32^ and diverse priorities and trends in terms of DTx characteristics, evaluation, regulatory patterns, and market development^30,33,34^. Thus, in addition to FDA efforts, international groups have been working to harmonize standards for software-based devices^35,36^.

Regarding the timing of DTx evaluations, the range of zero to almost 18 years showed the wide variability in the delay between initial development/pilot studies and first FDA-authorization. The greatest delay for our DTx was thus longer compared to estimates for medical devices in general (Exempt, 510(k), De Novo, PMA: 30 days to 7 years)^37^, though many of the PMA and De Novo pathway-approved DTx had shorter development periods than novel therapeutic complex medical devices (PMA: 13 years)^38^. Concerning post-market evidence, though we found less evidence for more recently-authorized DTx which inherently have less follow-up time (1-2 years), additional post-authorization evidence will potentially become available in the years to come (55% post-market studies of high-risk PMA-approved medical devices completed within 8-10 years^39^; 38% at median 4-year follow-up for PMA-approved cardiovascular devices)^40^. Additionally, PMA and De Novo-approved DTx had more RWE/post-market evidence which was conducted sooner following authorization compared to 510(k)-authorized DTx, including FDA-required post-authorization studies for 3 DTx (1 De Novo; 2 PMA).

An additional complexity when considering the timing of evidence generation for DTx is the wide time period over which the included DTx were first authorized (2002-2022). For several DTx, the extended time period of their predicate networks reflected continual incremental changes to the DTx, which made it challenging to pinpoint the exact time/authorization that the device became a DTx. Additionally, the regulatory changes which occurred during this period could impact the evidence requirements. Though we included a subgroup analysis to address this issue, additional research would be required to more clearly examine the impact of regulation changes on evidence generation. Thus, a key challenge is to ensure efficient, thorough, and appropriate evaluation of DTx, both before and after authorization, particularly considering the short life cycles of medical devices^41,42^ and even more so for software-based medical devices^43,44^.

Our study had a number of strengths. Foremost, our extensive search of multiple sources, including non-traditional sources such as DTx websites, and iterative search strategy identified a broad range of literature conducted over a period of more than 25 years, including studies under different DTx or manufacturer names. Given previous reviews did not evaluate the totality of evidence and full regulatory progression across the full DTx life cycles, our study adds richness through a more comprehensive, robust assessment of the development, evaluation, and regulation of DTx, particularly related to the purpose, timing, and characteristics of the studies of DTx. Specifically, in addition to pre-authorization development studies and pivotal RCTs, we include evidence from RWE/post-market and DTx indication expansion/modification studies, which to our knowledge had not previously been thoroughly evaluated, providing important information related to evidence generation after approval relevant to clinical care in practice.

Some limitations should be noted. We included only a portion of DTx currently commercially available, which may not be representative as our search of FDA databases covered only a four-year period and the seeking of FDA-authorization could encourage more formal, stringent development/evaluation than for non-FDA- authorized DTx. Similarly, despite our extensive search, evidence could have been missed, whether because it is not publicly available or due to search challenges (e.g., several included DTx were sold/acquired or had at least one name change). Misclassification of study categories was possible. Specifically, the vast heterogeneity of the DTx included, with potentially simultaneous development, evaluation, regulation, and commercialization in multiple populations/countries and for different indications, complicated categorizations. Additionally, the information necessary to make these determinations relies on that reported by manufacturers and researchers and these details were inconsistently reported. Finally, missing data was extensive for study dates, with study start and completion dates frequently lacking for publications, and all timepoints of interest (e.g., true “start” of development and first commercial availably of DTx, all trial registrations and authorizations in other countries, discontinuation of DTx) could not be identified.

In conclusion, the totality of evidence supporting FDA-authorized DTx was heterogeneous in quantity and timing, reflecting the considerable differences between DTx characteristics, development, regulation, and evaluation. While some differences in evidence availability can be expected due to the differences in risk of the devices, more transparent, timely dissemination and consistent evidence standards, appropriate to the level of risk of the individual DTx, are needed to inform clinical and regulatory decision-making. The ongoing efforts of regulators, including the FDA, to update regulatory frameworks for devices which incorporate software represent essential progress to provide innovative, nimbler processes for these devices. A key challenge moving forward is to assure the regulations balance the need for appropriate, transparent evidence throughout the DTx lifecycle without creating unnecessary burdens for lower risk DTx. As many DTx manufacturers are outside the traditional pharmaceutical and medical device industries, early engagement with both regulators and researchers to guide development and evaluation, before and after authorization, is vital. Future research should address additional aspects of DTx evaluation, such as study quality and outcomes evaluated, particularly for evaluations conducted after FDA-authorization.

## Methods

We conducted this analysis of the totality of evidence for DTx in three parts. First, we identified FDA-authorized DTx with a decision from 2019-2022, tracing DTx predicates and identifying subsequent clearances for identified DTx within FDA databases. Then, we performed a comprehensive review of publicly available information from multiple sources to locate studies evaluating these DTx. Finally, we classified these studies by their purpose, characteristics, and timing in order to assess the totality of evidence for the included DTx.

### Ethics statement

Ethical approval for the study was not required, as this study exclusively analyzed publicly available information.

### Eligibility criteria for DTx

We included devices authorized (approved or cleared) by the FDA with a decision from January 2019 to December 2022 which met the International Organization for Standardization definition of a DTx (“intended to treat or alleviate a disease, disorder, condition, or injury by generating and delivering a medical intervention”)^35^, as interpreted by the DTx Alliance^4^. These DTx could function independently or in conjunction with ancillary components (hardware devices, tandem clinician interventions) and be intended for prevention, treatment, or management of diseases and disorders.

We excluded products which did not contain software, did not deliver an intervention (e.g., for diagnosis or monitoring only), or were solely intended for clinical or clinician use (e.g., tools used by clinicians to generate a treatment plan or which generate a treatment which is then delivered by a clinician).

### Data sources and search strategy for DTx

FDA databases (Premarket Notification (510(k))^17^, Premarket Approval [PMA]^18^, De Novo^45^, Humanitarian Device Exemption [HDE]^46^) were searched in order to identify devices with a decision from January 1, 2019 to December 31, 2022.

### Selection process for DTx

For the devices identified in these searches, one reviewer first screened devices by name. Next, devices intended for clinical use or diagnosis only or which did not include software were excluded. Finally, one reviewer reviewed the information included in the product summary of FDA-authorization records to determine device eligibility. Duplicate devices were identified and removed by cross-checking names, manufacturers, and relevant characteristics.

Selection involved 2 reviewers (NTTP, MA) working independently under the supervision of a senior reviewer (VTT) to resolve disagreements, as described previously^6,22^. In cases where eligibility could not be determined solely based on FDA documentation, device websites and app stores were reviewed for pertinent information.

### Predicate network and subsequent clearance review

First, one researcher (FA), under the supervision and with verification by a supervisor (MA), traced the predicate networks of included DTx (searches conducted August 1–13, 2024) identified from the 510(k) database, using similar methodology, as previously described^47^. Specifically, starting with the 510(k) clearances, predicate devices named by manufacturers in FDA documentation (statement, decision, decision summary, summary) were traced back until the first FDA-authorization (De Novo request or PMA approval) or non-DTx related device (predicate different from DTx, based on name and/or manufacturer, or with missing/incomplete public documentation) was reached.

Then, to identify subsequent clearances related to modifications to all included DTx (initially identified within 510(k), PMA, De Novo, or HDE databases), one researcher (FA), under the supervision and with verification by a supervisor (MA), searched the 510(k) database^17^ using the DTx name (in Device Name) and manufacturer (in Applicant Name). Searches were conducted separately, using names as reported in the initially identified documentation.

During the predicate review, two sets of two DTx each (Motive Knee Wrap and Intellihab; L100 Go System and L360 Thigh System) had common authorization networks. In the first set, one DTx (IntelliHab system, intended for prescription use) was the named predicate of the second (Motive Knee Wrap, intended for over-the-counter use). In the second set (L100 Go System and L360 Thigh System), both DTx authorizations reference a common predicate device (L300 Go System) and were subsets of that device (L100 Go System is a lower leg cuff only; L360 Thigh System is the Thigh Standalone configuration). Therefore, these were considered as “duplicates” and were combined in the analysis to reflect this common authorization process.

### Data extraction for DTx characteristics

To extract DTx characteristics, we used a standardized form to extract information from the earliest identified authorization, as information could change in subsequent authorizations. Characteristics extracted included general information (authorization number, regulation pathway, decision date), whether a prescription was required, target age and disease/condition, and components (combined hardware/software; software with connection to other hardware possible/necessary; software/app only). The target diseases/conditions for each DTx were mapped to the analyzed DTx by a single reviewer (MA) based on the extraction data, in coordination with the senior reviewer (VTT). For included DTx, 1 reviewer (MA) extracted the relevant DTx characteristics.

In the predicate network and subsequent clearances review, for all DTx FDA-authorizations identified, the authorization number, decision date, and references to clinical studies were extracted. This review was completed by two researchers, with one researcher (FA) extracting information, under the supervision and with verification of a second reviewer (MA).

### Eligibility criteria for studies evaluating included DTx

All primary studies evaluating included DTx (interventions with the same name or which could be clearly be linked to DTx based on information available from studies/website/etc.) were eligible. Studies were eligible regardless of whether or not they were conducted by developers/researchers as part of the formal development process (i.e., Phase I–IV). We included trial registrations, FDA-required post-authorization studies (Post-approval studies [PAS]^48^ and Postmarket Surveillance studies)^49^, and scientific publications.

We excluded studies in which the DTx was not the intervention evaluated (DTx served as control; not evaluated independently or similar DTx/non-DTx combined as intervention). Additionally, studies were excluded where only one component of the DTx was evaluated (e.g., evaluates validity of continuous glucose monitor [CGM] or carbon monoxide [CO] measurements) or which utilized the DTx for non-therapeutic purposes (e.g., monitoring only [CO monitors]). As our interest was in identifying primary studies, reviews and meta-analyses and studies expounding hypotheses were excluded. We excluded the following publication types: editorials, consensus statements, guidelines, comments/commentaries, replies; conference abstracts, presentations, proceedings, reports; government reports; books; and white papers and non-journal articles. Finally, we excluded trials/studies with incomplete or insufficient information to make an eligibility decision (articles with no full-text available; withdrawn or retracted CT.gov records; award abstracts; studies referenced in FDA or website text, but not formally reported sufficiently to extract necessary data/verify underlying study).

### Data sources and search strategy (initial searches) for studies evaluating included DTx

To identify publicly available information (registered clinical trials and scientific literature) regarding evaluation for each DTx (including “duplicate” DTx to ensure relevant records were located), we searched four sources: CT.gov^50^, MEDLINE via PubMed^51^, DTx websites, and FDA documentation (510(k)^17^, PMA^18^, De Novo^45^, HDE^46^) identified in DTx searches.

To identify trials records of DTx registered on CT.gov, one researcher (MA; July 4, 2024) carried out four separate searches (using “exact terms” when the website suggested alternate results; no filters) using the DTx name as “other terms” and Intervention/Treatment and then using the manufacturer name as Sponsor/Collaborator and Sponsor (Lead). After each search, results were saved to the “My Saved Studies” list to remove duplicates, and then after all searches were conducted, this list was exported for screening.

To identify published studies of DTx, one researcher (MA; July 1, 2024) searched MEDLINE via PubMed using the prompt: (“DTx Name”) OR (“Manufacturer name”). Studies identified were then exported for screening. For both the CT.gov and PubMed searches, if this initial search strategy returned large number of results ( > 100) suggesting the search was not sufficiently specific, one reviewer (MA) refined the search by adding additional keywords or by removing names which generated a large number of extraneous results.

To identify additional studies not captured in these searches, including trials registered on other platforms (e.g., Australian New Zealand Clinical Trials Registry [ANZCTR]^52^, International Standard Randomized Controlled Trial Number [ISRCTN] registry^53^; FDA PAS^48^ and Postmarket Surveillance^49^ studies) and publications not indexed in PubMed, FDA documentation and the DTx websites were reviewed. One researcher (MA; June 28–July 4, 2024) reviewed the full FDA-authorization documents (for the primary DTx authorization used for DTx eligibility) to identify any referenced studies or trials.

Finally, one reviewer (MA; July 16–25, 2024) reviewed the websites of the included DTx to identify additional studies and trials. The most relevant website(s) for the DTx was identified through a Google search, first using the DTx name. If a current website not clearly identified, the search was repeated using manufacturer name. The identified websites were then reviewed, focusing on pages most likely to include relevant information (i.e., pages dedicated to: clinical trials, evidence, research; information for clinicians; main landing page; user manuals and product brochure). Pages considered unlikely to contain relevant information were not systematically reviewed (i.e., pages dedicated to: videos, blogs, news and events, about us; funding/insurance; privacy/legal information). Additionally, information requiring registration or contact with the company was not sought. When the first reviewer (MA) did not locate a website or studies/trials for a DTx, a second reviewer (FA; July 30, 2024) verified lack of studies referenced. All potentially relevant studies and trials were extracted (MA and FA) for screening.

After completing these four searches, identified trials and studies were compiled and de-duplicated to identify unique CT.gov, post-authorization, and other registration records and PubMed and non-PubMed articles or preprints (MA) for screening.

### Selection process (initial searches) for studies evaluating included DTx

One reviewer (MA), under the supervision of the senior reviewer (VTT), screened identified records to determine eligibility. For trial registry records (CT.gov, post-authorization, other registrations), a single round of screening was conducted to determine eligibility based on the current record. For articles (PubMed, other publications), titles and abstracts were first screened to select articles for full text review. Next, the full texts of these potentially relevant articles were reviewed to determine eligibility. During screening, reviews focusing on a single included DTx were tagged in order to review their reference lists for additional studies.

### Iterative searches/reference list review for studies evaluating included DTx

Following the initial selection, iterative reviews of references lists and linked studies of included studies were conducted. For trial registrations and FDA-mandated post-authorization studies, one reviewer (FA or MA) extracted all linked publications. For articles, one reviewer (FA) performed a review of reference lists of certain included articles (efficacy and effectiveness studies included in a previous study^6^) to extract additional publications and trial registration numbers. One reviewer (MA) identified primary DTx studies included in the reviews of DTx identified in the first round. These records were de-duplicated and screened for inclusion (MA), as described for the first round.

For the additional records identified in this second round, one reviewer (FA) continued reviewing all new trial registration records to identify linked studies and all publications to identify additional trial registrations, until no new records were identified. The remaining article references lists were not systematically reviewed, but additional studies identified during data extraction or study matching (described below) were included, through the end of extraction (June 20, 2025), at which point minimal new studies were being identified. These records were de-duplicated and screened for inclusion (MA), as described for the first round.

Following completion of all searches and closing of the study database for new entries, for studies selected for inclusion, one reviewer (MA) de-duplicated the results to identify unique evaluations of each DTx (i.e., matching records related to same underlying study). Where possible, studies were matched by record identifier (PMIDs, NCT numbers, other trial identifier) or direct linkage across sources (e.g., NCT records linked in FDA database). Additionally, studies were matched by their characteristics (e.g., acronyms, authors, recruitment dates, sample sizes, registration/IRB numbers). In practice, we kept linkages as reported, unless they were clearly erroneous (based on differing study designs, locations), such that if separate CT.gov records were recorded for related studies (i.e., separate records for related studies conducted in two locations) they were considered different studies, or conversely if reported in a single CT.gov record (one record for pilot and pivotal phases), they were considered the same study. For publications based on RWE/app data (e.g., OneTouch), we considered records as the same study where explicitly described by authors as such or where the study populations clearly overlapped.

To describe individual unique studies where multiple records for a single study were located (ex: both CT.gov record and publication/s), we prioritized data to use in analyses to describe studies (study design/purpose, population, primary outcome; study dates) in the following way: trial record (due to consistency of reporting), initial trial record where an extension or continued access protocol was later filed, and publication with most complete report where a trial record was not located.

### Data extraction for studies evaluating included DTx

We developed a standardized data extraction form. For all included study records, one researcher (FA), under supervision (MA, VTT), extracted relevant data (August 20, 2024–June 20, 2025). Data extracted for all records was identifying information (record source [CT.gov, other registry, PubMed, other] and type [trial record or publication]; record identifier [registry number, PMID, other]) and study purpose/objective, design, population, and outcomes, as detailed below.

Study type was extracted as human or non-human (animal study; algorithm/modeling, including cost-effectiveness). For human studies, design was extracted and categorized as randomized (parallel [including waitlist control]; cross-over; factorial) or non-randomized (controlled prospective [non-randomized crossover; multiple arm prospective cohort; quasi/semi-randomized; sequential assignment]; uncontrolled prospective [single arm prospective trial]; controlled retrospective [multiple arm prospective cohort; case-control]; uncontrolled retrospective [single arm prospective trial]; case series/report; cross-sectional/survey; mixed methods/qualitative). Where studies included arms/substudies with different designs, we assigned the study as the most rigorous design reported (i.e., randomized over non-randomized; prospective over retrospective).

Additionally, we extracted the number and type (patient, other stakeholder [provider, parents, partner]) of participants. If the exact/actual total was not clearly reported, we took the estimated total (CT.gov records), lowest possible total (where reported as “>” a given number), or the known total (where multiple groups were included, but totals for all not clear). Study location, based on where participants were recruited (country, or continent if country not reported) was also extracted

We extracted the named primary outcome/s. Where a primary outcome was not explicitly named, the Title and Study Overview (for trial records), title, abstract, objectives, and results (for publications) were reviewed to infer the primary outcome/s. Then, the primary outcome was categorized based on a published categorization system^54^, analyzing only the first reported primary outcome where multiple were reported.

Finally, study dates (study start and completion dates; publication or availability of results on trial registry) were extracted. For trial registrations, we extracted study start and completion dates and dates results were posted to the registry. For articles, we extracted reported study dates (start and/or completion) and the earliest publication date (either the official date of publication or date of the online-only version posted, whichever occurred first). Additionally, we noted articles which were a protocol only (i.e., results not reported). Where the exact date not provided, we set the date as 1 (for day and month) to allow for calculations; if a date range was reported, we took earliest for study start and latest for study completion.

### Classification of studies by their purpose, characteristics and timing

Then, based on a full consideration of the DTx evaluation and regulation ecosystem (all identified FDA-authorizations and studies), we classified included studies based on their purpose, as informed by their stated objectives, population characteristics (same as initial FDA-authorization or not; human or non-human), sample size, length of study/follow-up, design, and timing in relation to first FDA-authorization of the DTx, commercial availability, and other studies. First, we defined, for each DTx, its initial indication, that is the first indication for which it was authorized. Within this indication, we categorized studies as development/pilot, pivotal, or RWE/post-market studies. Studies related to other indications or versions of the DTx, regardless of their other characteristics, were categorized as DTx indication expansion/modification studies. The categorizations were performed by a single reviewer (MA), in coordination with the senior reviewer (VTT). The categories utilized and the relevant study characteristics used (in order of importance) to make this determination were:

Development/pilot*:* studies in the *initial* target population/indication per first (earliest) identified FDA-authorization of the DTx related to development, proof-of-concept, or preparation for a pivotal study and/or were referred to as development/pilot in later studies, as detailed below:

- Study types/purposes:
  - Non-human/preclinical: animal studies; initial algorithm development, in silico and simulation studies, in vitro
  - Human: any study design with the following purposes:
    - Development of DTx: evaluations of
      - Reliability/validity/accuracy/precision/correlation
      - Acceptability and user perspectives/perceptions/satisfaction, usability
      - Non-DTx or prototype/early version of device
      - Related to identification of appropriate target population/s and indication/s
    - Proof-of-concept of DTx: reported as preliminary, first/initial/early evidence/step/experience
    - Pilot studies in preparation for pivotal study
      - Objectives related to piloting and/or feasibility for future pivotal study
      - Explicitly reported in FDA documentation, other DTx study records, or on DTx website as a pilot/feasibility study leading to the pivotal study
- Characteristics relative to other studies of same DTx or clinical trial norms
  - Smaller sample size and/or scope (in terms of setting: conducted in hospital or supervised setting [ex: diabetes DTx]) or clinical trial norms (in practice, <40 participants)
  - Shorter length of follow-up, relative to other RCT/s of same DTx or clinical trial norms (in practice, <1-week follow-up)
- Timing (if purpose/characteristics insufficient for categorization)
  - Conducted before initial pivotal and/or initial FDA-authorization
  - Referenced in later studies as contributing to development/piloting

Pivotal RCT (one per study; utilized as anchor for determining other categories): in the *initial* target population/indication per first (earliest) identified FDA-authorization of the DTx, meeting at least one of the following:

- Study types/purposes: RCT explicitly reported as pivotal study per study record, other DTx study records, on DTx website, or in FDA documentation which evaluated DTx effectiveness or efficacy (as these terms are sometimes reported interchangeably)
- Characteristics relative to other studies/authorizations
  - Largest non-pilot RCT conducted before initial authorization
  - If multiple RCTs reported as pivotal for 1 DTx, we considered the pivotal trials as those with the larger sample size and most complete with available publications
  - Pivotal study for first DTx version of device (where predicate network traced back over long time periods with incremental development)

RWE/post-market: further evaluations including the *initial* target population/indication per first (earliest) identified FDA-authorization of the DTx for which the purpose was related to evaluations of DTx in practice and implementation, registry studies, and cost-effectiveness studies, as detailed below:

- Study types/purposes
  - Non-human
    - Algorithm assessment/evaluation using real-world data, which was not clearly related to initial development (which would be development/pilot) or development of new features (which would be DTx indication expansion/modification)
    - Cost-effectiveness based on modeling
  - Human: any study design with the following purposes:
    - Evaluations of DTx in practice, for example:
      - Implementation studies (including targeting specific, but already authorized populations, i.e., underprivileged groups)
      - Healthcare integration studies, quality improvement programs, service evaluations
      - Case-series and case-studies
      - FDA-mandated PAS, Postmarket Surveillance studies
      - Objectives related to evaluating DTx effectiveness in the strict context of RWE/pragmatic studies, for first DTx version of device (where predicate network traced back over long time periods with incremental development and/or non-clearly DTx versions)
    - Evaluations utilizing real-world data, for example:
      - Registry or manufacturer/insurance databases
      - Real-world data generated from software/apps

- Timing (if purpose/characteristics insufficient for categorization): following *commercial* availability; as some DTx, in particular, apps, were commercially available prior to initial FDA-authorization (including where first authorized outside the US), this category could include studies conducted prior to initial authorization

DTx indication expansion/modification: *outside initial* target population/indication and/or a *modified version of the DTx* per first (earliest) FDA-authorization, including for FDA labeling changes, of any type (development/pilot, pivotal RCT, or RWE/post-market), including:

- Study types/purposes
  - Non-human: algorithm assessment related to development of new features (algorithm refinements, modifications to software, AI and predictive model development)
  - Human: any study design with the following purposes:
    - Developing or evaluating new versions of or modifications to DTx
      - Testing updated versions or new features
      - Modifications to DTx, including use with new medications (e.g., diabetes related DTx) or cultural/language/country-specific adaptations
    - Evaluation in different population or for new indication not covered in initial authorization
      - Expanded age group only
      - New indication
        - On different disease/condition (e.g., cardiovascular effect of diabetes DTx)
        - In different disease/condition population (e.g., acute versus chronic pain; Type 1 versus Type 2 diabetes)
    - Evaluating new treatment modality
      - Prescription versus over-the-counter use
      - Stand-alone versus as adjunct therapy
    - Registries/real world data studies conducted solely in populations/versions or for indications not covered in the initial authorization of the DTx-version of the device
    - Explicitly reported in FDA documentation, other DTx study records, or on DTx website as supporting evidence in FDA-clearances for modifications to DTx, including those related to requests for labeling changes for modifications to DTx
- Timing (if purpose/characteristics insufficient for categorization): following initial authorization; as some DTx were evaluated simultaneously in different populations and/or authorized in other countries before authorization in the US, this category could include studies conducted prior to initial FDA-authorization

Unclear: ambiguity in which category most appropriate

- Modeling/algorithm studies where not possible to determine if purpose was initial or new version/feature development or for the purpose of real-world evaluation
- Case series/reports where not possible to determine if purpose was development or pilot/feasibility/preliminary or RWE/post-approval/cost-effectiveness
- Other: ambiguity in relation to type and timing of study, other studies, and FDA-authorization

In this categorization, the first study attribute of interest was the indication for use in the first FDA-authorization of the DTx. Less emphasis was placed on study design (as, for example, RCTs could be conducted in any of the classifications) and the declared study phase (as, for example, a pilot study could be conducted in the context of development/pilot, DTx indication expansion/modifications, or RWE/post-market implementation studies), as these characteristics alone cannot be used to clearly indicate the appropriate category, but must be considered in relation to the DTx evaluation/regulation ecosystem as a whole. Additionally, while it was a consideration, timing of studies in relation to other development/evaluation/regulatory events was not a concrete deciding factor, as, for example, DTx could be commercially available and generate RWE prior to authorization.

We considered a DTx to have an extensive evidence dossier if it had at least 1 study identified within each category (excluding unclear).

### Statistical analysis

In the initial descriptive analysis, we described included DTx, per their first (earliest) identified FDA-authorization, according to their characteristics and regulatory information. DTx characteristics reported include if a prescription was required, target disease/condition (smoking cessation; musculoskeletal health [muscle weakness, pain, recovery, re-education, training; performance enhancement; injuries prevention and rehabilitation; joint mobility, functional pathologies]; women’s health [birth control or period and fertility trackers, incontinence or pelvic floor dysfunction, alone or in combination with other disorders]; respiratory conditions [prescribed metered dose inhaler (MDI) medication users; patients unable to cough or clear secretions/mobilization of secretions and lung expansion therapy]; diabetes; arthritis or chronic pain [back, trunk, limbs]; child health [amblyopia, ADHD]; essential tremor; irritable bowel syndrome; sleep disorders [insomnia, nightmare]; cardiac diseases;, migraine), and device components (combined hardware/software; software which connects/can connect to other hardware; software/app only). The target age was categorized as children only (including ages 0-12 years old), adults only (including “adults” with age limit not specified or age limit at least 18 and older; where targeting “women”), children and adults (lower age bound includes children and adults, i.e., from 2-12 years and older), adolescents and adults (lower age bound includes adolescents and adults, i.e., from 13 years and older; intended for fertility/birth control purposes where age not specified), or unclear. Regulatory and predicate network information reported includes regulatory pathway (510(k), De Novo, PMA, HDE) and identifier, year, and whether (and timing of) additional FDA decisions/clearances were identified for DTx modifications.

Additionally, we described the studies identified evaluating these DTx overall, in terms of quantity, timing, and totality of evidence for each DTx (median, range studies per DTx), study type (RCT or non-randomized studies involving human subjects, animal, or modeling/simulation/in vitro studies), and classification. For studies involving human subjects, we additionally report study design, population size (median, range) and type, and primary outcome assessed, overall and by classification. For RCTs involving human subjects, we determine the availability and quantity of evidence for each DTx (median, range studies per DTx) and population size (median, range), overall and by classification.

Then, we conducted a comprehensive visual timeline review mapping the totality of evidence for the included DTx, highlighting DTx evaluations and FDA-decisions. First, we created comprehensive timelines of the evaluation and regulation of each DTx (dates all FDA-decisions and evaluation study start, completion, and publication/posting of results/protocol), by study classification. For one DTx, we provided a more thorough review including additional information related to study design, population size and characteristics (for DTx indication expansion/modification studies), and location.

Finally, we created several summary timelines to illustrate and compare the study periods for each study category relative to its first FDA-authorization, by DTx. We defined the study period using the earliest study start date and latest study completion date for the studies within each defined category for each DTx (e.g., development/pilot study period starting with the earliest development/pilot start and ending with the latest completion). In the first summary figure, we plotted the study periods for all categories together in a single plot, relative to the first FDA-authorization, by initial FDA-authorization pathway. Lastly, for study periods related to the first FDA-authorization (development/pilot, pivotal RCT, RWE/post-market), we plotted and described time (years; median, range) from these timepoints (study start, completion, and first publication/availability of results, separately) to/from the first FDA-authorization; these analyses were stratified by timing before or after the first authorization, overall and further stratified by FDA-approval pathway (510(k); PMA, dDe Novo, HDE), year of first FDA-authorization (2002-2019; 2020-2022) and DTx components (combined hardware/software; software which connects/can connect to other hardware+software/app only [software/app-only or with connection to hardware]). In the analyses, overall and stratified by FDA-authorization pathway, year of first FDA-authorization, and DTx components, we determined the number/percentage of DTx in each stratum with a pivotal RCT and other classifications.

R version 4.2.2 (R Project for Statistical Computing, Vienna, Austria) was utilized for analysis, with assistance from ChatGPT (version 4, 5, and 5.1) for writing code.

## Supplementary information

Supplementary Information

## Data Availability

The original data from the study are publicly available from the United States Food & Drug Administration (FDA), ClinicalTrials.gov, PubMed, and DTx websites. The datasets generated and/or analyzed during the current study are not publicly available as all the underlying data were extracted directly from publicly available sources, but are available from the corresponding author on reasonable request.

## Code availability

The R code (R version 4.2.2) used in this analysis is not publicly available but may be made available to qualified researchers on reasonable request from the corresponding author.
