# Supplementary material for: Mapping the evidence supporting FDA-authorized digital therapeutics, before and after authorization
Source: NPJ Digit Med. 2026 May 20;9:618. doi: 10.1038/s41746-026-02737-9 (PMC13458057; doi:10.1038/s41746-026-02737-9)
Supplement: Supplementary file 1 [file 41746_2026_2737_MOESM1_ESM.pdf]

# Supplementary information

**Table of Contents**

Table S1: FDA regulatory information and characteristics of predicates networks of FDA-authorized DTx ................ 2
Table S2: Predicates networks of FDA-authorized DTx ................................................................................... 3
Table S3: Availability and characteristics of unique studies of FDA-authorized DTx.................................................. 6
Table S4: RCTs involving human subjects of FDA-authorized DTx (N=143) .............................................................. 7
Table S5: Study population in studies involving human subjects of FDA-authorized DTx, overall and by study classification (N=412) ........................................................................................................................ 8
Table S6: Time to first study start, last study completion, and first publication of results for first FDA authorization of included DTx, by category of study purpose (N=45 DTx) ........................................................................... 9
Table S7: Time to first study start, last study completion, and first publication of results for first FDA authorization of included DTx, by category of study purpose and initial FDA authorization type ........................................................ 10
Table S8: Time to first study start, last study completion, and first publication of results for first FDA authorization of included DTx, by category of study purpose and DTx components............................................................................ 11
Table S9: Time to first study start, last study completion, and first publication of results for first FDA authorization of included DTx, by category of study purpose and year of initial DTx authorization..................................................... 12
Figure S1: Selection of FDA-authorized DTx with a decision from 2019-2022 ......................................................... 13
Figure S2: Flowchart for the selection of studies evaluating FDA-authorized DTx with a decision from 2019-2022.. 14
Figure S3: Time from first study start to FDA-authorization among studies related to the first authorized indication, by study classification ........................................................................................................................ 15
Figure S4: Time from last study completion to FDA-authorization among studies related to the first authorized indication, by study classification........................................................................................................... 17
Figure S5: Time from first publication/availability of results to FDA-authorization among studies related to the first authorized indication, by study classification ............................................................................................ 19
Figure S6: Time from first study start to FDA-authorization among studies related to the first authorized indication, by study classification, stratified by FDA-authorization pathway (510(k) versus PMA and De Novo)............................ 21
Figure S7: Time from last study completion to FDA-authorization among studies related to the first authorized indication, by study classification, stratified by FDA-authorization pathway (510(k) versus PMA and De Novo)...... 23
Figure S8: Time from first publication/availability of results to FDA-authorization among studies related to the first authorized indication, by study classification, stratified by FDA-authorization pathway (510(k) versus PMA and De Novo)........................................................................................................................................... 25
Figure S9: Time from first study start to FDA-authorization among studies related to the first authorized indication, by study classification, stratified by DTx components ......................................................................................... 27
Figure S10: Time from last study completion to FDA-authorization among studies related to the first authorized indication, by study classification, stratified by DTx components .................................................................. 29
Figure S11: Time from first publication/availability of results to FDA-authorization among studies related to the first authorized indication, by study classification, stratified by DTx components ............................................... 31
Figure S12: Time from first study start to FDA-authorization among studies related to the first authorized indication, by study classification, stratified by timing of first authorization (2002-2019 or 2020-2022) ..................................... 33
Figure S13: Time from last study completion to FDA-authorization among studies related to the first authorized indication, by study classification, stratified by timing of first authorization (2002-2019 or 2020-2022) .................... 35
Figure S14: Time from first publication/availability of results to FDA-authorization among studies related to the first authorized indication, by study classification, stratified by timing of first authorization (2002-2019 or 2020-2022) .. 37
Document S1: Totality of evidence for each DTx ......................................................................................... 39

| **Table S1: FDA regulatory information and characteristics of predicates networks of FDA-authorized DTx** | | |
|---|---|---|
| FDA regulatory information by DTx (n=45) | | n (%) |
| Initial FDA-authorization | 510(k) premarket notification[a] | 32 (71.1) |
| | De novo[b] | 10 (22.2) |
| | Pre-market approval (PMA)[c] | 3 (6.7) |
| Subsequent FDA clearance for DTx modification located | Yes | 26 (57.8) |
| | Time from first to second identified authorization (years); median (range) | 1.7 (0.0, 5.0) |
| | No | 19 (42.2) |
| Characteristics of predicate networks | | |
| FDA-authorizations identified | All FDA records identified in included DTx predicate networks (total) | 136 |
| | 510(k) clearances following initial authorization (total) | 91 |
| | FDA-authorizations per DTx; median (range) | 2 (1, 11) |
| Year subsequent FDA-clearance | 2006-2009 | 4 (4.4) |
| | 2010-2014 | 10 (11.0) |
| | 2015-2018 | 21 (23.1) |
| | 2019 | 8 (8.8) |
| | 2020 | 13 (14.3) |
| | 2021 | 14 (15.4) |
| | 2022 | 9 (9.9) |
| | 2023 | 9 (9.9) |
| | 2024 | 3 (3.3) |

[a]510(k) premarket notification is a simplified process for typically lower risk devices which can claim substantial equivalence to a previously approved predicate device.
[b]Pre-market approval (PMA) is rigorous process requiring proof of safety and effectiveness for higher risk devices.
[c]De Novo requests are intended for low/moderate risk devices without predicates, through an approval process less rigorous than the PMA pathway.

Abbreviations: digital therapeutic (DTx); United States Food & Drug Administration (FDA)

**Table S2: Predicates networks of FDA-authorized DTx**

| DTx | Authorization number[a,b,c] | Date (year-month-day) |
|---|---|---|
| Aluna | K193311 | 2020-03-25 |
| Assure WCD | P200037 | 2021-07-27 |
| Ava | K200163 | 2021-01-19 |
| Bigfoot Unity | K202145 | 2021-05-07 |
| | K222280 | 2023-03-01 |
| Biofeedback MNS | K202648 | 2020-12-08 |
| | K220161 | 2022-07-20 |
| Biwaze Cough | K191912 | 2020-03-27 |
| BlueStar | K100066 | 2010-07-15 |
| | K112370 | 2011-10-14 |
| | K120314 | 2012-02-24 |
| | K141273 | 2014-07-08 |
| | K162225 | 2016-11-22 |
| | K162532 | 2017-01-12 |
| | K190013 | 2019-11-04 |
| | K193654 | 2020-04-07 |
| | K203434 | 2021-09-08 |
| | K230813 | 2023-07-28 |
| | K222888 | 2023-08-11 |
| Breathesuite | K203155 | 2021-09-17 |
| Cala | DEN170028 | 2018-04-26 |
| | K182706 | 2018-10-25 |
| | K203288 | 2021-10-05 |
| | K222237 | 2022-11-22 |
| Capmedic | K183586 | 2019-09-13 |
| Cionic | K213622 | 2022-02-14 |
| | K221823 | 2022-07-21 |
| Clue | K193330 | 2021-02-18 |
| Control-IQ | DEN190034 | 2019-12-13 |
| | K200467 | 2020-06-16 |
| | K232382 | 2023-11-03 |
| Curesight | K221375 | 2022-09-29 |
| D-Nav | K181916 | 2019-02-04 |
| EaseVRx | DEN210014 | 2021-11-16 |
| Endeavor | DEN200026 | 2020-06-15 |
| | K231337 | 2023-12-13 |
| | K233496 | 2024-06-14 |
| Flyte | K200409 | 2020-07-10 |
| | K210764 | 2021-04-13 |
| | K212655 | 2021-09-21 |
| | K233362 | 2023-12-29 |
| | K240805 | 2024-08-01 |
| Icoquit | K211918 | 2021-12-21 |
| Inpen | K160629 | 2016-07-26 |
| | K181327 | 2018-07-06 |
| | K192841 | 2020-02-10 |
| | K190487 | 2020-02-18 |
| | K201337 | 2020-06-18 |
| Insulia | K161433 | 2016-11-09 |
| | K170669 | 2017-06-19 |
| | K172177 | 2017-11-07 |
| | K202596 | 2021-06-04 |
| L100 Go/L360 Thigh | K022776 | 2002-09-11 |
| | K053468 | 2006-07-07 |
| | K080219 | 2008-02-28 |

| | | |
|---|---|---|
| | K103343 | 2011-04-29 |
| | K120853 | 2012-04-20 |
| | K122784 | 2012-12-27 |
| | K162407 | 2017-01-27 |
| | K173682 | 2018-03-09 |
| | K190285 | 2019-03-14 |
| | K191587 | 2019-09-12 |
| | K200262 | 2020-03-04 |
| Leva | K133990 | 2014-10-09 |
| | K180637 | 2018-04-04 |
| | K192270 | 2019-11-22 |
| | K212495 | 2021-09-08 |
| | K213913 | 2022-06-30 |
| Levator Elite | K213043 | 2022-02-25 |
| Luminopia | DEN210005 | 2021-10-20 |
| | K221659 | 2022-11-04 |
| Parallel | DEN200029 | 2020-11-25 |
| | K211372 | 2021-06-02 |
| Maximus | K124032 | 2013-04-25 |
| | K151689 | 2016-03-17 |
| | K192143 | 2020-02-14 |
| | K200988 | 2020-05-26 |
| Intellihab/Motive | K150413 | 2015-04-02 |
| | K163067 | 2016-12-01 |
| | K210604 | 2021-06-03 |
| | K220738 | 2022-05-12 |
| Mstim Drop | K202110 | 2021-11-05 |
| Myocycle | K170132 | 2017-04-25 |
| | K213925 | 2022-04-25 |
| Nalu | K183047 | 2019-03-22 |
| | K183579 | 2019-03-29 |
| | K191435 | 2019-09-06 |
| | K201618 | 2020-07-15 |
| | K202274 | 2020-11-09 |
| | K203547 | 2021-03-25 |
| | K221376 | 2022-06-10 |
| Natural Cycles | DEN170052 | 2018-08-10 |
| | K202897 | 2021-06-24 |
| | K231274 | 2023-08-24 |
| | K241006 | 2024-07-11 |
| Nerivio | DEN180059 | 2019-05-20 |
| | K201824 | 2020-10-23 |
| | K203181 | 2021-01-22 |
| | K223169 | 2023-02-06 |
| | K232152 | 2023-11-08 |
| Neurolutions | DEN200046 | 2021-04-23 |
| NightWare | DEN200033 | 2020-11-06 |
| OneTouch | K024194 | 2003-02-07 |
| | K053529 | 2006-01-17 |
| | K072543 | 2007-10-05 |
| | K093745 | 2011-02-11 |
| | K110637 | 2011-09-07 |
| | K131363 | 2013-08-30 |
| | K150214 | 2015-07-31 |
| | K193475 | 2020-02-14 |
| Optimizer | P180036 | 2019-03-21 |
| Pivot | K171408 | 2017-09-29 |
| | K201206 | 2021-06-30 |

| | | |
|---|---|---|
| Powerdot | K150078 | 2015-09-30 |
| | K172876 | 2017-12-04 |
| | K181759 | 2018-11-09 |
| | K210269 | 2021-02-23 |
| | K210938 | 2021-07-30 |
| Propeller | K121609 | 2012-07-02 |
| | K140638 | 2014-05-02 |
| | K142960 | 2015-03-05 |
| | K142516 | 2015-05-15 |
| | K143671 | 2015-06-19 |
| | K152482 | 2016-03-04 |
| | K152882 | 2016-03-08 |
| | K161454 | 2016-11-01 |
| | K180770 | 2018-08-01 |
| | K192724 | 2020-03-26 |
| Regulora | K211463 | 2021-11-24 |
| Saluda | P190002 | 2022-02-28 |
| Shoulder Pacemaker | K210674 | 2021-08-24 |
| | K220994 | 2022-10-14 |
| Somryst | K191716 | 2020-03-23 |
| Vera | K150462 | 2015-10-30 |
| | K182333 | 2019-04-09 |
| | K192474 | 2019-10-10 |

[a]510(k) premarket notification is a simplified process for typically lower risk devices which can claim substantial equivalence to a previously approved predicate device. The authorization number for these clearances starts with a "K."
[b]Pre-market approval (PMA) is rigorous process requiring proof of safety and effectiveness for higher risk devices. The authorization number for these clearances starts with a "P."
[c]De Novo requests are intended for low/moderate risk devices without predicates, through an approval process less rigorous than the PMA pathway. The authorization number for these clearances starts with "DEN."

Abbreviations: digital therapeutic (DTx); United States Food & Drug Administration (FDA)

| Table S3: Availability and characteristics of unique studies of FDA-authorized DTx | | |
|---|---|---|
| Studies/evidence by DTx (N=45 DTx) | | n ( %) |
| Study availability | DTx with at least 1 study | 40 (88.9) |
| | DTx without a study identified | 5 (11.1) |
| | Studies per DTx; median (range) | 7 (0, 73) |
| Totality of evidence | DTx with extensive evidence dossiers[a] | 14 (31.1) |
| | DTx with at least one classification missing | 31 (68.9) |
| | DTx with prior development/pilot study and pivotal studies[b] | 5 (11.1) |
| Timing of evidence relative to first authorization | Long period of development/evaluation (>10 years)[c] | 6 (13.3) |
| | No studies conducted before first authorization[d] | 13 (28.9) |
| | No results published before first authorization | 24 (53.3) |
| Study characteristics (N=436 unique studies) | | |
| Study type | Human | 412 (95.5) |
| | Randomized trials | 143 (32.8) |
| | Non-randomized studies | 269 (61.7) |
| | Non-human | 24 (5.5) |
| | Animal | 8 (1.8) |
| | Modeling/simulation/in vitro[e] | 16 (3.7) |
| Study classification | Development / pilot | 132 (30.3) |
| | Pivotal RCT | 21 (4.8) |
| | RWE / post-market | 127 (29.1) |
| | DTx indication expansion / modification studies | 145 (33.3) |
| | Unclear[f] | 11 (2.5) |

[a]At least 1 study identified within each category (development/pilot, pivotal RCT, RWE/post-market, DTx indication expansion/modification studies)
[b]Development/pilot and pivotal studies prior to first authorization but without further RWE/post-market and/or DTx indication expansion/modification studies
[c]DTx with long periods of development/evaluation: first study start and/or publication/results available >10 years before first authorization
[d]Includes DTx without studies identified and those without a study with a start, completion, or publication prior to first FDA-authorization
[e]Modeling/simulation/in vitro studies: n=15 algorithm, model development/validation/optimization, simulation or in silico; n=1 in vitro (modeling of inhaler aerosol deposition)
[f]Not classified: n=11 (modeling/algorithm [n=5] or case series/reports with unclear purpose [n=3]; ambiguity related to type and timing of study, other studies, and FDA-authorization [n=3])

Abbreviations: digital therapeutic (DTx); United States Food & Drug Administration (FDA); randomized controlled trial (RCT); real world evidence (RWE)

| Table S4: RCTs involving human subjects of FDA-authorized DTx (N=143) | | | | | | |
|---|---|---|---|---|---|---|
| | Development / Pilot (n=45) | Pivotal RCT (n=21) | RWE / Post-market (n=18) | DTx Indication Modification (n=58) | Unclear (n=1) | TOTAL (n=143) |
| **RCTs per DTx (n=45 DTx)** | | | | | | |
| DTx with an RCT (n; %) | 22 (48.9) | 21 (46.7) | 11 (24.4) | 18 (42.0) | 1 (2.2) | 31 (68.9) |
| Median (range) RCTs | 1 (1, 12) | 1 (1, 1) | 1 (1, 4) | 2 (1, 15) | 1 (1, 1) | 3 (1, 31) |
| DTx no RCT (n; %) | 23 (51.1) | 24 (53.3) | 34 (75.6) | 27 (60.0) | 44 (97.8) | 14 (31.1) |
| **Patients included in RCTs** | | | | | | |
| Median (range) | 48 (9, 1000) | 188 (59, 558) | 119 (13, 667) | 69.5 (6, 1500) | 81 (81, 81) | 82 (6, 1500) |

Abbreviations: digital therapeutic (DTx); United States Food & Drug Administration (FDA); randomized controlled trial (RCT); real world evidence (RWE)

| Table S5: Study population[a] in studies involving human subjects[b] of FDA-authorized DTx, overall and by study classification (N=412) | | | | | | |
|---|---|---|---|---|---|---|
| | Development / Pilot | Pivotal RCT | RWE / Post-market | DTx Indication Modification | Unclear | TOTAL[c] |
| **Patient** | | | | | | |
| Studies | n=118 | n=21 | n=124 | n=138 | n=6 | n=407 |
| Participants, median (range) | 30 (1, 3268) | 188 (59, 558) | 199.5 (2, 144000) | 50 (1, 56715) | 42.5 (1, 378694) | 66 (1, 378694) |
| **Provider** | | | | | | |
| Studies | n=1 | n=0 | n=4[d] | n=5 | n=0 | n=10[d] |
| Participants, median (range) | 26 (26, 26) | --- | 12 (10, 15) | 22 (8, 355) | --- | 14 (8, 355) |
| **Other (parent, partner)** | | | | | | |
| Studies | n=0 | n=0 | n=2[d] | n=1 | n=0 | n=3[d] |
| Participants, median (range) | --- | --- | 9 (6, 12) | 120 (120, 120) | --- | 12 (6, 120) |

[a]Where number of participants reported as “>” a number, took value reported; for studies with multiple participant types (e.g., patients and providers), where one type participant total specified and another type not, took value reported

[b]24 non-human studies were also included; 8 which were animal studies and 16 which were modeling/algorithm/in vitro studies

[c]Studies with more than 1 type of participant (for example, patients and providers) were included in all relevant categories, thus the total column adds to greater than the number of studies included.

[d]n=1 RWE / Post-market study where # participants (providers) unclear not included in table

Abbreviations: digital therapeutic (DTx); United States Food & Drug Administration (FDA); randomized controlled trial (RCT); real world evidence (RWE)

| Table S6: Time to first study start, last study completion, and first publication of results for first FDA authorization of included DTx, by category of study purpose (N=45 DTx) | | | | | |
|---|---|---|---|---|---|
| | **Pre-authorization** | | **Post-authorization** | | **DTx without studies/data[a] (n)** |
| To first study start | DTx with studies (n) | Years before authorization (median [range]) | DTx with studies (n) | Years after authorization (median [range]) | |
| Development/pilot study | 26 | 4.7 (0.7, 17.6) | 5 | 2.8 (1.8, 13.7) | 14 |
| Pivotal RCT | 18 | 2.8 (1.1, 14.1) | 3 | 6.0 (4.5, 7.6) | 24 |
| RWE/post-market | 12 | 3.2 (0.3, 13.7) | 14 | 1.1 (0.0, 8.9) | 19 |
| To last study completion | | | | | |
| Development/pilot study | 19 | 2.9 (0.2, 10.7) | 12 | 3.6 (0.0, 14.4) | 14 |
| Pivotal RCT | 14 | 1.0 (0.0, 5.5) | 7 | 3.8 (0.5, 10.4) | 24 |
| RWE/post-market | 1 | 1.4 (1.4, 1.4) | 25 | 5.2 (0.3, 19.5) | 19 |
| To first results publication/posting | | | | | |
| Development/pilot study | 21 | 3.9 (0.0, 16.3) | 10 | 2.5 (0.1, 15.3) | 14 |
| Pivotal RCT | 9 | 1.3 (0.0, 8.1) | 12 | 2.3 (0.1, 10.6) | 24 |
| RWE/post-market | 6 | 2.8 (0.2, 8.9) | 16 | 2.8 (0.7, 13.2) | 23 |

[a]DTx without studies/data can include those with missing data for study start and completion dates (frequently missing for studies with publications only). Additionally, first results publication/posting can include studies identified in trial registries which do not yet have publications.

Abbreviations: digital therapeutic (DTx); United States Food & Drug Administration (FDA); randomized controlled trial (RCT); real world evidence (RWE)

**Table S7: Time to first study start, last study completion, and first publication of results for first FDA authorization of included DTx, by category of study purpose and initial FDA authorization type**

| | Pre-authorization | | Post-authorization | | **DTx without studies/ data[a] (n)** |
|---|---|---|---|---|---|
| **510(k) premarket notification (n=32)** | | | | | |
| To first study start | DTx with studies (n) | Years before authorization (median [range]) | DTx with studies (n) | Years after authorization (median [range]) | |
| Development/pilot study | 15 | 4.6 (0.7, 13.5) | 5 | 2.8 (1.8, 13.7) | 12 |
| Pivotal RCT | 8 | 3.1 (1.1, 8.5) | 3 | 6.0 (4.5, 7.6) | 21 |
| RWE/post-market | 8 | 2.5 (0.3, 11.7) | 8 | 1.3 (0.0, 8.9) | 16 |
| To last study completion | | | | | |
| Development/pilot study | 11 | 4.5 (0.2, 10.7) | 9 | 3.3 (0.0, 14.4) | 12 |
| Pivotal RCT | 6 | 1.1 (0.1, 5.5) | 5 | 5.7 (0.8, 10.4) | 21 |
| RWE/post-market | 1 | 1.4 (1.4, 1.4) | 15 | 6.0 (0.3, 19.5) | 16 |
| To first results publication/posting | | | | | |
| Development/pilot study | 10 | 4.1 (1.3, 12.2) | 9 | 3.7 (0.1, 15.3) | 13 |
| Pivotal RCT | 1 | 3.3 (3.3, 3.3) | 10 | 2.5 (0.1, 10.6) | 21 |
| RWE/post-market | 3 | 3.4 (2.4, 8.9) | 12 | 3.7 (1.0, 13.2) | 17 |
| **PMA or De Novo (n=13)** | | | | | |
| To first study start | | | | | |
| Development/pilot study | 11 | 7.2 (1.7, 17.6) | --- | --- | 2 |
| Pivotal RCT | 10 | 2.4 (1.3, 14.1) | --- | --- | 3 |
| RWE/post-market | 4 | 4.2 (2.1, 13.7) | 6 | 0.5 (0.1, 2.0) | 3 |
| To last study completion | | | | | |
| Development/pilot study | 8 | 1.8 (0.4, 9.2) | 3 | 3.9 (2.2, 5.3) | 2 |
| Pivotal RCT | 8 | 1.0 (0.0, 4.7) | 2 | 2.2 (0.5, 3.8) | 3 |
| RWE/post-market | --- | --- | 10 | 5.2 (2.6, 7.5) | 3 |
| To first results publication/posting | | | | | |
| Development/pilot study | 11 | 3.9 (0.0, 16.3) | 1 | 0.6 (0.6, 0.6) | 1 |
| Pivotal RCT | 8 | 1.0 (0.0, 8.1) | 2 | 1.5 (0.8, 2.2) | 3 |
| RWE/post-market | 3 | 2.4 (0.2, 3.2) | 4 | 1.4 (0.7, 3.0) | 6 |

[a]DTx without studies/data can include those with missing data for study start and completion dates (frequently missing for studies with publications only). Additionally, first results publication/posting can include studies identified in trial registries which do not yet have publications.

Abbreviations: digital therapeutic (DTx); United States Food & Drug Administration (FDA); Pre-market approval (PMA); randomized controlled trial (RCT); real world evidence (RWE)

**Table S8: Time to first study start, last study completion, and first publication of results for first FDA authorization of included DTx, by category of study purpose and DTx components**

| | Pre-authorization | | Post-authorization | | DTx without studies/data[a] (n) |
|---|---|---|---|---|---|
| **Combined hardware/software DTx (n=32 DTx)** | | | | | |
| To first study start | DTx with studies (n) | Years before authorization (median [range]) | DTx with studies (n) | Years after authorization (median [range]) | |
| Development/pilot study | 16 | 4.3 (0.7, 17.5) | 5 | 2.8 (1.8, 13.7) | 11 |
| Pivotal RCT | 8 | 2.4 (1.1, 14.1) | 3 | 6.0 (4.5, 7.6) | 21 |
| RWE/post-market | 6 | 1.7 (0.3, 13.7) | 9 | 1.2 (0.0, 8.9) | 17 |
| To last study completion | | | | | |
| Development/pilot study | 10 | 1.8 (0.2, 5.4) | 11 | 3.3 (0.0, 14.4) | 11 |
| Pivotal RCT | 6 | 0.5 (0.0, 1.5) | 5 | 5.7 (0.5, 10.4) | 21 |
| RWE/post-market | --- | --- | 15 | 5.8 (0.3, 19.5) | 17 |
| To first results publication/posting | | | | | |
| Development/pilot study | 10 | 3.2 (0.0, 16.3) | 10 | 2.5 (0.1, 15.3) | 12 |
| Pivotal RCT | 4 | 1.5 (0.0, 8.1) | 7 | 3.6 (0.1, 10.6) | 21 |
| RWE/post-market | 2 | 1.7 (0.2, 3.2) | 11 | 2.7 (1.0, 13.2) | 19 |
| **Software/app only or with connection to hardware (n=13 DTx)** | | | | | |
| To first study start | | | | | |
| Development/pilot study | 10 | 7.7 (1.7, 17.6) | --- | --- | 3 |
| Pivotal RCT | 10 | 3.4 (1.3, 8.5) | --- | --- | 3 |
| RWE/post-market | 6 | 6.0 (0.5, 11.7) | 5 | 0.9 (0.1, 2.0) | 2 |
| To last study completion | | | | | |
| Development/pilot study | 9 | 4.5 (0.4, 10.7) | 1 | 5.3 (5.3, 5.3) | 3 |
| Pivotal RCT | 8 | 2.2 (0.1, 5.5) | 2 | 2.5 (1.1, 3.8) | 3 |
| RWE/post-market | 1 | 1.4 (1.4, 1.4) | 10 | 4.9 (2.3, 11.5) | 2 |
| To first results publication/posting | | | | | |
| Development/pilot study | 11 | 6.8 (1.3, 12.2) | --- | --- | 2 |
| Pivotal RCT | 5 | 1.3 (0.1, 3.3) | 5 | 1 (0.1, 2.6) | 3 |
| RWE/post-market | 4 | 2.9 (2.4, 8.9) | 5 | 3 (0.7, 4.2) | 4 |

[a]DTx without studies/data can include those with missing data for study start and completion dates (frequently missing for studies with publications only). Additionally, first results publication/posting can include studies identified in trial registries which do not yet have publications.

Abbreviations: digital therapeutic (DTx); United States Food & Drug Administration (FDA); randomized controlled trial (RCT); real world evidence (RWE); mobile application (app)

| **Table S9: Time to first study start, last study completion, and first publication of results for first FDA authorization of included DTx, by category of study purpose and year of initial DTx authorization** | | | | | |
|---|---|---|---|---|---|
| **First authorized 2002-2019 (n=21)** | | | | | |
| | **Pre-authorization** | | **Post-authorization** | | **DTx without studies/ data[a] (n)** |
| To first study start | DTx with studies (n) | Years before authorization (median [range]) | DTx with studies (n) | Years after authorization (median [range]) | |
| Development/pilot study | 9 | 4.6 (2.4, 17.5) | 5 | 2.8 (1.8, 13.7) | 7 |
| Pivotal RCT | 7 | 2.1 (1.4, 14.1) | 3 | 6.0 (4.5, 7.6) | 11 |
| RWE/post-market | 7 | 3.8 (0.3, 13.7) | 8 | 1.1 (0.0, 8.9) | 6 |
| To last study completion | | | | | |
| Development/pilot study | 8 | 2.6 (0.4, 7.9) | 6 | 6.8 (2.2, 14.4) | 7 |
| Pivotal RCT | 6 | 1.1 (0.0, 3.5) | 4 | 6.3 (1.1, 10.4) | 11 |
| RWE/post-market | --- | --- | 15 | 6.1 (1.2, 19.5) | 6 |
| To first results publication/posting | | | | | |
| Development/pilot study | 10 | 2.8 (0.0, 16.3) | 6 | 4.6 (1.2, 15.3) | 5 |
| Pivotal RCT | 3 | 0.2 (0.0, 8.1) | 7 | 2.6 (0.1, 10.6) | 11 |
| RWE/post-market | 3 | 3.2 (2.4, 3.4) | 12 | 3.7 (0.7, 13.2) | 6 |
| **First authorized 2020-2022 (n=24)** | | | | | |
| To first study start | | | | | |
| Development/pilot study | 17 | 4.9 (0.7, 17.6) | --- | --- | 7 |
| Pivotal RCT | 11 | 2.8 (1.1, 8.5) | --- | --- | 13 |
| RWE/post-market | 5 | 2.7 (0.7, 11.7) | 6 | 0.9 (0.3, 2.0) | 13 |
| To last study completion | | | | | |
| Development/pilot study | 11 | 4.4 (0.2, 10.7) | 6 | 1.4 (0.0, 5.3) | 7 |
| Pivotal RCT | 8 | 1.0 (0.1, 5.5) | 3 | 0.8 (0.5, 3.8) | 13 |
| RWE/post-market | 1 | 1.4 (1.4, 1.4) | 10 | 3.6 (0.3, 5.8) | 13 |
| To first results publication/posting | | | | | |
| Development/pilot study | 11 | 4.4 (1.4, 12.2) | 4 | 0.3 (0.1, 0.6) | 9 |
| Pivotal RCT | 6 | 1.5 (0.1, 3.3) | 5 | 2.2 (0.1, 3.6) | 13 |
| RWE/post-market | 3 | 2.4 (0.2, 8.9) | 4 | 1.8 (1.0, 3.0) | 17 |

[a]DTx without studies/data can include those with missing data for study start and completion dates (frequently missing for studies with publications only). Additionally, first results publication/posting can include studies identified in trial registries which do not yet have publications.

Abbreviations: digital therapeutic (DTx); United States Food & Drug Administration (FDA); randomized controlled trial (RCT); real world evidence (RWE)

**Figure S1: Selection of FDA-authorized DTx with a decision from 2019-2022**

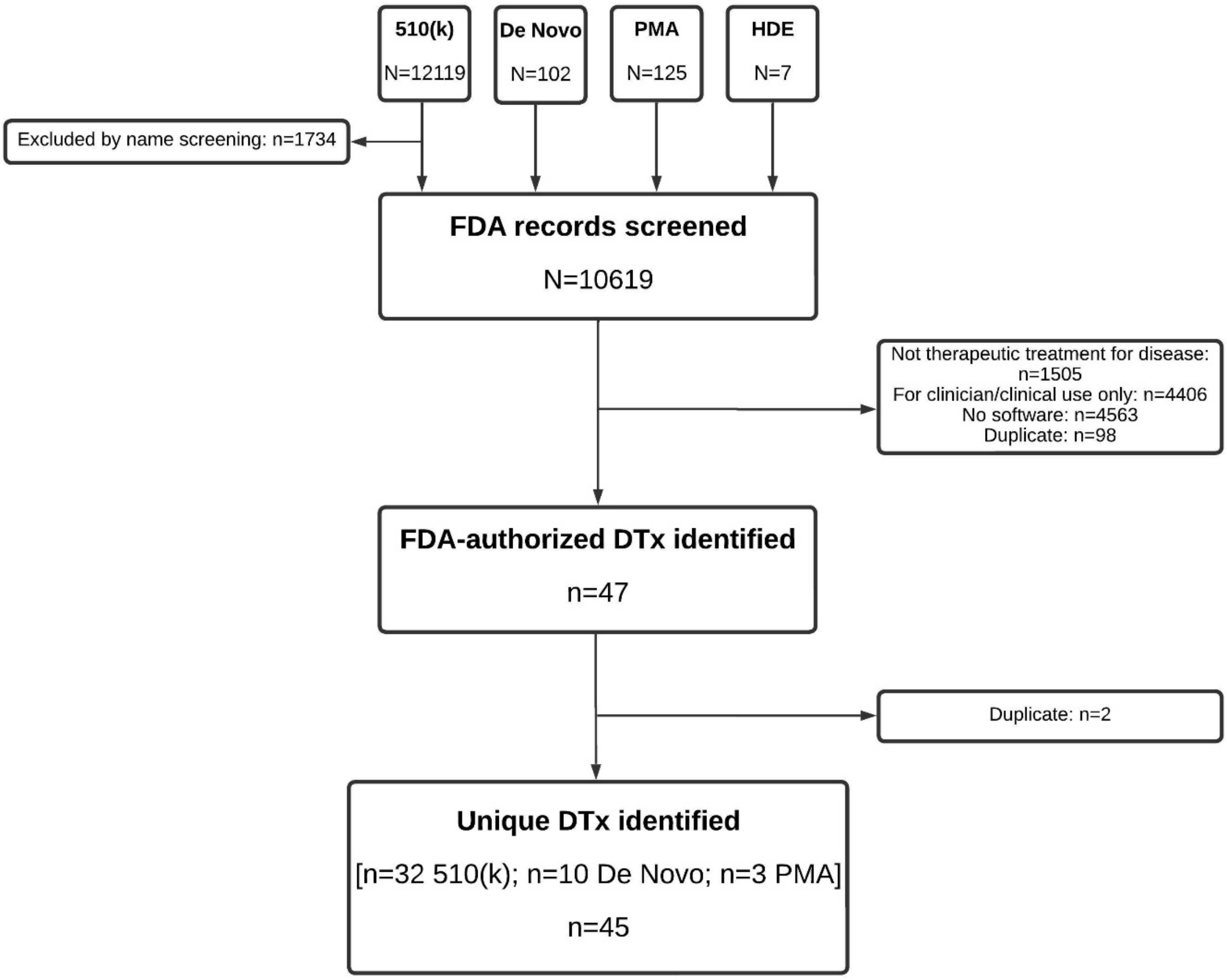


Legend: During the predicate review, two sets of two DTx each, which were authorized under different names, had common predicate networks and were therefore considered as "duplicates." In the first set, one DTx (IntelliHab system, intended for prescription use) was the named predicate of the second (Motive Knee Wrap, intended for over-the-counter use). In the second set (L100 Go System and L360 Thigh System), both reference a common predicate device (L300 Go System) and are subsets of that device (L100 Go System is a lower leg cuff only; L360 Thigh System is the Thigh Standalone configuration).

Abbreviations: digital therapeutic (DTx); United States Food & Drug Administration (FDA); Premarket Notification (510(k)), Premarket Approval (PMA), Humanitarian Device Exemption (HDE)

**Figure S2: Flowchart for the selection of studies evaluating FDA-authorized DTx with a decision from 2019-2022**

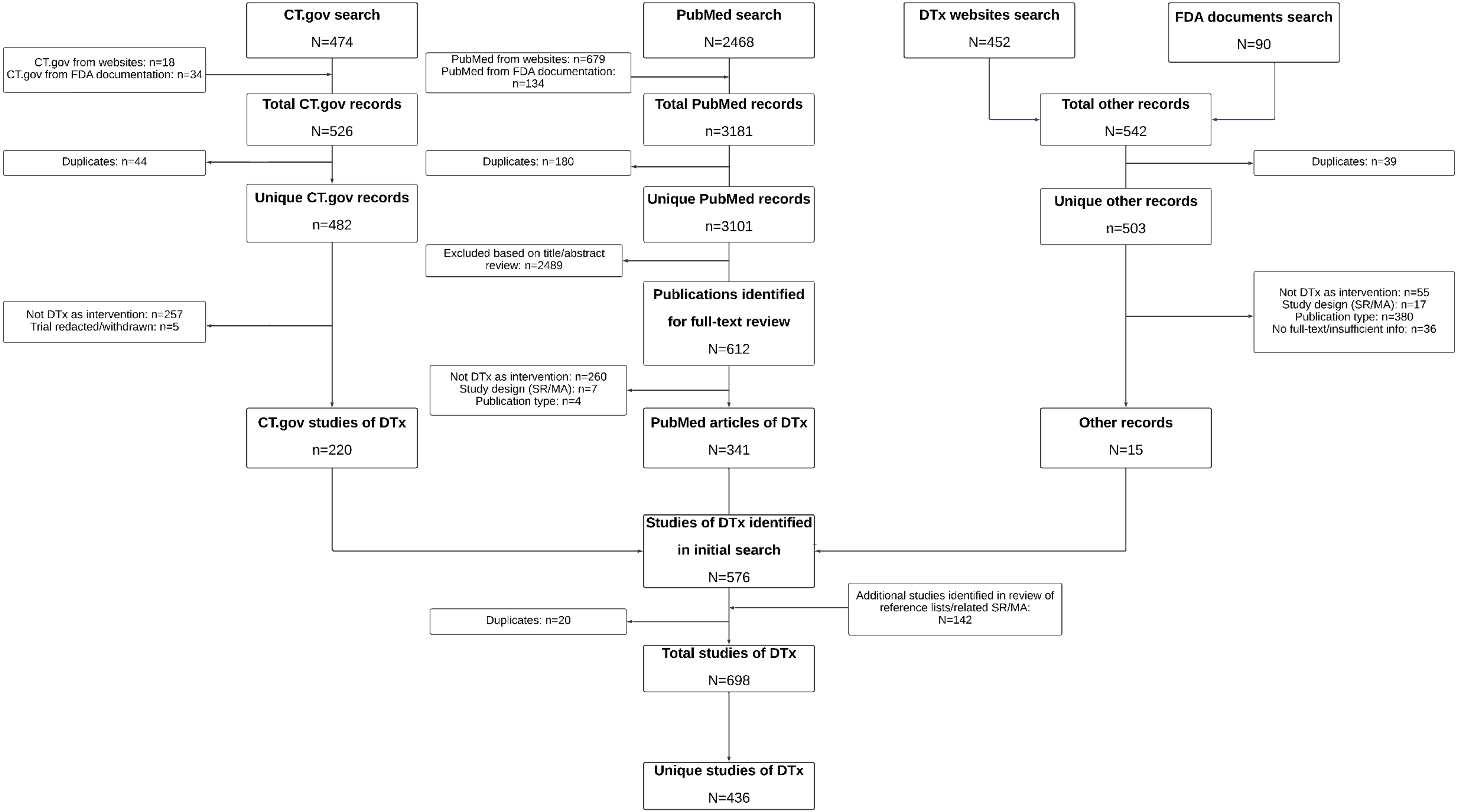


Legend: During the predicate review, two sets of two DTx each (IntelliHab system and Motive Knee Wrap; L100 Go System and L360 Thigh System), which were authorized under different names, had common predicate networks and were therefore considered as "duplicates." In the literature review, searches were conducted using for all 4 devices separately to ensure relevant records were located, then the retrieved studies were de-duplicated at the end of the search process.

Abbreviations: clinicaltrials.gov (CT.gov); digital therapeutic (DTx); United States Food & Drug Administration (FDA); systematic review/meta-analysis (SR/MA)

**Figure S3: Time from first study start to FDA-authorization among studies related to the first authorized indication, by study classification**

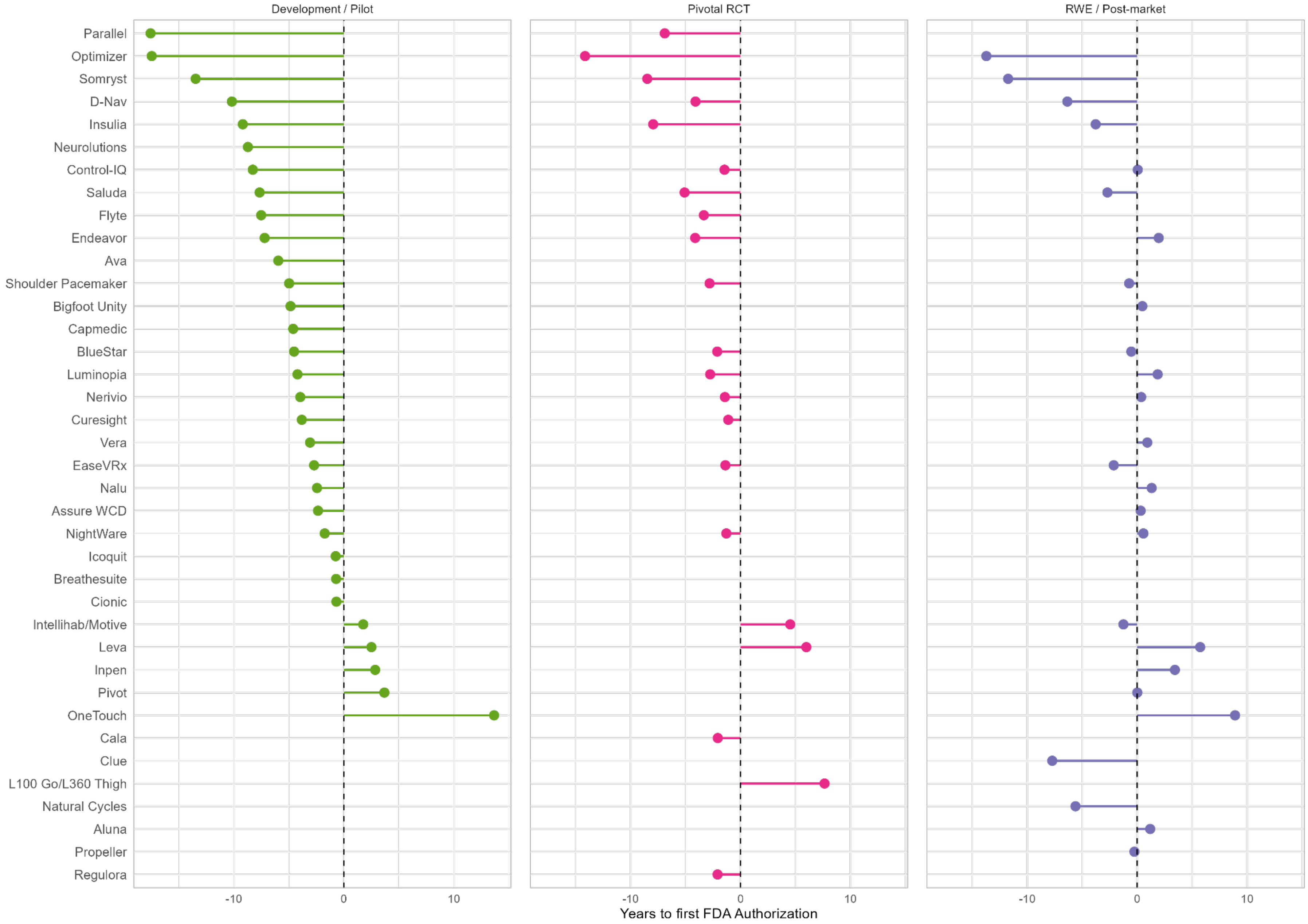

Legend: The start of the study period for evaluations of each DTx within each defined category (e.g., development/pilot study period starting with the earliest development/pilot start) are illustrated, in relation to the first FDA-authorization (set as time 0), for studies related to the indication for use in the first FDA-authorization. These categories were characterized as, briefly:

*Development/pilot*: in initial target population/indication per first FDA-authorization, and focusing on DTx development and/or pilot studies in preparation for the pivotal trial
*Pivotal RCT*: in initial target population/indication per first FDA-authorization
*RWE/post-market*: in initial target population/indication per first FDA-authorization and using real-world data, focusing on evaluation of DTx in practice, and/or following commercial availability

DTx with no studies/publications (n=5 DTx) or only DTx indication expansion/modification studies (n=2 DTx) and studies not related to the first indication for use (n=145 DTx indication expansion/modification studies) or with unclear classification (n=11) were not included in figure. Development/pilot (n=26 DTx; median 4.7, range 0.7-17.6 years prior) and pivotal RCT (n=18 DTx; median 2.8, range 1.1-14.1 years prior) periods tended to start prior to first authorization. Conversely, the timing of start of RWE/post-market were more heterogeneous, with some of these studies starting prior to authorization (n=12 DTx; median 3.2, range 0.3-13.7 years prior) and others after (n=14 DTx; median 1.1, range 0.0-8.9 years after) due to simultaneous evaluation in multiple countries and the commercial availability of the DTx prior to FDA-authorization. For all study periods across categories, the length of time varied greatly between DTx.

Abbreviations: digital therapeutic (DTx); United States Food & Drug Administration (FDA); randomized controlled trial (RCT); real world evidence (RWE)

**Figure S4: Time from last study completion to FDA-authorization among studies related to the first authorized indication, by study classification**

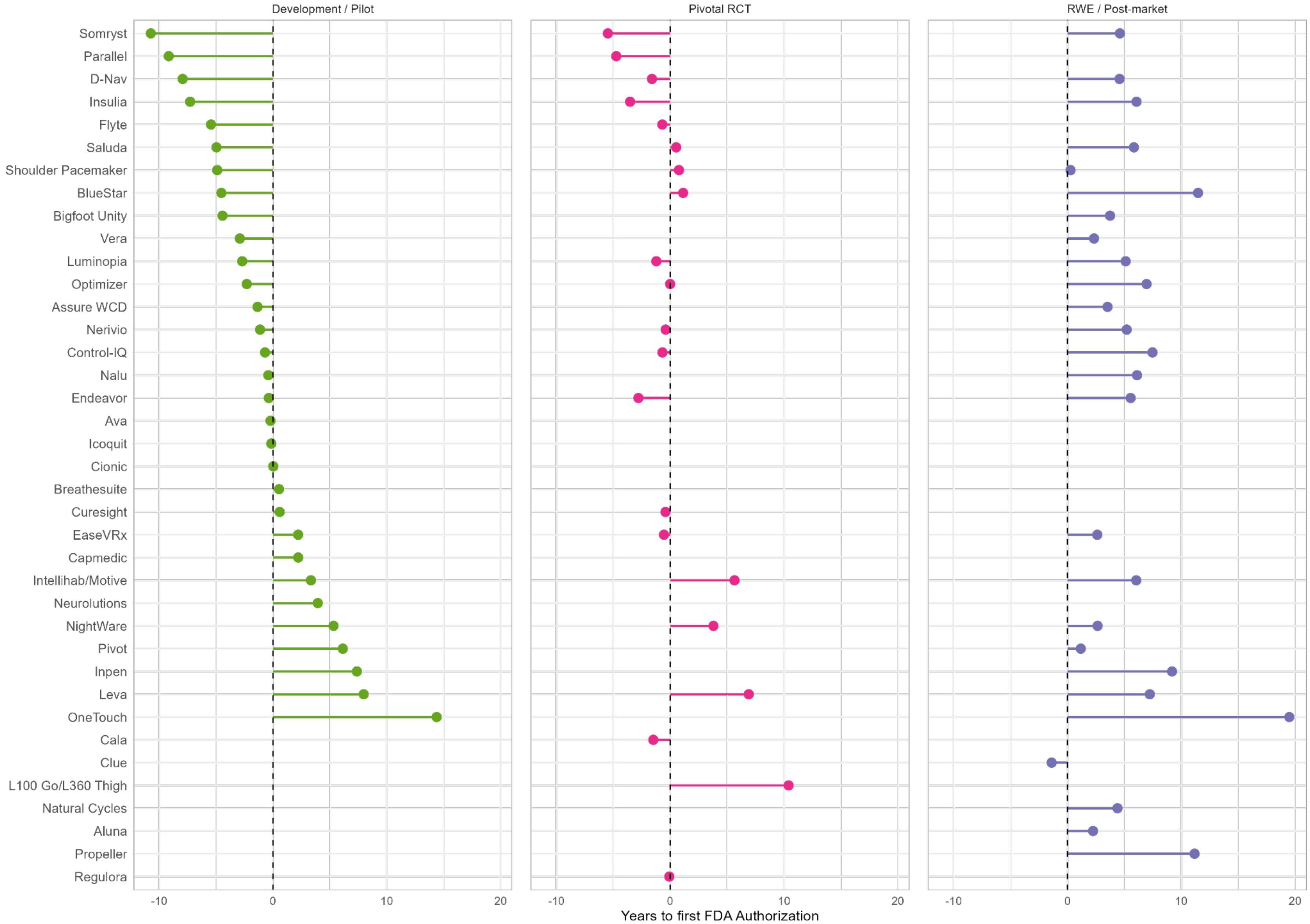

Legend: The end of the study period for evaluations of each DTx within each defined category (e.g., development/pilot study period ending with the latest development/pilot completion) are illustrated, in relation to the first FDA-authorization (set as time 0), for studies related to the indication for use in the first FDA-authorization. These categories were characterized as, briefly:

*Development/pilot*: in initial target population/indication per first FDA-authorization, and focusing on DTx development and/or pilot studies in preparation for the pivotal trial
*Pivotal RCT*: in initial target population/indication per first FDA-authorization
*RWE/post-market*: in initial target population/indication per first FDA-authorization and using real-world data, focusing on evaluation of DTx in practice, and/or following commercial availability

DTx with no studies/publications (n=5 DTx) or only DTx indication expansion/modification studies (n=2 DTx) and studies not related to the first indication for use (n=145 DTx indication expansion/modification studies) or with unclear classification (n=11) were not included in figure. Almost all RWE/post-market (n=25 DTx; median 5.2, range 0.3-19.5 years after) study periods ended after first FDA-authorization. Completion of the study periods were more mixed for development/pilot (n=19 DTx; median 2.9, range 0.2-10.7 years prior; n=12 DTx; median 3.6, range 0.0-14.4 years after) and pivotal RCTs (n=14 DTx; median 1.0, range 0.0-5.5 years prior; n=7 DTx; median 3.8, range 0.5-10.4 years after). For all study periods across categories, the length of time varied greatly between DTx.

Abbreviations: digital therapeutic (DTx); United States Food & Drug Administration (FDA); randomized controlled trial (RCT); real world evidence (RWE)

**Figure S5: Time from first publication/availability of results to FDA-authorization among studies related to the first authorized indication, by study classification**

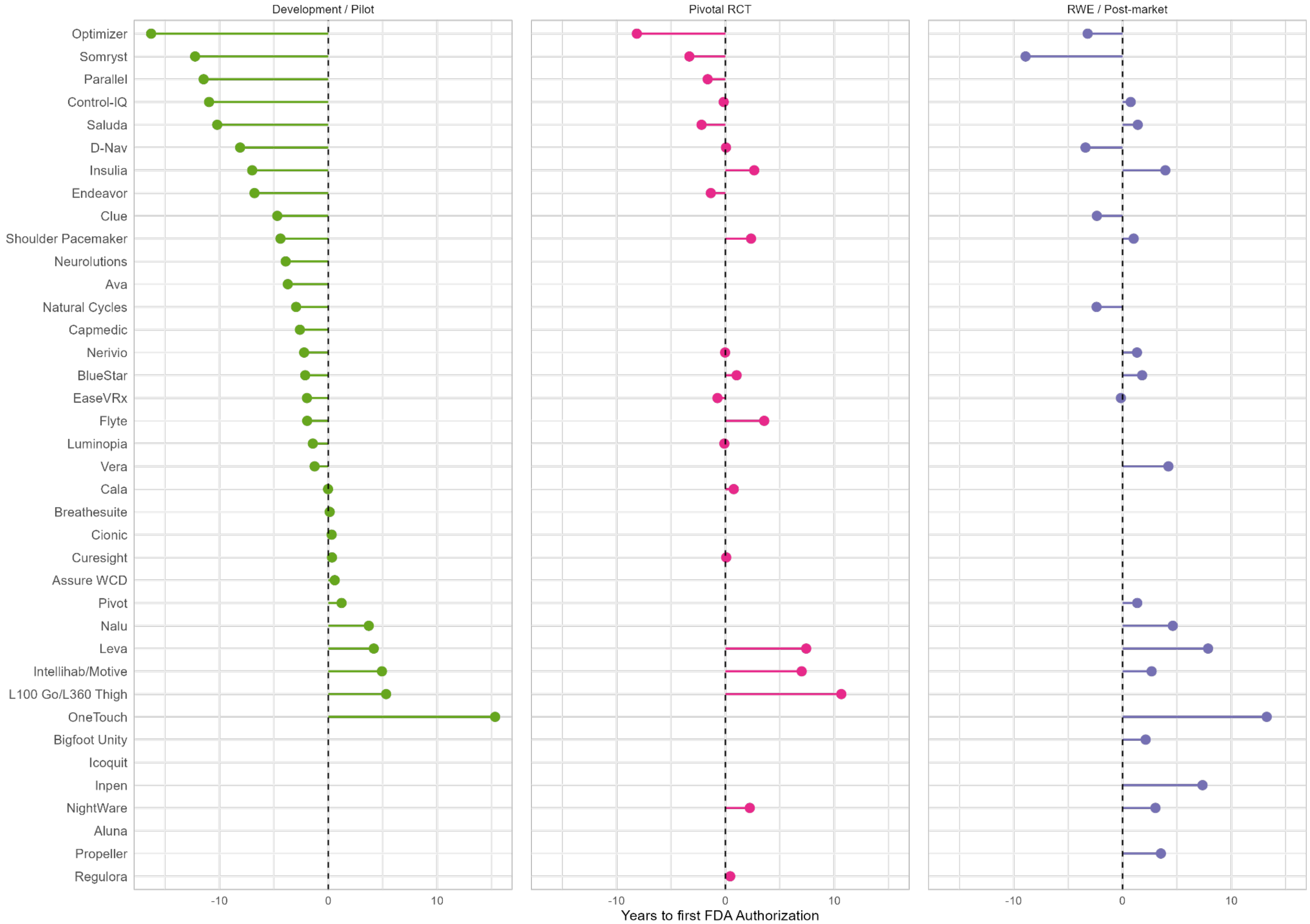

Legend: The earliest publication of results (in a published article or in a clinical trial register) for each DTx within each defined category are illustrated, in relation to the first FDA-authorization (set as time 0), for studies related to the indication for use in the first FDA-authorization. These categories were characterized as, briefly:

*Development/pilot*: in initial target population/indication per first FDA-authorization, and focusing on DTx development and/or pilot studies in preparation for the pivotal trial
*Pivotal RCT*: in initial target population/indication per first FDA-authorization
*RWE/post-market*: in initial target population/indication per first FDA-authorization and using real-world data, focusing on evaluation of DTx in practice, and/or following commercial availability

DTx with no studies/publications (n=5 DTx) or only DTx indication expansion/modification studies (n=2 DTx) and studies not related to the first indication for use (n=145 DTx indication expansion/modification studies) or with unclear classification (n=11) were not included in figure. For earliest publications/results of DTx, those related to development/pilot studies tended to be available prior to first authorization (n=21 DTx; median 3.9, range 0.0-16.3 years prior), and most RWE/post-market (n=16 DTx; median 2.8, range 0.7-13.2 years after) publications followed first FDA authorization. For pivotal RCTs, first publication was balanced (n=9 DTx; median 1.3, range 0.0-8.1 years prior; n=12 DTx; median 2.3, range 0.1-10.6 years after). Nonetheless, across all categories, considerable heterogeneity in the length of time from publication to/from FDA-authorization was observed.

Abbreviations: digital therapeutic (DTx); United States Food & Drug Administration (FDA); randomized controlled trial (RCT); real world evidence (RWE)

**Figure S6: Time from first study start to FDA-authorization among studies related to the first authorized indication, by study classification, stratified by FDA-authorization pathway (510(k) versus PMA and De Novo)**

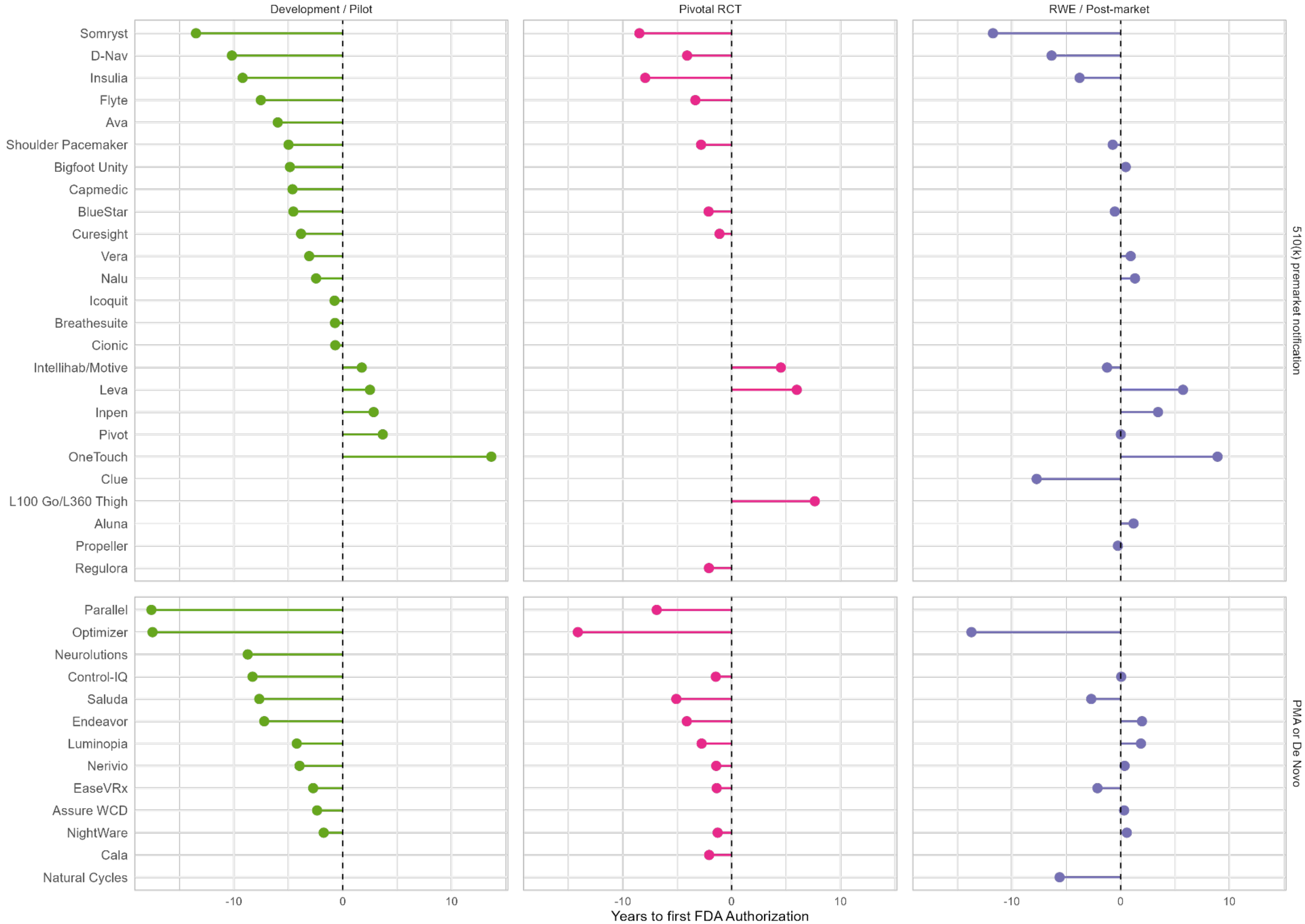

Legend: The start of the study period for evaluations of each DTx, stratified by first FDA-authorization pathway (510(k) premarket notification pathway or PMA+De Novo) within each defined category (e.g., development/pilot study period starting with the earliest development/pilot start), are illustrated, in relation to the first FDA-authorization (set as time 0), for studies related to the indication for use in the first FDA-authorization. These categories were characterized as, briefly:

*Development/pilot*: in initial target population/indication per first FDA-authorization, and focusing on DTx development and/or pilot studies in preparation for the pivotal trial
*Pivotal RCT*: in initial target population/indication per first FDA-authorization
*RWE/post-market*: in initial target population/indication per first FDA-authorization and using real-world data, focusing on evaluation of DTx in practice, and/or following commercial availability

DTx with no studies/publications (n=5 DTx) or only DTx indication expansion/modification studies (n=2 DTx), all authorized through the 510(k) premarket notification pathway, and studies not related to the first indication for use (n=145 DTx indication expansion/modification studies) or with unclear classification (n=11) were not included in figure. More DTx first authorized through the 510(k) pathway (21/32 DTx; 65.6%) did not have a pivotal trial than those first authorized through the PMA or De Novo pathway (3/13 DTx; 23.1%).

Development/pilot studies (n=11 DTx; median 7.2, range 1.7-17.6 years prior) and pivotal RCTs (n=10 DTx; median 2.4, range 1.3-14.1 years prior) for DTx authorized through the PMA or De Novo pathways were all initiated prior to authorization, though some DTx did not have studies in one/both of these categories. For RWE/post-market studies of DTx authorized through the PMA or De Novo pathways, timing of study for those starting after FDA-authorization was relatively short (n=6 DTx; median 0.5, range 0.1-2.0 years after). For DTx authorized through the 510(k) premarket notification pathway, timing of the start of studies was generally in line with the overall results.

Abbreviations: digital therapeutic (DTx); United States Food & Drug Administration (FDA); Pre-market Approval (PMA); randomized controlled trial (RCT); real world evidence (RWE)

**Figure S7: Time from last study completion to FDA-authorization among studies related to the first authorized indication, by study classification, stratified by FDA-authorization pathway (510(k) versus PMA and De Novo)**

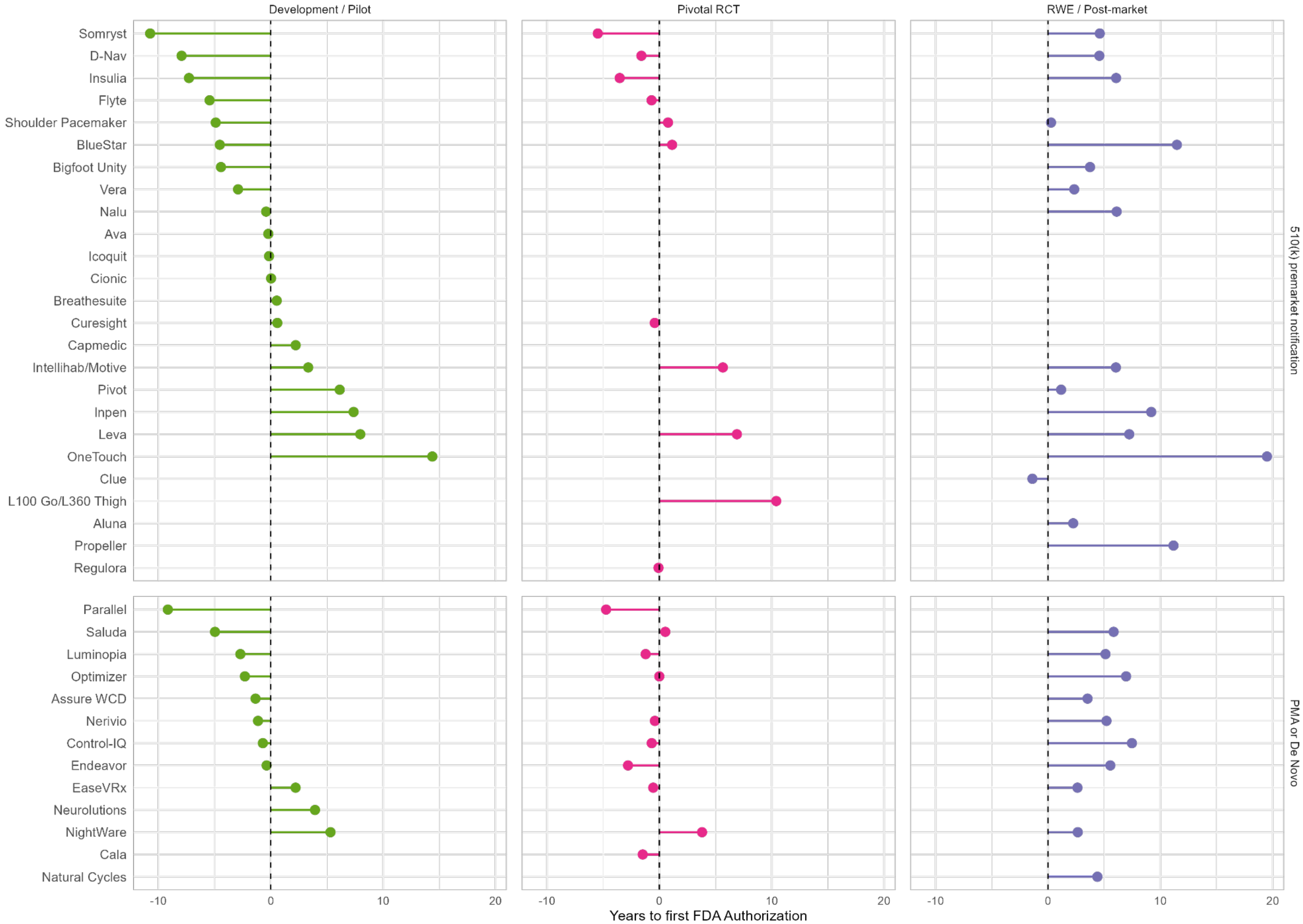

Legend: The end of the study period for evaluations of each DTx, stratified by first FDA-authorization pathway (510(k) premarket notification pathway or PMA+De Novo) within each defined category (e.g., development/pilot study period ending with the latest development/pilot completion) are illustrated, in relation to the first FDA-authorization (set as time 0), for studies related to the indication for use in the first FDA-authorization. These categories were characterized as, briefly:

*Development/pilot*: in initial target population/indication per first FDA-authorization, and focusing on DTx development and/or pilot studies in preparation for the pivotal trial
*Pivotal RCT*: in initial target population/indication per first FDA-authorization
*RWE/post-market*: in initial target population/indication per first FDA-authorization and using real-world data, focusing on evaluation of DTx in practice, and/or following commercial availability

DTx with no studies/publications (n=5 DTx) or only DTx indication expansion/modification studies (n=2 DTx), all authorized through the 510(k) premarket notification pathway, and studies not related to the first indication for use (n=145 DTx indication expansion/modification studies) or with unclear classification (n=11) were not included in figure. More DTx first authorized through the 510(k) pathway (21/32 DTx; 65.6%) did not have a pivotal trial than those first authorized through the PMA or De Novo pathway (3/13 DTx; 23.1%).

For all categories, clear differences in timing of study completion by first FDA-authorization pathway were not evident.

Abbreviations: digital therapeutic (DTx); United States Food & Drug Administration (FDA); Pre-market Approval (PMA); randomized controlled trial (RCT); real world evidence (RWE)

**Figure S8: Time from first publication/availability of results to FDA-authorization among studies related to the first authorized indication, by study classification, stratified by FDA-authorization pathway (510(k) versus PMA and De Novo)**

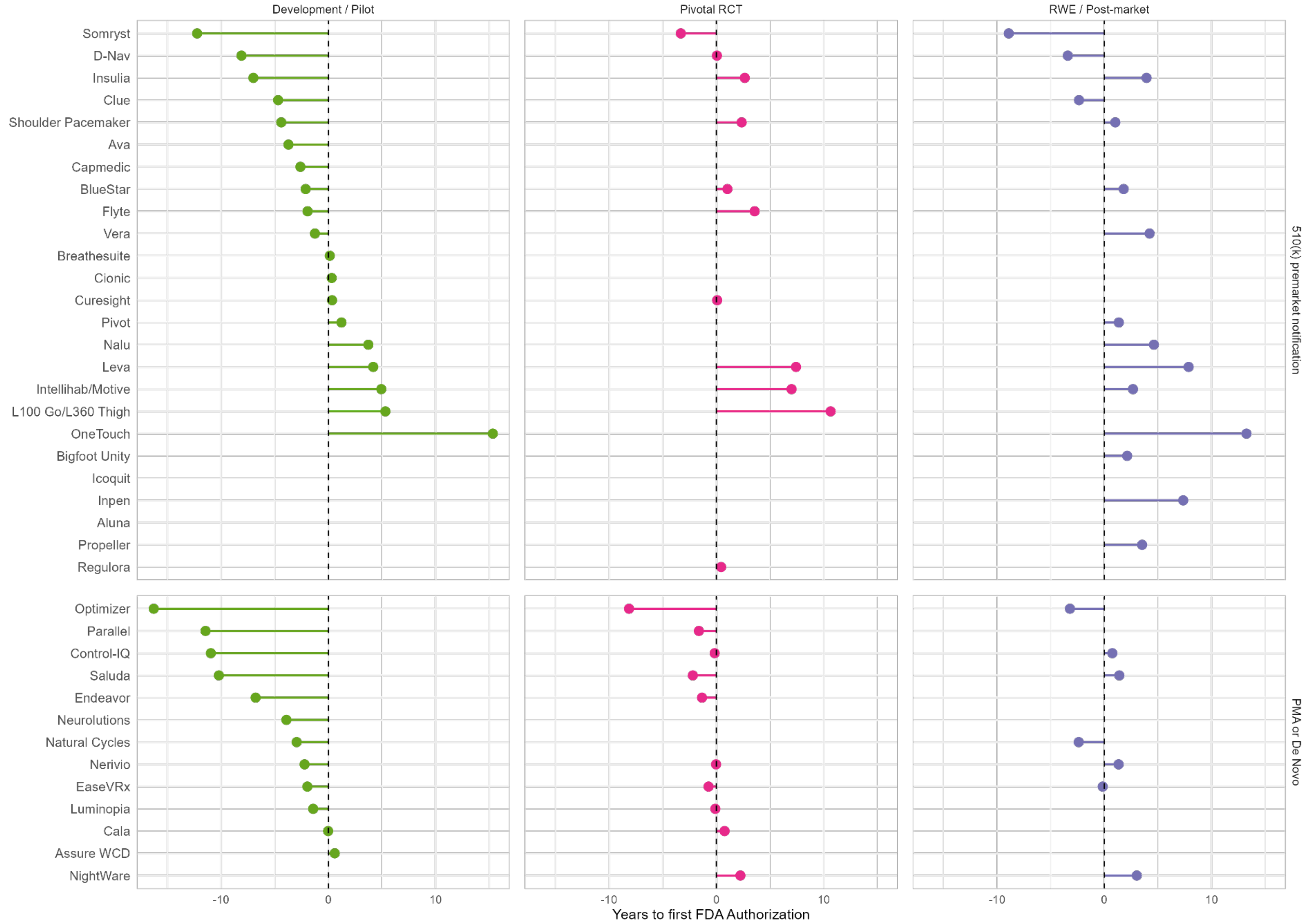

Legend: The earliest publication of results (in a published article or clinical trial register) for each DTx stratified by first FDA-authorization pathway (510(k) premarket notification pathway or PMA+De Novo) within each defined category are illustrated, in relation to the first FDA-authorization (set as time 0), for studies related to the indication for use in the first FDA-authorization. These categories were characterized as, briefly:

*Development/pilot*: in initial target population/indication per first FDA-authorization, and focusing on DTx development and/or pilot studies in preparation for the pivotal trial
*Pivotal RCT*: in initial target population/indication per first FDA-authorization
*RWE/post-market*: in initial target population/indication per first FDA-authorization and using real-world data, focusing on evaluation of DTx in practice, and/or following commercial availability

DTx with no studies/publications (n=5 DTx) or only DTx indication expansion/modification studies (n=2 DTx), all authorized through the 510(k) premarket notification pathway, and studies not related to the first indication for use (n=145 DTx indication expansion/modification studies) or with unclear classification (n=11) were not included in figure. More DTx first authorized through the 510(k) pathway (21/32 DTx; 65.6%) did not have a pivotal trial than those first authorized through the PMA or De Novo pathway (3/13 DTx; 23.1%).

Publications/results related to development/pilot studies (n=11 DTx; median 3.9, range 0.0-16.3 years prior) and pivotal RCTs (n=8 DTx; median 1.0, range 0.0-8.1 years prior) for DTx authorized through the PMA or De Novo pathways were generally available prior to first authorization, though for some DTx none were located for one of the categories; earliest publication of results for RWE-post-market studies of these DTx occurred within 5 years before (n=3 DTx; median 2.4, range 0.2-3.2 years prior) or after (n=4 DTx; median 1.4, range 0.7-3.0 years after) authorization, though for some DTx none were located for one of the categories. Clear trends specifically related to the timing of study publication/results availability for DTx authorized through the 510(k) premarket notification pathway were not evident, but were generally in line with the overall results.

Abbreviations: digital therapeutic (DTx); United States Food & Drug Administration (FDA); Pre-market Approval (PMA); randomized controlled trial (RCT); real world evidence (RWE)

**Figure S9: Time from first study start to FDA-authorization among studies related to the first authorized indication, by study classification, stratified by DTx components**

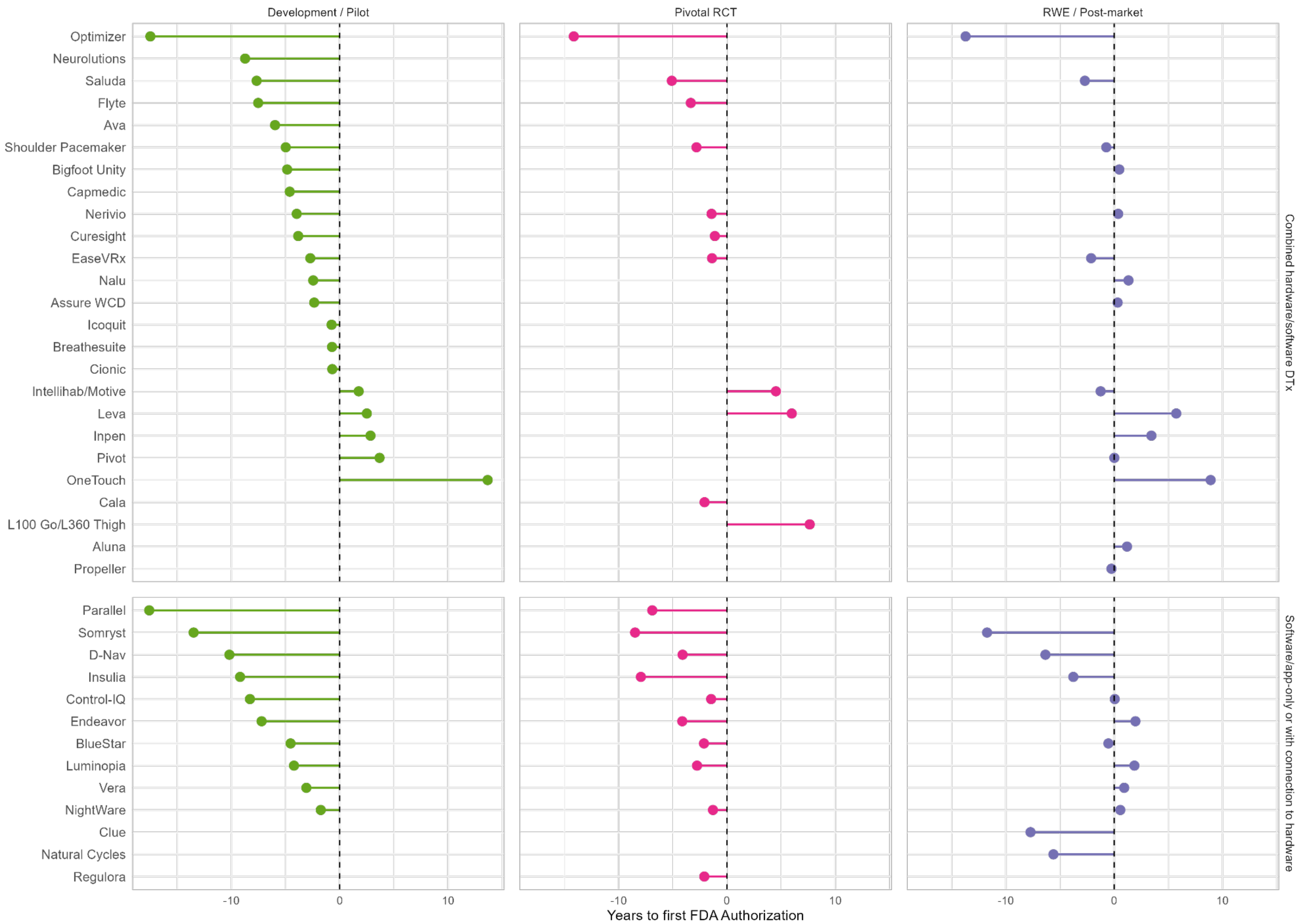

Legend: The start of study period for evaluations of each DTx, stratified by DTx components (combined hardware/software DTx; DTx which are software/app-only or with connection to hardware) within each category (e.g., development/pilot study period starting with the earliest development/pilot start), are illustrated, in relation to first FDA-authorization (set as time 0), for studies related to the indication for use in the first FDA-authorization. These categories were characterized as, briefly:

*Development/pilot*: in initial target population/indication per first FDA-authorization, and focusing on DTx development and/or pilot studies in preparation for the pivotal trial
*Pivotal RCT*: in initial target population/indication per first FDA-authorization
*RWE/post-market*: in initial target population/indication per first FDA-authorization and using real-world data, focusing on evaluation of DTx in practice, and/or following commercial availability

DTx with no studies/publications (n=5 DTx) or only DTx indication expansion/modification studies (n=2 DTx), all combined hardware/software DTx, and studies not related to the first indication for use (n=145 DTx indication expansion/modification studies) or with unclear classification (n=11) were not included in figure. More combined hardware/software DTx (21/32 DTx; 65.6%) did not have a pivotal trial than those which are mobile applications or software devices which are standalone or connect to hardware components not part of the DTx (3/13 DTx; 23.1%).

Development/pilot studies (n=10 DTx; median 7.7, range 1.7-17.6 years prior) and pivotal RCTs (n=10 DTx; median 3.4, range 1.3-8.5 years prior) for DTx which are software/app-only or with connection to hardware were all initiated prior to authorization, though some DTx did not have studies in one/both of these categories; for these DTx, a trend was noted for RWE/post-market studies to be initiated farther in advance (n=6 DTx; median 6.0, range 0.5-11.7 years prior) or shortly after first FDA-authorization (n=5 DTx; median 0.9, range 0.1-2.0 years after). For combined hardware/software DTx, timing of study start was generally in line with overall results, though these DTx were less likely to have a pivotal RCT or RWE/post-market study than were DTx which are software/app-only or with connection to hardware.

Abbreviations: digital therapeutic (DTx); United States Food & Drug Administration (FDA); randomized controlled trial (RCT); real world evidence (RWE); mobile application (app)

**Figure S10: Time from last study completion to FDA-authorization among studies related to the first authorized indication, by study classification, stratified by DTx components**

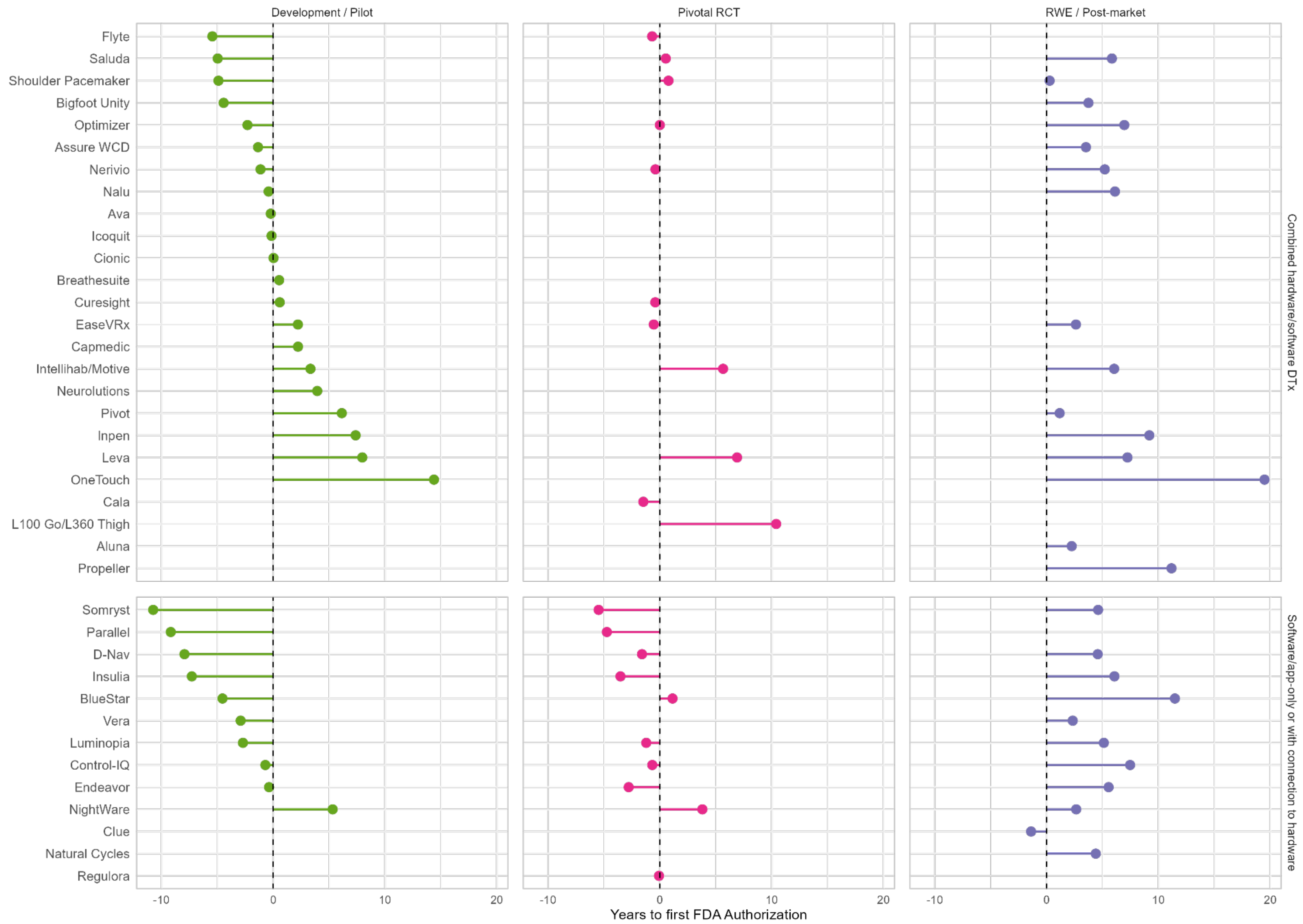

Legend: The end of the study period for evaluations of each DTx within each defined category (e.g., development/pilot study period ending with the latest development/pilot completion) are illustrated, in relation to the first FDA-authorization (set as time 0), for studies related to the indication for use in the first FDA-authorization. These categories were characterized as, briefly:

*Development/pilot*: in initial target population/indication per first FDA-authorization, and focusing on DTx development and/or pilot studies in preparation for the pivotal trial
*Pivotal RCT*: in initial target population/indication per first FDA-authorization
*RWE/post-market*: in initial target population/indication per first FDA-authorization and using real-world data, focusing on evaluation of DTx in practice, and/or following commercial availability

DTx with no studies/publications (n=5 DTx) or only DTx indication expansion/modification studies (n=2 DTx), all combined hardware/software DTx, and studies not related to the first indication for use (n=145 DTx indication expansion/modification studies) or with unclear classification (n=11) were not included in figure. More combined hardware/software DTx (21/32 DTx; 65.6%) did not have a pivotal trial than those which are software/app-only or with connection to hardware (3/13 DTx; 23.1%).

Development/pilot studies and pivotal RCTs for DTx which are software/app-only or with connection to hardware were shifted left (i.e., occurred relatively earlier in relation to first FDA-authorization) in comparison to combined hardware/software DTx. Regardless of DTx components, differences in the timing of the completion of RWE/post-market study periods were not evident, though DTx which are software/app-only or with connection to hardware more commonly had this type of study.

Abbreviations: digital therapeutic (DTx); United States Food & Drug Administration (FDA); randomized controlled trial (RCT); real world evidence (RWE); mobile application (app)

**Figure S11: Time from first publication/availability of results to FDA-authorization among studies related to the first authorized indication, by study classification, stratified by DTx components**

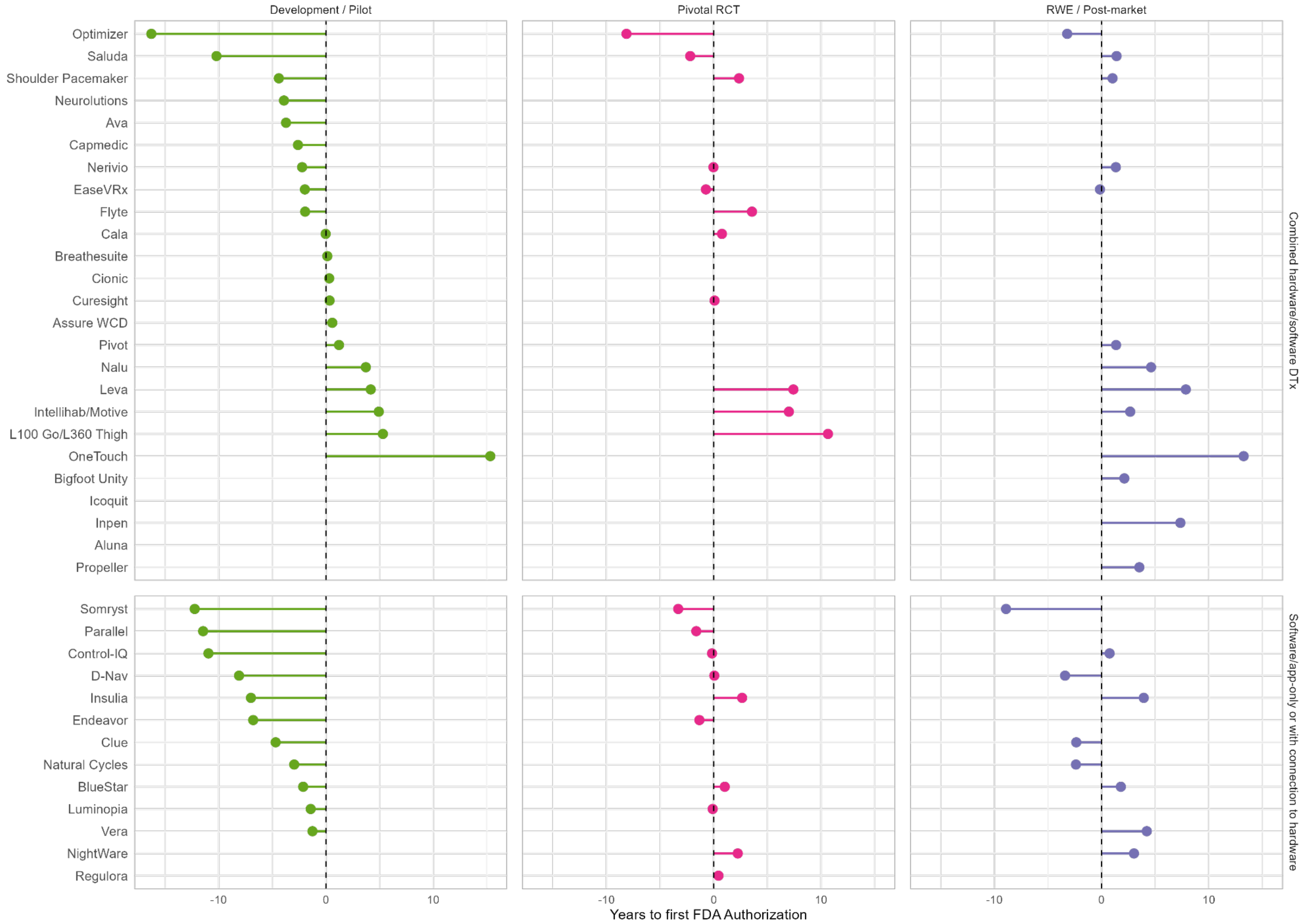

Legend: The earliest publication of results (in a published article or in a clinical trial register) for each DTx within each defined category are illustrated, in relation to the first FDA-authorization (set as time 0), for studies related to the indication for use in the first FDA-authorization. These categories were characterized as, briefly:

*Development/pilot*: in initial target population/indication per first FDA-authorization, and focusing on DTx development and/or pilot studies in preparation for the pivotal trial
*Pivotal RCT*: in initial target population/indication per first FDA-authorization
*RWE/post-market*: in initial target population/indication per first FDA-authorization and using real-world data, focusing on evaluation of DTx in practice, and/or following commercial availability

DTx with no studies/publications (n=5 DTx) or only DTx indication expansion/modification studies (n=2 DTx), all combined hardware/software DTx, and studies not related to the first indication for use (n=145 DTx indication expansion/modification studies) or with unclear classification (n=11) were not included in figure. More combined hardware/software DTx (21/32 DTx; 65.6%) did not have a pivotal trial than those which are software/app-only or with connection to hardware (3/13 DTx; 23.1%).

Development/pilot studies (n=11 DTx; median 6.8, range 1.3-12.2 years prior) of DTx which are software/app-only or with connection to hardware were published/had results available prior to authorization, though some DTx did not have studies in this category; for these DTx, a slight trend was noted for more of these studies having more pivotal RCTs and RWE/post-market studies with results published/available prior to FDA-authorization and for this to occur slightly earlier (relative to FDA-authorization) than for combined hardware/software DTx.

Abbreviations: digital therapeutic (DTx); United States Food & Drug Administration (FDA); randomized controlled trial (RCT); real world evidence (RWE); mobile application (app)

**Figure S12: Time from first study start to FDA-authorization among studies related to the first authorized indication, by study classification, stratified by timing of first authorization (2002-2019 or 2020-2022)**

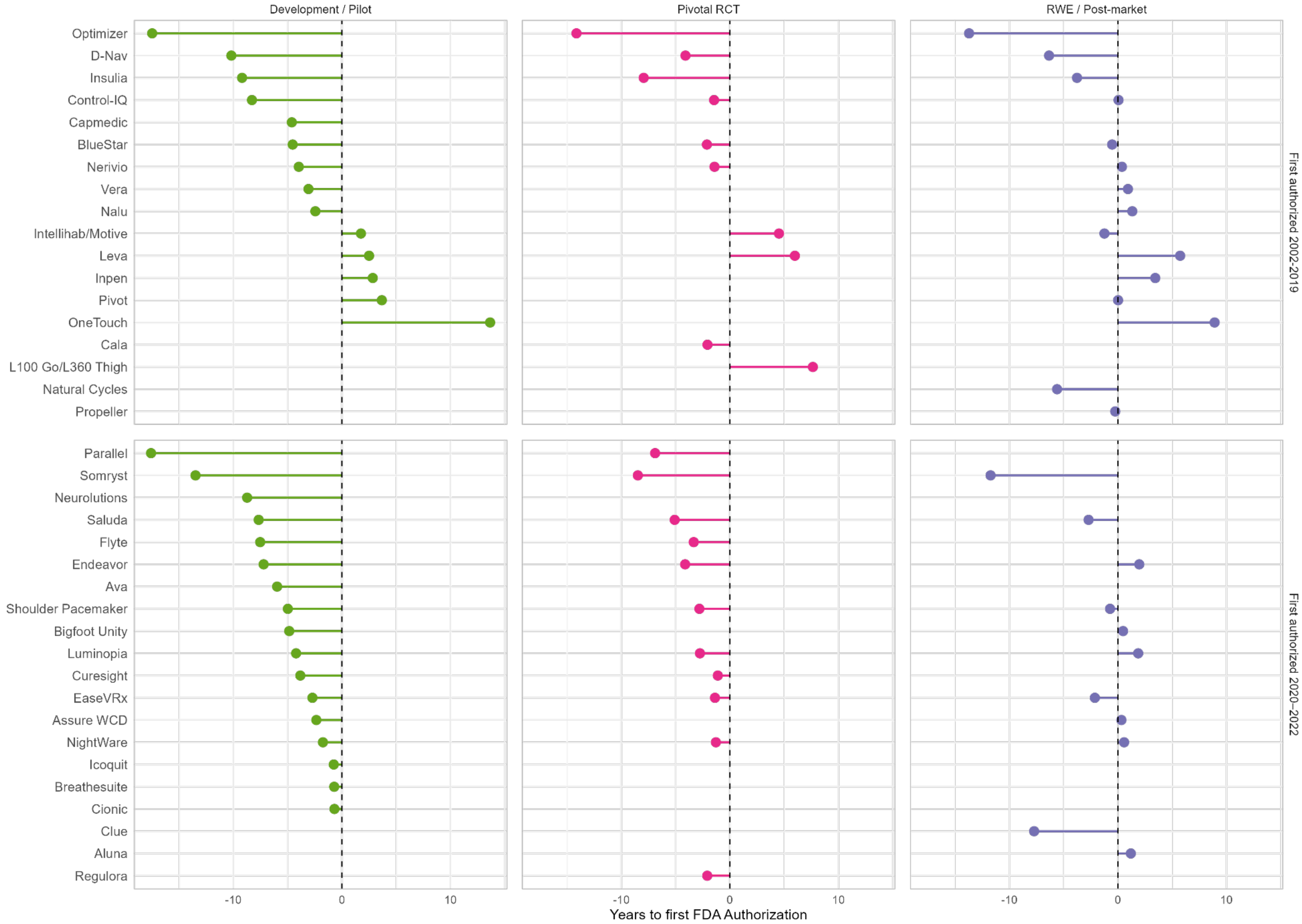

Legend: The start of the study period for evaluations of each DTx, stratified by year of first FDA-authorization (2002-2019; 2020-2022) within each category (e.g., development/pilot study period starting with the earliest development/pilot start), are illustrated, in relation to the first FDA-authorization (set as time 0), for studies related to the indication for use in the first FDA-authorization. These categories were characterized as, briefly:

*Development/pilot*: in initial target population/indication per first FDA-authorization, and focusing on DTx development and/or pilot studies in preparation for the pivotal trial
*Pivotal RCT*: in initial target population/indication per first FDA-authorization
*RWE/post-market*: in initial target population/indication per first FDA-authorization and using real-world data, focusing on evaluation of DTx in practice, and/or following commercial availability

DTx with no studies/publications (n=5 DTx) or only DTx indication expansion/modification studies (n=2 DTx) and studies not related to first indication for use (n=145 DTx indication expansion/modification studies) or with unclear classification (n=11) were not included in figure. No difference was found for the availability of pivotal trials for DTx which received first FDA-authorization 2020-2022 (13/24 DTx no pivotal trial; 54.2%) versus DTx which received first FDA-authorization 2002-2019 (11/21 DTx no pivotal trial; 52.4%).

Development/pilot studies (n=17 DTx; median 4.9, range 0.7-17.6 years prior) and pivotal RCTs (n=11 DTx; median 2.8, range 1.1-8.5 years prior) for DTx which received first FDA-authorization 2020-2022 were all initiated prior to authorization, though some DTx did not have studies in one of these categories. For RWE/post-market studies of DTx which received first FDA-authorization 2020-2022, fewer DTx had studies in this category (11/24 DTx; 45.8%; versus 15/21 DTx which received first FDA-authorization 2002-2019; 71.4%) and the timing of study start after FDA-authorization is relatively short (n=6 DTx; median 0.9, range 0.3-2.0 years after), as an artifact of the fact that study searches were conducted in the summer of 2024, allowing a short amount of time in which these studies could have been initiated. For DTx which received first FDA-authorization 2002-2019, timing of the start of studies was generally in line with the overall results.

Abbreviations: digital therapeutic (DTx); United States Food & Drug Administration (FDA); randomized controlled trial (RCT); real world evidence (RWE)

**Figure S13: Time from last study completion to FDA-authorization among studies related to the first authorized indication, by study classification, stratified by timing of first authorization (2002-2019 or 2020-2022)**

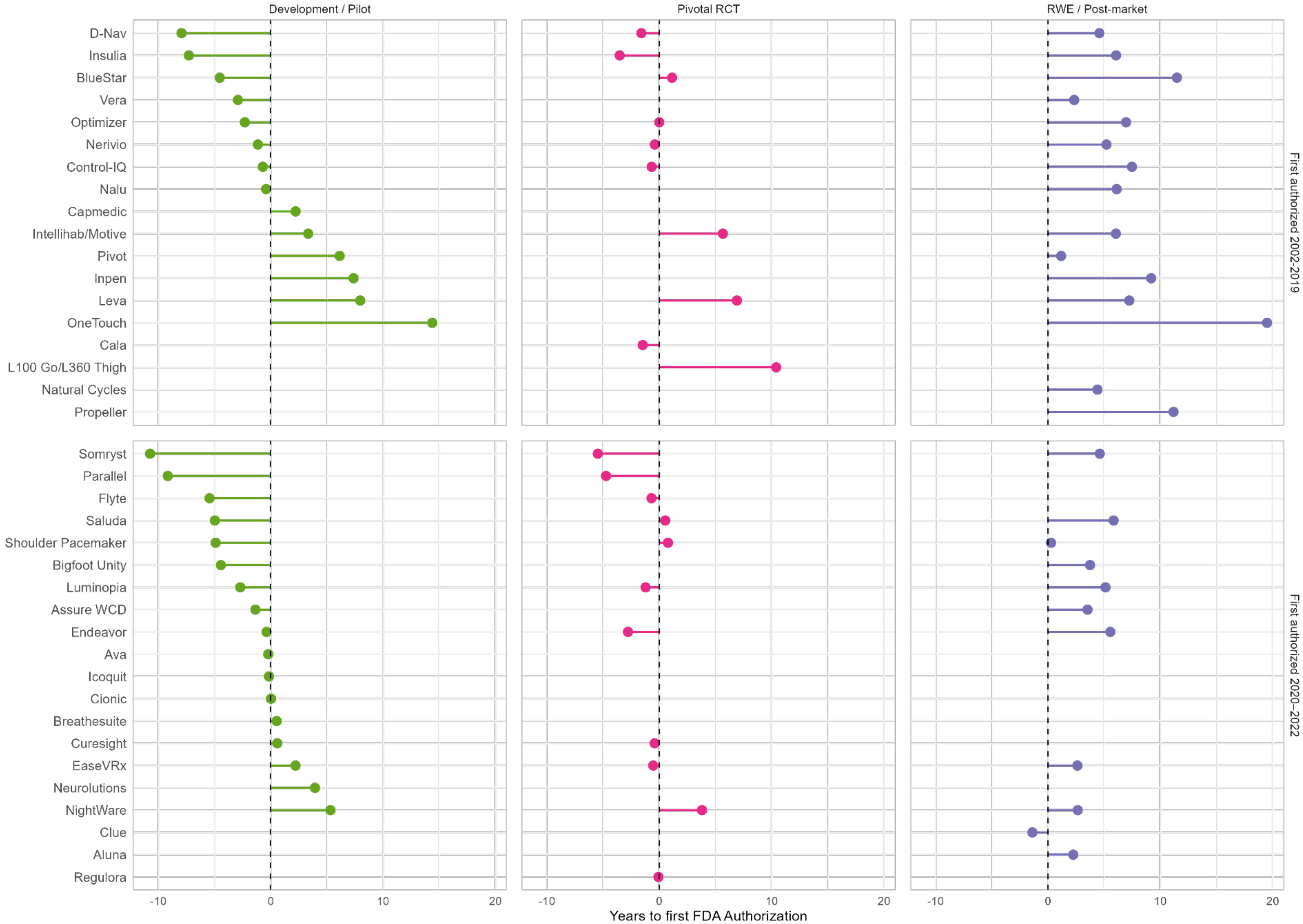

Legend: The end of the study period for evaluations of each DTx, stratified by year of first FDA-authorization (2002-2019; 2020-2022) within each defined category (e.g., development/pilot study period ending with the latest development/pilot completion) are illustrated, in relation to the first FDA-authorization (set as time 0), for studies related to the indication for use in the first FDA-authorization. These categories were characterized as, briefly:

*Development/pilot*: in initial target population/indication per first FDA-authorization, and focusing on DTx development and/or pilot studies in preparation for the pivotal trial
*Pivotal RCT*: in initial target population/indication per first FDA-authorization
*RWE/post-market*: in initial target population/indication per first FDA-authorization and using real-world data, focusing on evaluation of DTx in practice, and/or following commercial availability

DTx with no studies/publications (n=5 DTx) or only DTx indication expansion/modification studies (n=2 DTx) and studies not related to the first indication for use (n=145 DTx indication expansion/modification studies) or with unclear classification (n=11) were not included in figure. No difference was found for the availability of pivotal trials for DTx which received first FDA-authorization 2020-2022 (13/24 DTx no pivotal trial; 54.2%) versus DTx which received first FDA-authorization 2002-2019 (11/21 DTx no pivotal trial; 52.4%).

Regardless of when DTx received first FDA-authorization (2002-2019 or 2020-2022), timing of the completion of RWE/post-market study periods virtually always occurred following first FDA-authorization, though time for those authorized earlier (2002-2019: n=15 DTx: median 6.1, range 1.2-19.5 years after; 2020-2022: n=10 DTx: median 3.6, range 0.3-5.8 after) was generally longer as an artifact of the longer time to the cut-off of study inclusion (summer 2024). For development/pilot studies and pivotal RCTs, clear differences in timing of study completion by year of first FDA-authorization were not evident, with heterogeneity observed within these classifications/strata.

Abbreviations: digital therapeutic (DTx); United States Food & Drug Administration (FDA); randomized controlled trial (RCT); real world evidence (RWE)

**Figure S14: Time from first publication/availability of results to FDA-authorization among studies related to the first authorized indication, by study classification, stratified by timing of first authorization (2002-2019 or 2020-2022)**

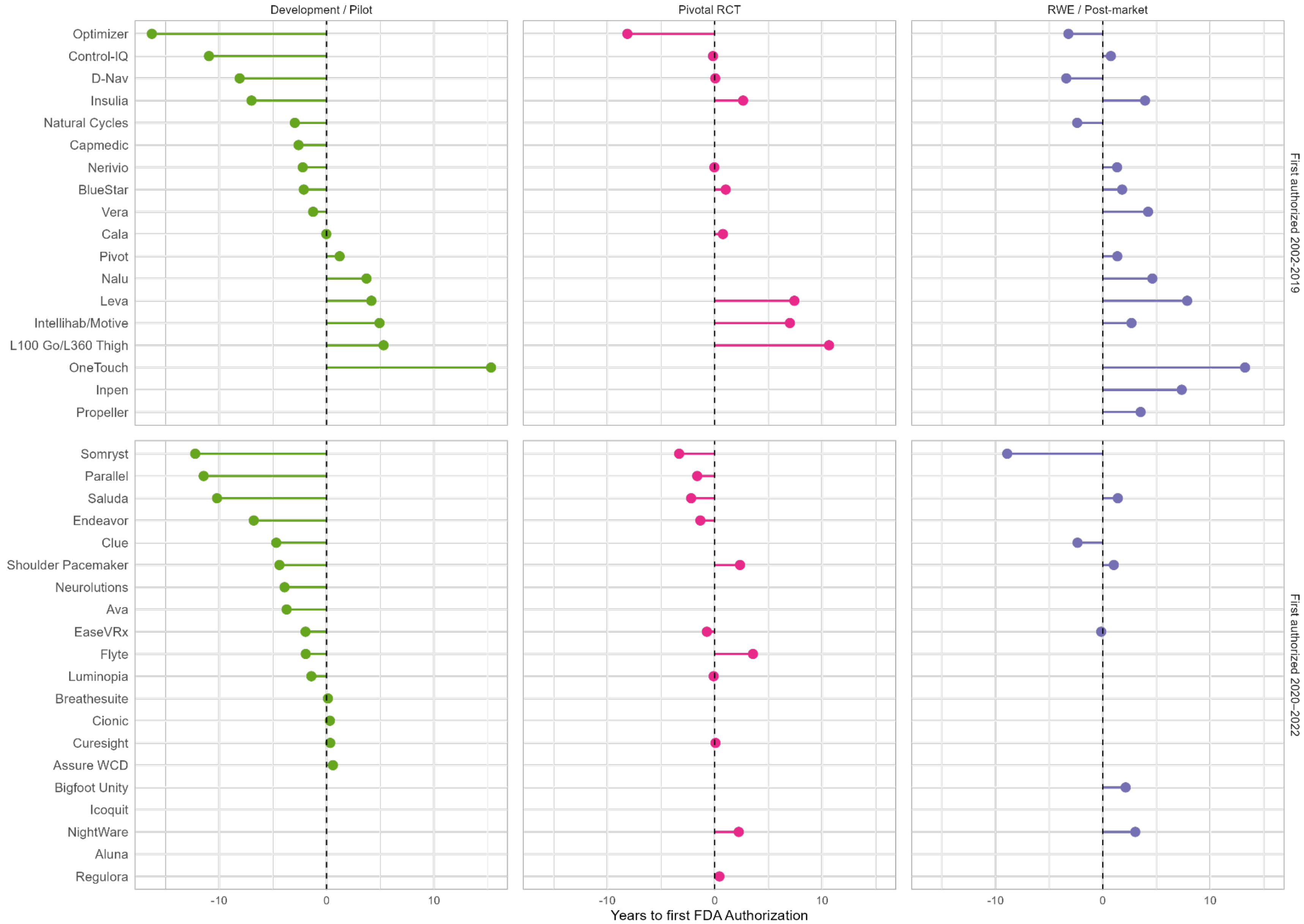

Legend: The earliest publication of results (in a published article or in a clinical trial register) for each DTx, stratified by year of first FDA-authorization (2002-2019; 2020-2022) within each defined category are illustrated, in relation to the first FDA-authorization (set as time 0), for studies related to the indication for use in the first FDA-authorization. These categories were characterized as, briefly:

*Development/pilot*: in initial target population/indication per first FDA-authorization, and focusing on DTx development and/or pilot studies in preparation for the pivotal trial
*Pivotal RCT*: in initial target population/indication per first FDA-authorization
*RWE/post-market*: in initial target population/indication per first FDA-authorization and using real-world data, focusing on evaluation of DTx in practice, and/or following commercial availability

DTx with no studies/publications (n=5 DTx) or only DTx indication expansion/modification studies (n=2 DTx) and studies not related to the first indication for use (n=145 DTx indication expansion/modification studies) or with unclear classification (n=11) were not included in figure. No difference was found for the availability of pivotal trials for DTx which received first FDA-authorization 2020-2022 (13/24 DTx no pivotal trial; 54.2%) versus DTx which received first FDA-authorization 2002-2019 (11/21 DTx no pivotal trial; 52.4%).

Publications/results related to development/pilot studies for DTx which received first FDA-authorization 2020-2022 were generally available prior to first authorization (n=11 DTx; median 4.4, range 1.4-12.2 years prior) or shortly thereafter (n=4 DTx; median 0.3, range 0.1, 0.6 years after), though for some DTx none were located. For RWE/post-market studies of DTx which received first FDA-authorization 2020-2022, fewer DTx had studies in this category (7/24 DTx; 29.2%), as an artifact of the fact that study searches were conducted in the summer of 2024, allowing a short amount of time in which these studies could have been completed and published/results made available. Clear trends specifically related to the timing of study publication/results availability for DTx receiving first FDA-authorization earlier (2002-2019) were not evident, but were generally in line with the overall results.

Abbreviations: digital therapeutic (DTx); United States Food & Drug Administration (FDA); randomized controlled trial (RCT); real world evidence (RWE)

**Document S1: Totality of evidence for each DTx**

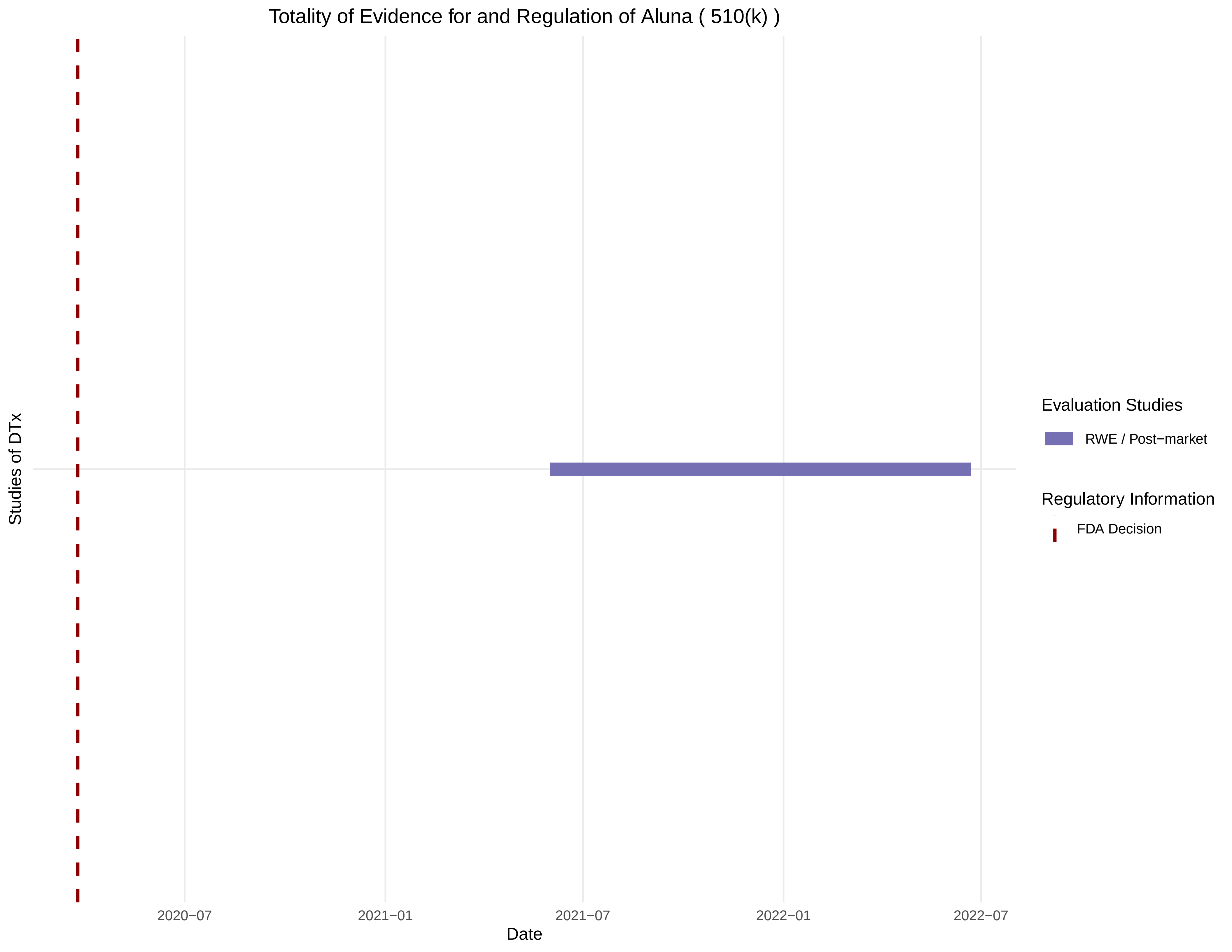
Totality of Evidence for and Regulation of Aluna ( 510(k) )
Studies of DTx
Date
2020−07
2021−01
2021−07
2022−01
2022−07
Evaluation Studies
RWE / Post−market
Regulatory Information
FDA Decision

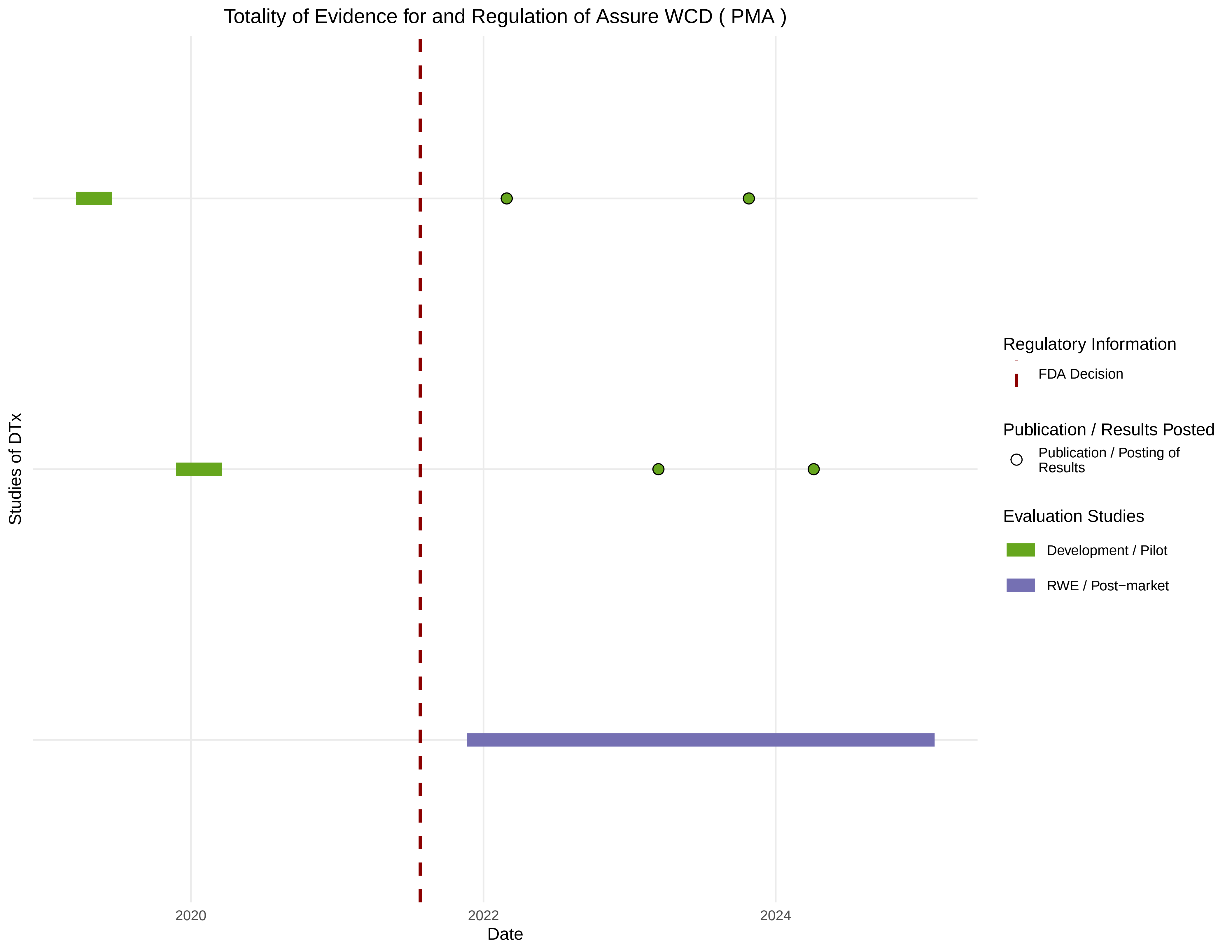

Totality of Evidence for and Regulation of Assure WCD ( PMA )
Studies of DTx
2020
2022
2024
Date
Regulatory Information
FDA Decision
Publication / Results Posted
Publication / Posting of Results
Evaluation Studies
Development / Pilot
RWE / Post−market

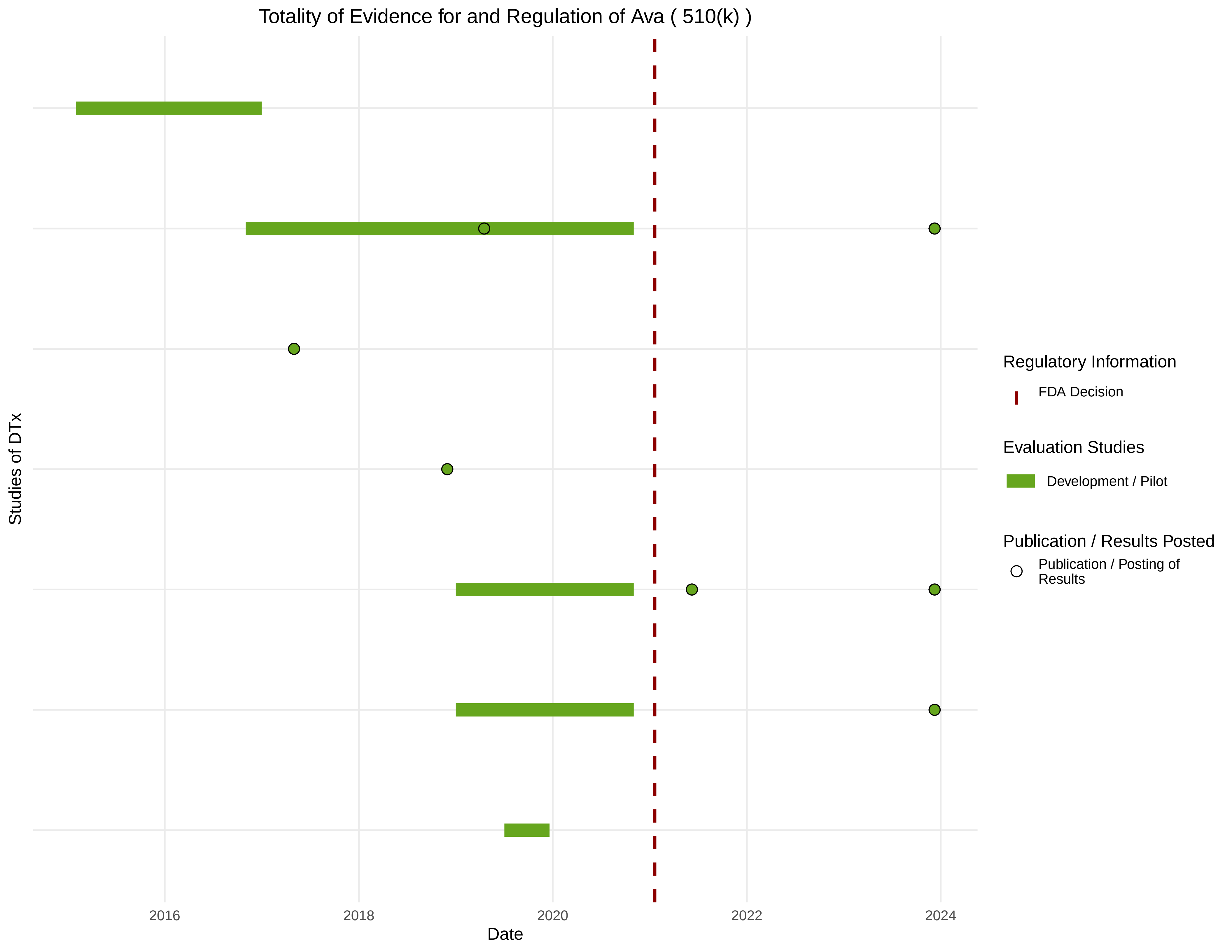
Totality of Evidence for and Regulation of Ava ( 510(k) )
Studies of DTx
2016
2018
2020
2022
2024
Date
Regulatory Information
FDA Decision
Evaluation Studies
Development / Pilot
Publication / Results Posted
Publication / Posting of Results

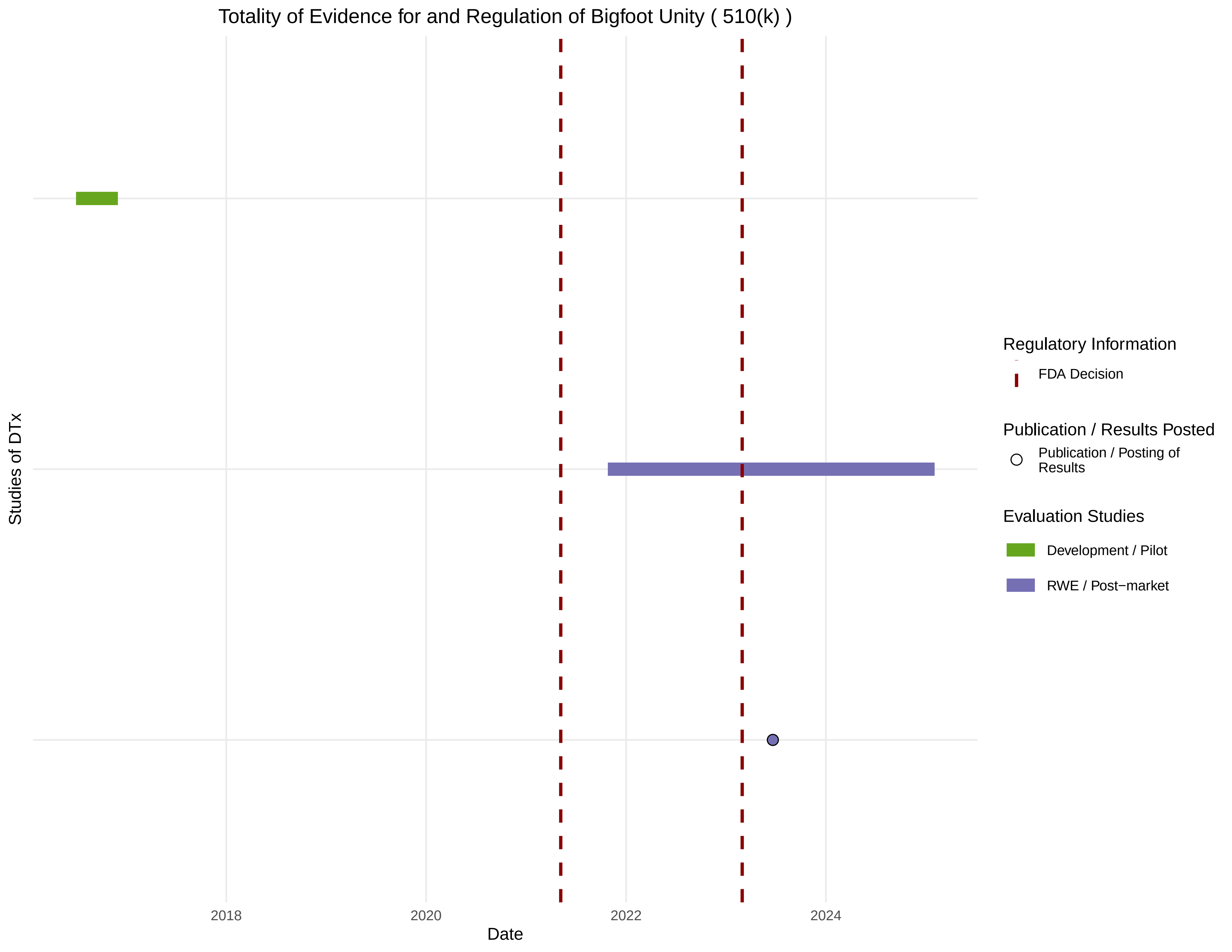
Totality of Evidence for and Regulation of Bigfoot Unity ( 510(k) )
Studies of DTx
Date
2018
2020
2022
2024
Regulatory Information
FDA Decision
Publication / Results Posted
Publication / Posting of Results
Evaluation Studies
Development / Pilot
RWE / Post−market

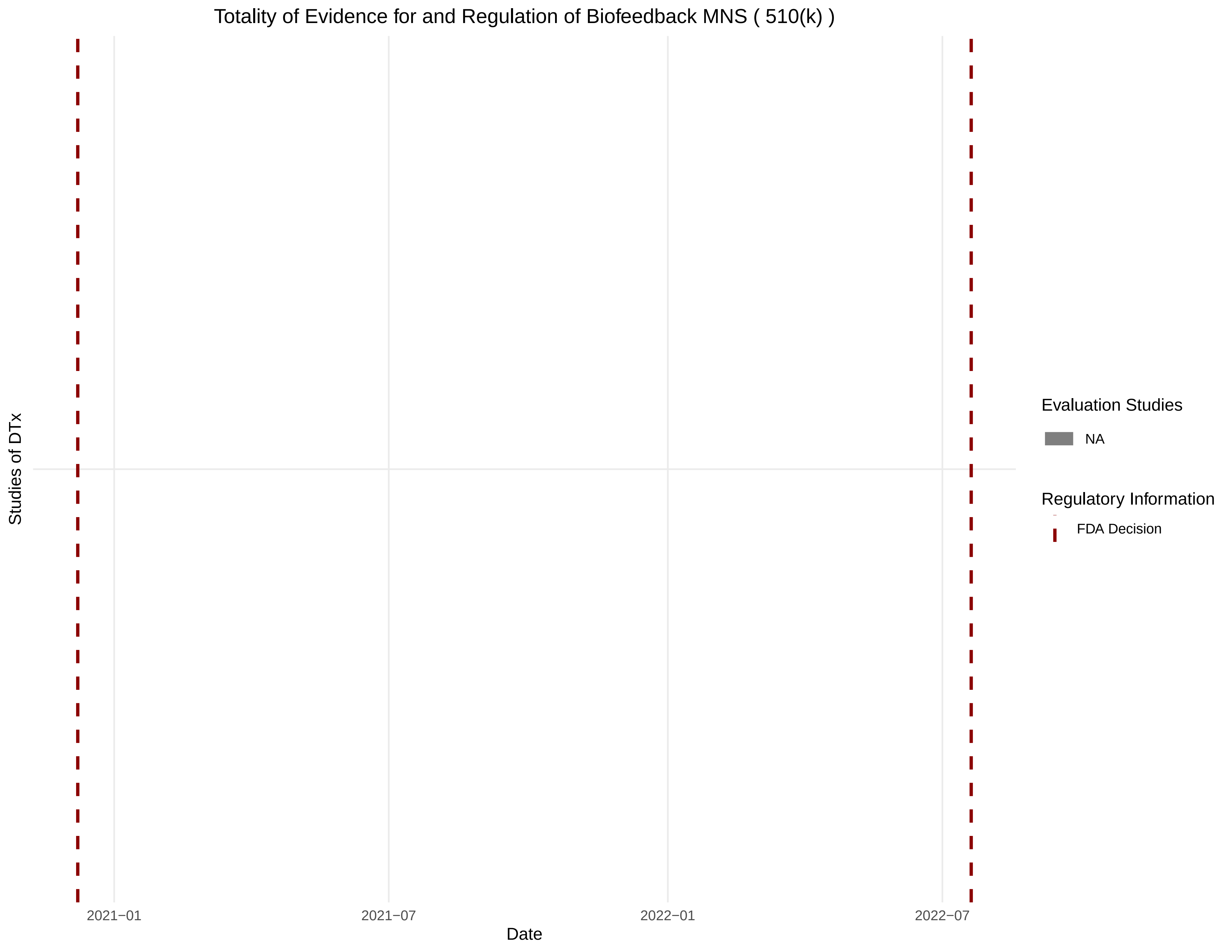
Totality of Evidence for and Regulation of Biofeedback MNS ( 510(k) )
Studies of DTx
Date
2021−01
2021−07
2022−01
2022−07
Evaluation Studies
NA
Regulatory Information
FDA Decision

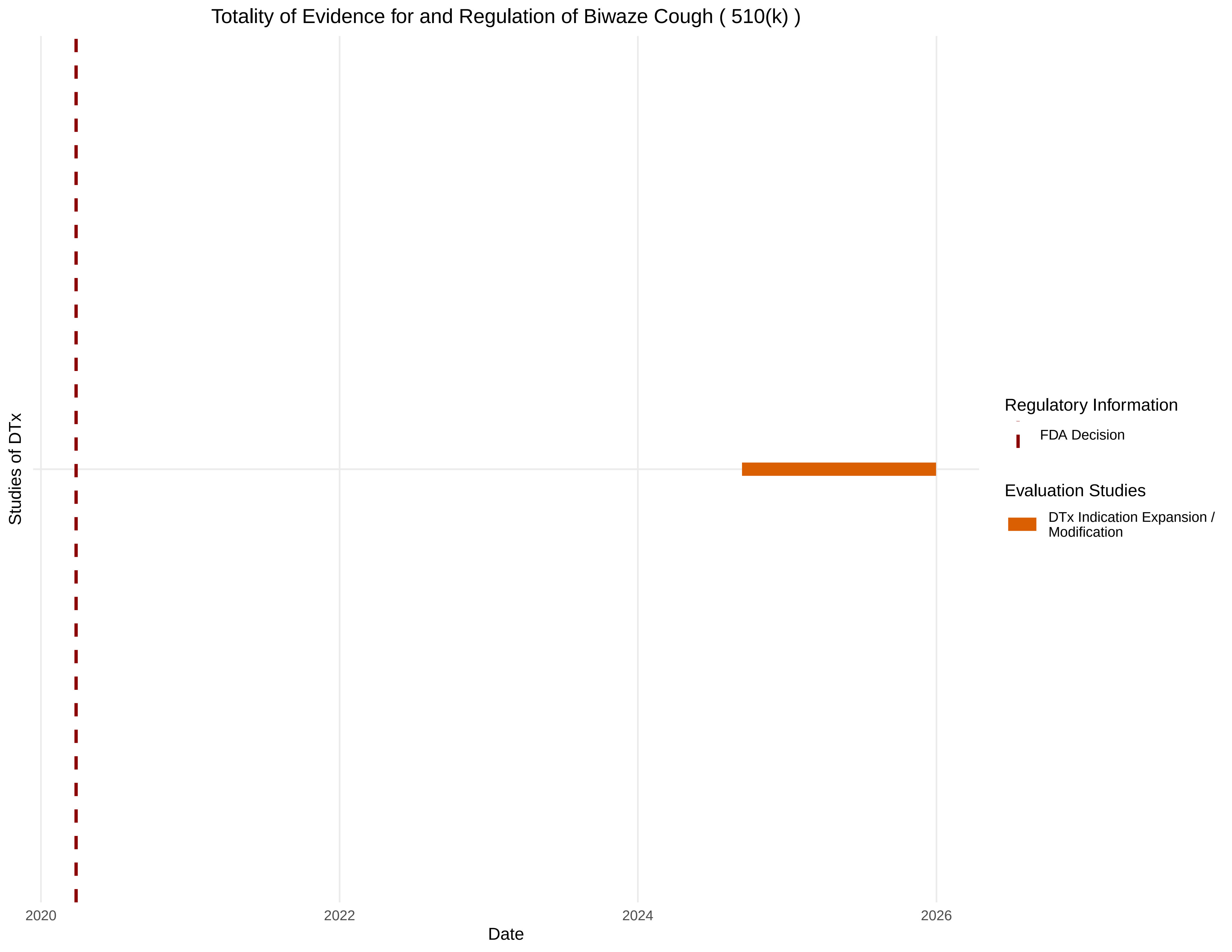

Totality of Evidence for and Regulation of Biwaze Cough ( 510(k) )
Studies of DTx
2020
2022
2024
2026
Date
Regulatory Information
FDA Decision
Evaluation Studies
DTx Indication Expansion / Modification

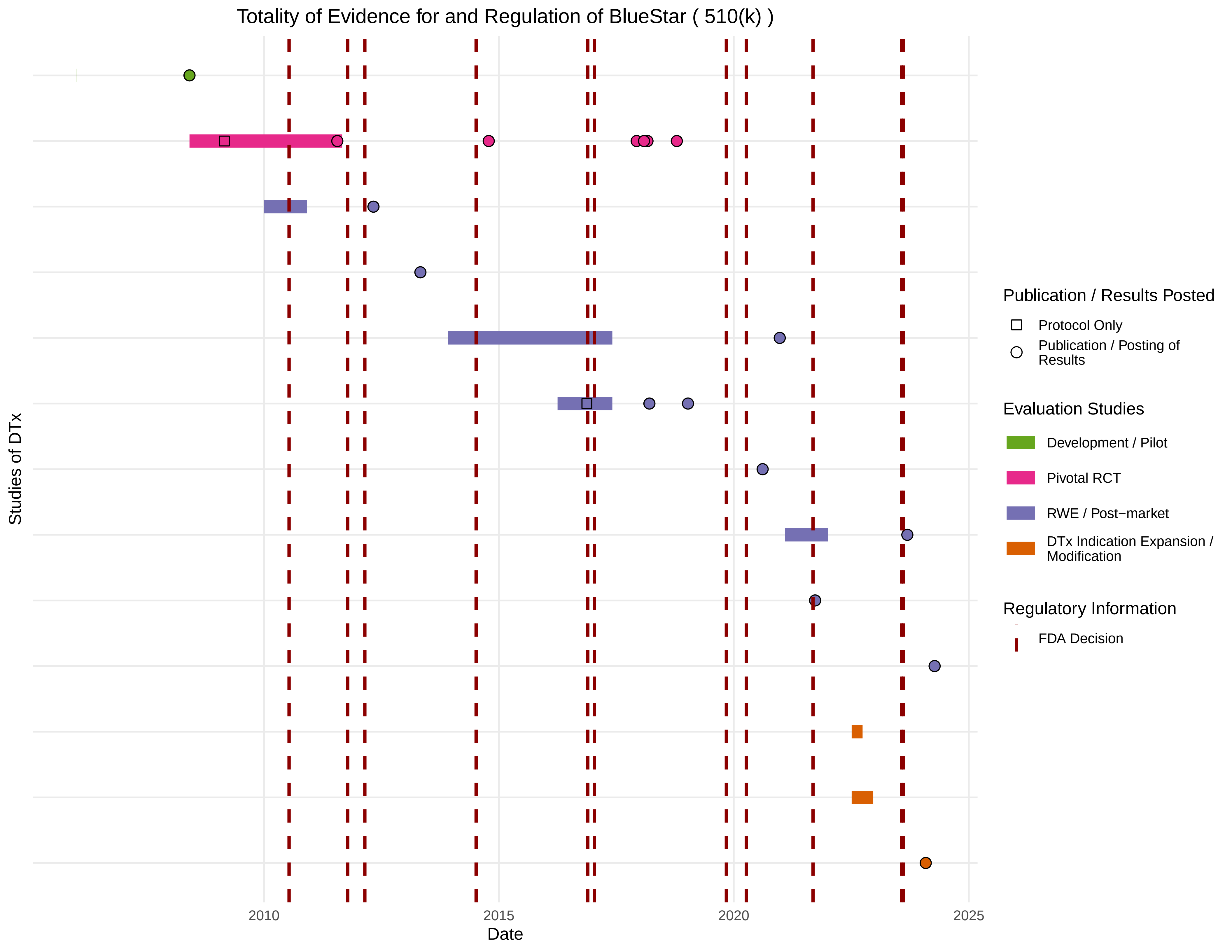

Totality of Evidence for and Regulation of BlueStar ( 510(k) )
Studies of DTx
Date
2010
2015
2020
2025
Publication / Results Posted
Protocol Only
Publication / Posting of Results
Evaluation Studies
Development / Pilot
Pivotal RCT
RWE / Post−market
DTx Indication Expansion / Modification
Regulatory Information
FDA Decision

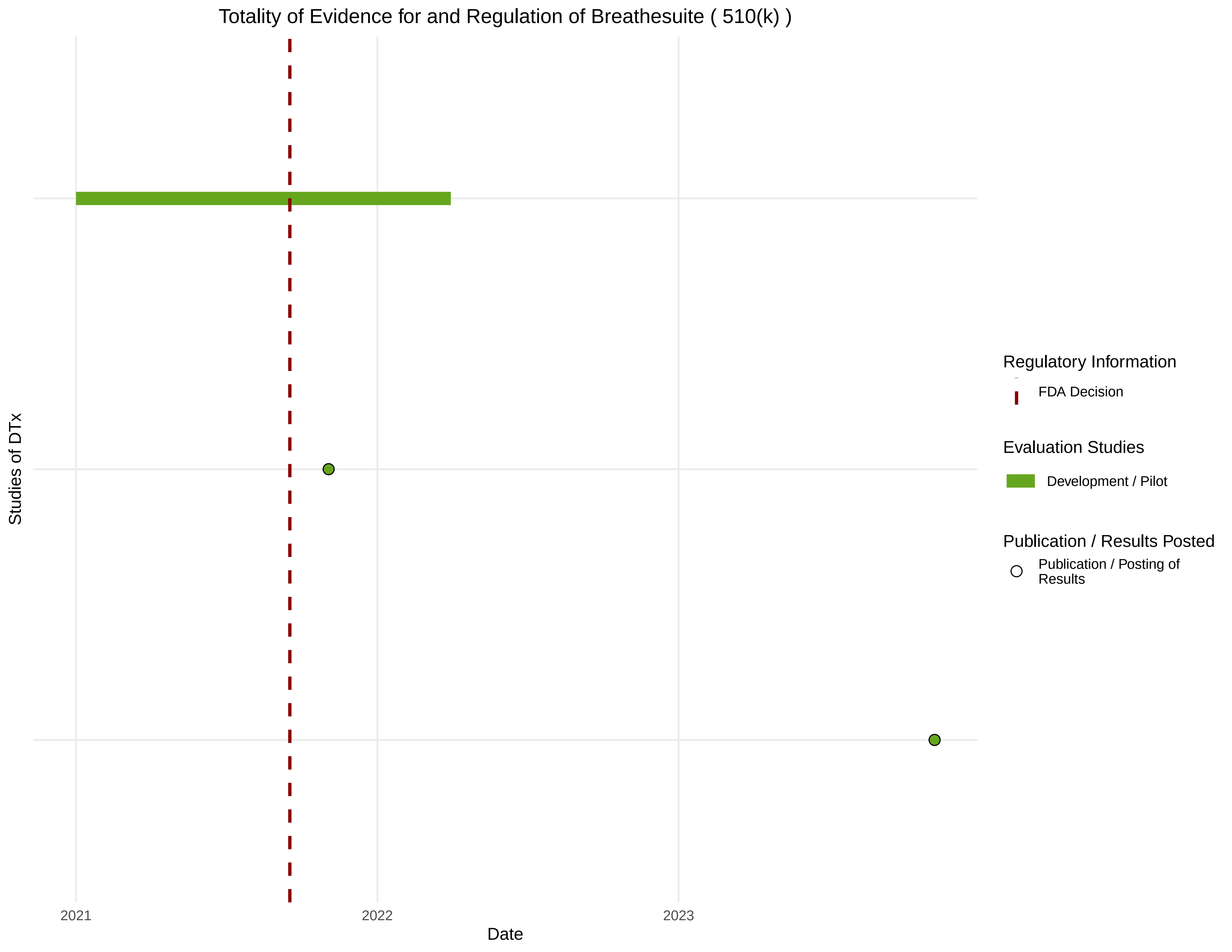
Totality of Evidence for and Regulation of Breathesuite ( 510(k) )
Studies of DTx
Date
2021
2022
2023
Regulatory Information
FDA Decision
Evaluation Studies
Development / Pilot
Publication / Results Posted
Publication / Posting of Results

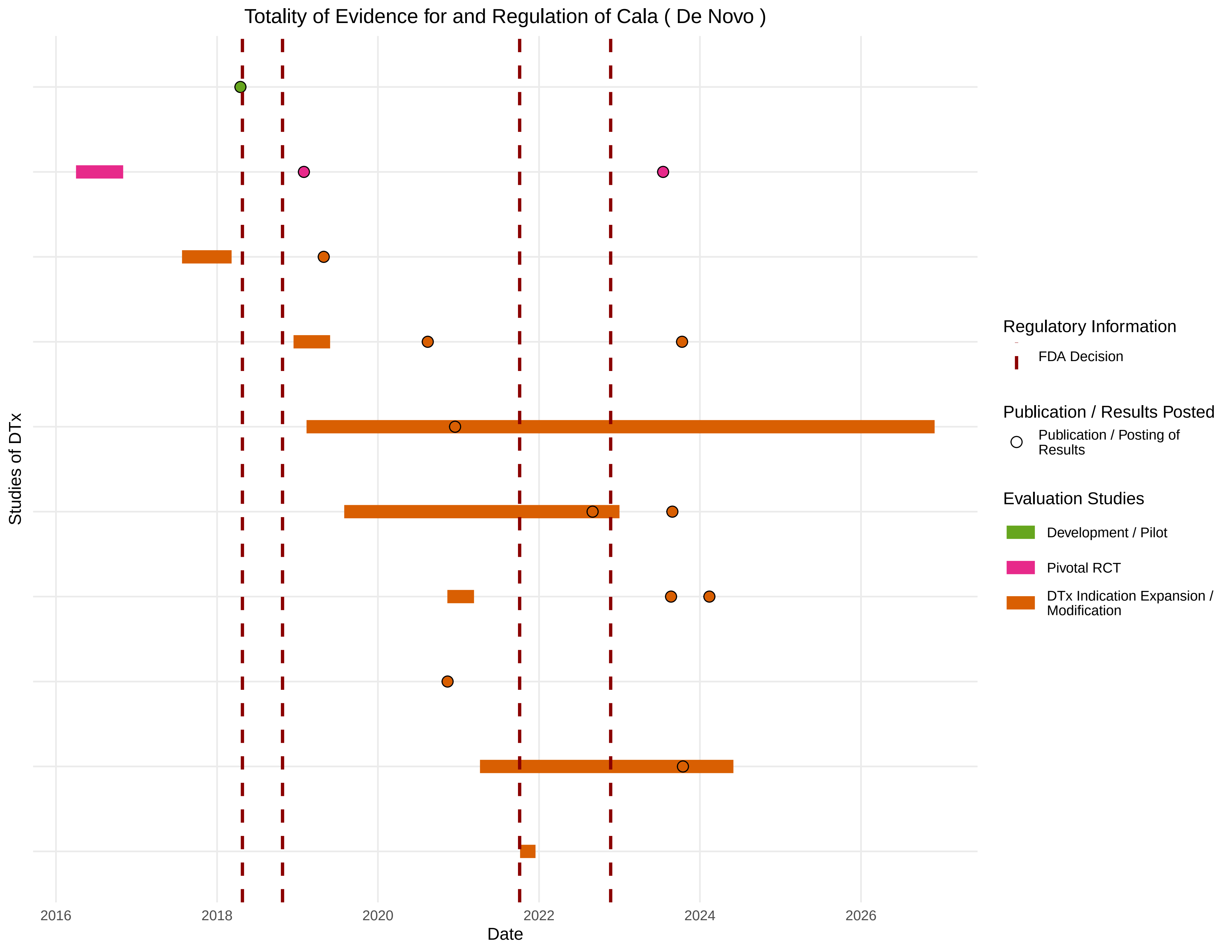

Totality of Evidence for and Regulation of Cala ( De Novo )
Studies of DTx
Date
2016
2018
2020
2022
2024
2026
Regulatory Information
FDA Decision
Publication / Results Posted
Publication / Posting of Results
Evaluation Studies
Development / Pilot
Pivotal RCT
DTx Indication Expansion / Modification

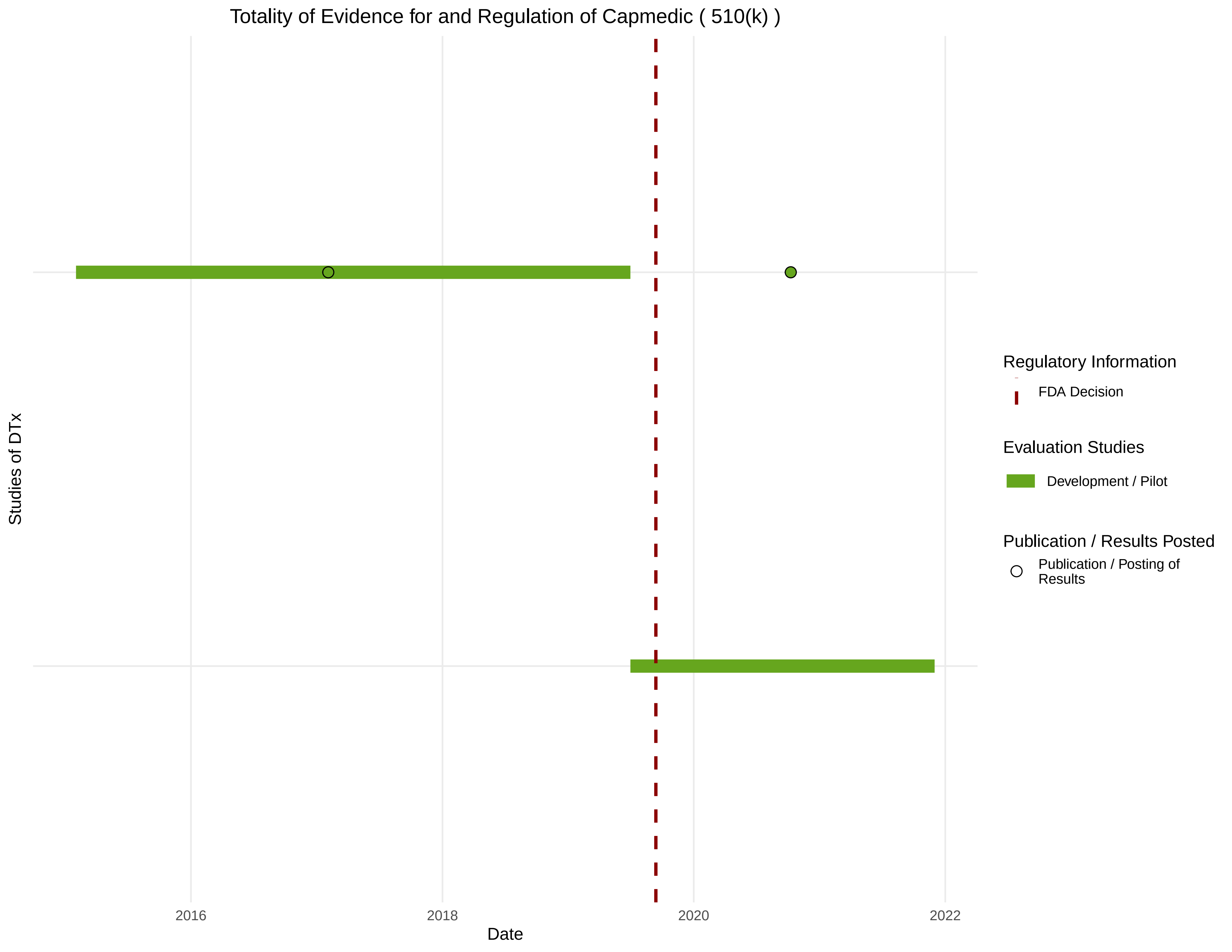

Totality of Evidence for and Regulation of Capmedic ( 510(k) )
Studies of DTx
Date
2016
2018
2020
2022
Regulatory Information
FDA Decision
Evaluation Studies
Development / Pilot
Publication / Results Posted
Publication / Posting of Results

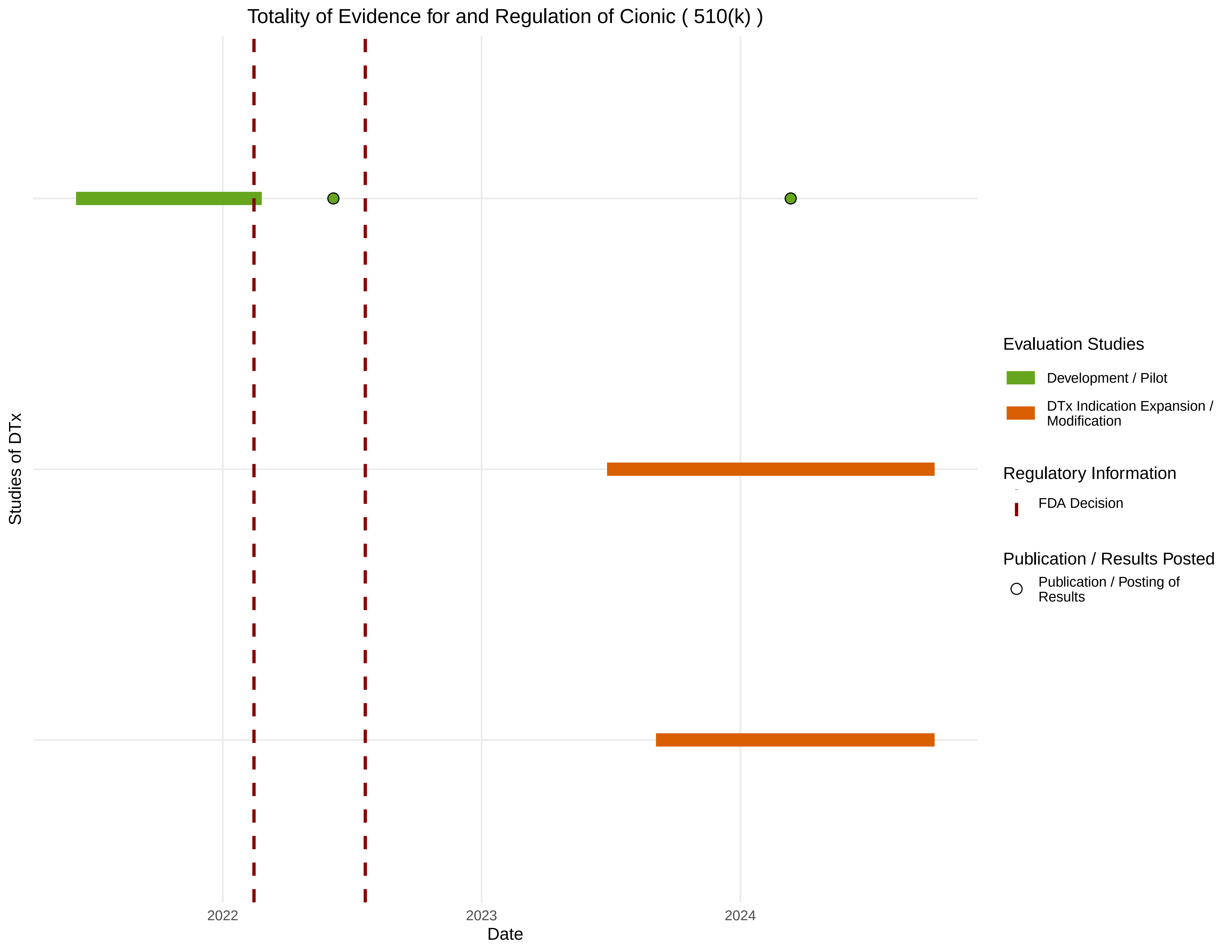

Totality of Evidence for and Regulation of Cionic ( 510(k) )
Studies of DTx
2022
2023
2024
Date
Evaluation Studies
Development / Pilot
DTx Indication Expansion / Modification
Regulatory Information
FDA Decision
Publication / Results Posted
Publication / Posting of Results

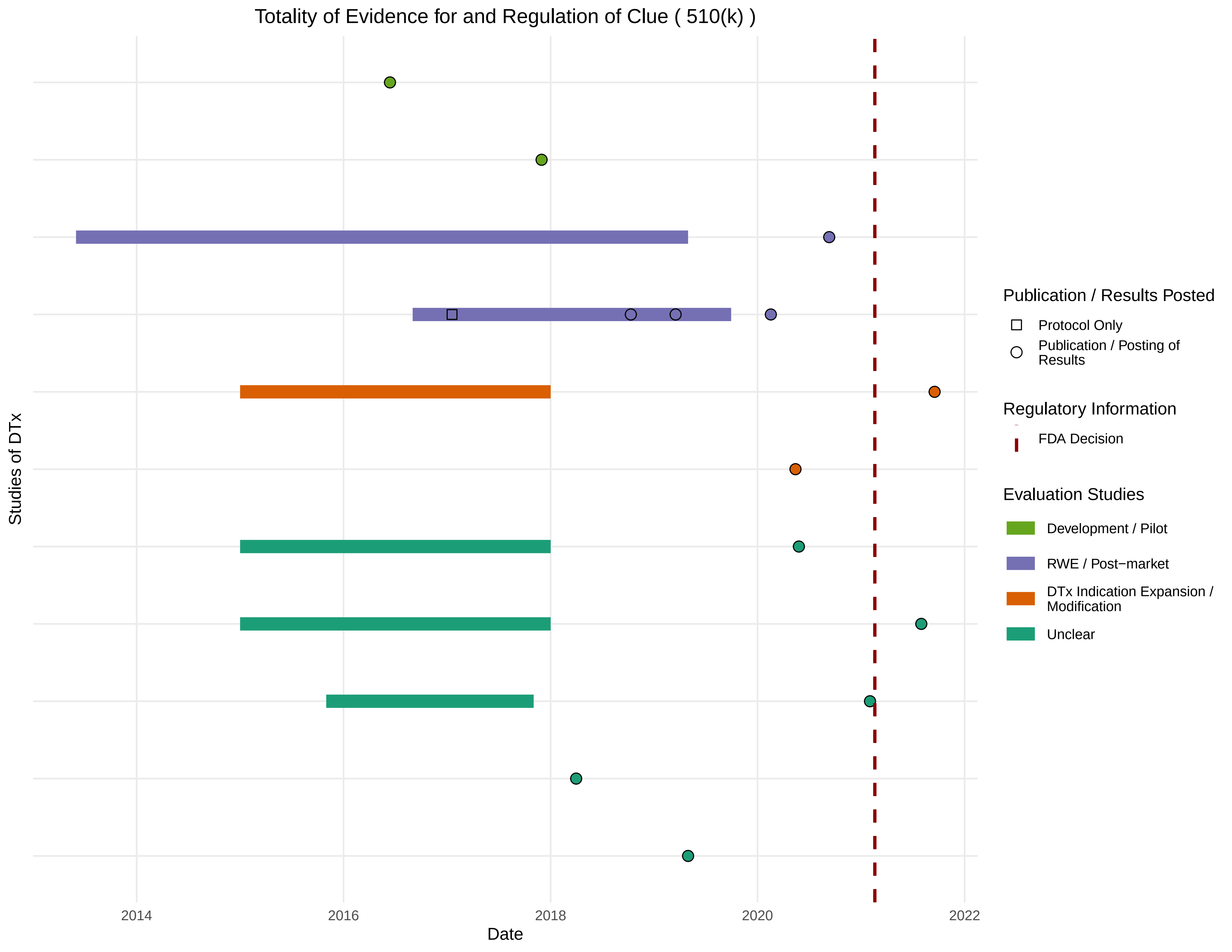
Totality of Evidence for and Regulation of Clue ( 510(k) )
Studies of DTx
2014
2016
2018
2020
2022
Date
Publication / Results Posted
Protocol Only
Publication / Posting of Results
Regulatory Information
FDA Decision
Evaluation Studies
Development / Pilot
RWE / Post−market
DTx Indication Expansion / Modification
Unclear

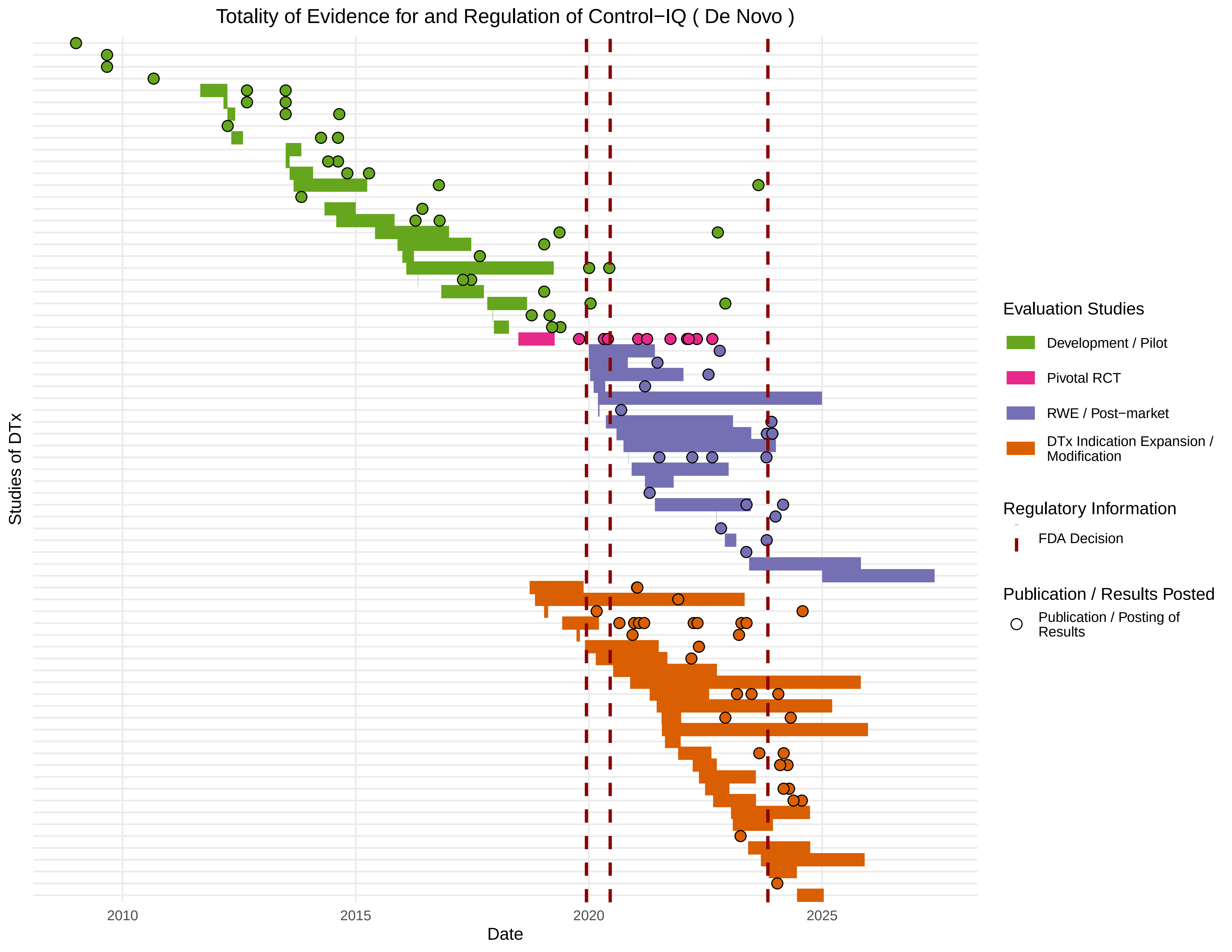
Totality of Evidence for and Regulation of Control−IQ ( De Novo )
Studies of DTx
Date
2010
2015
2020
2025
Evaluation Studies
Development / Pilot
Pivotal RCT
RWE / Post−market
DTx Indication Expansion / Modification
Regulatory Information
FDA Decision
Publication / Results Posted
Publication / Posting of Results

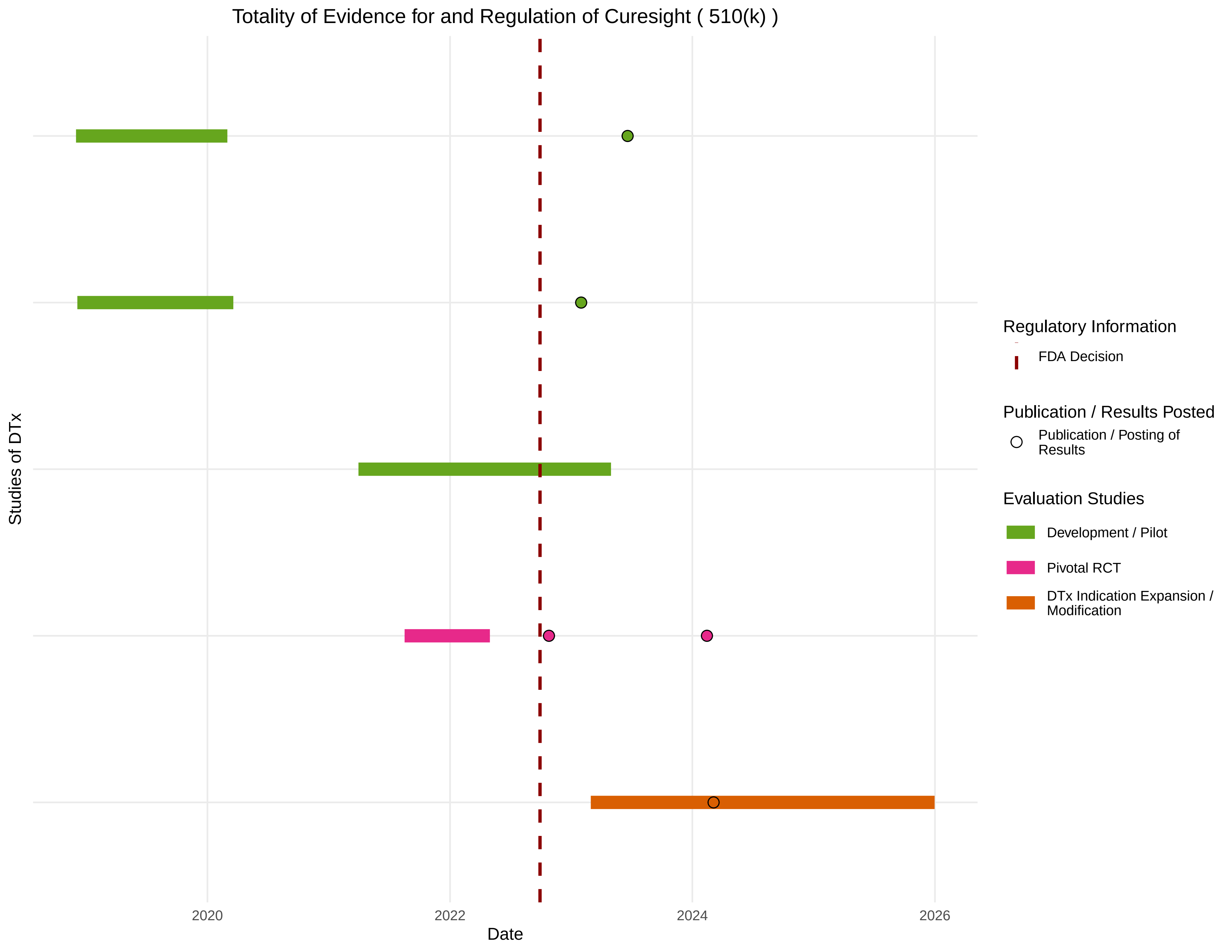
Totality of Evidence for and Regulation of Curesight ( 510(k) )
Studies of DTx
2020
2022
2024
2026
Date
Regulatory Information
FDA Decision
Publication / Results Posted
Publication / Posting of Results
Evaluation Studies
Development / Pilot
Pivotal RCT
DTx Indication Expansion / Modification

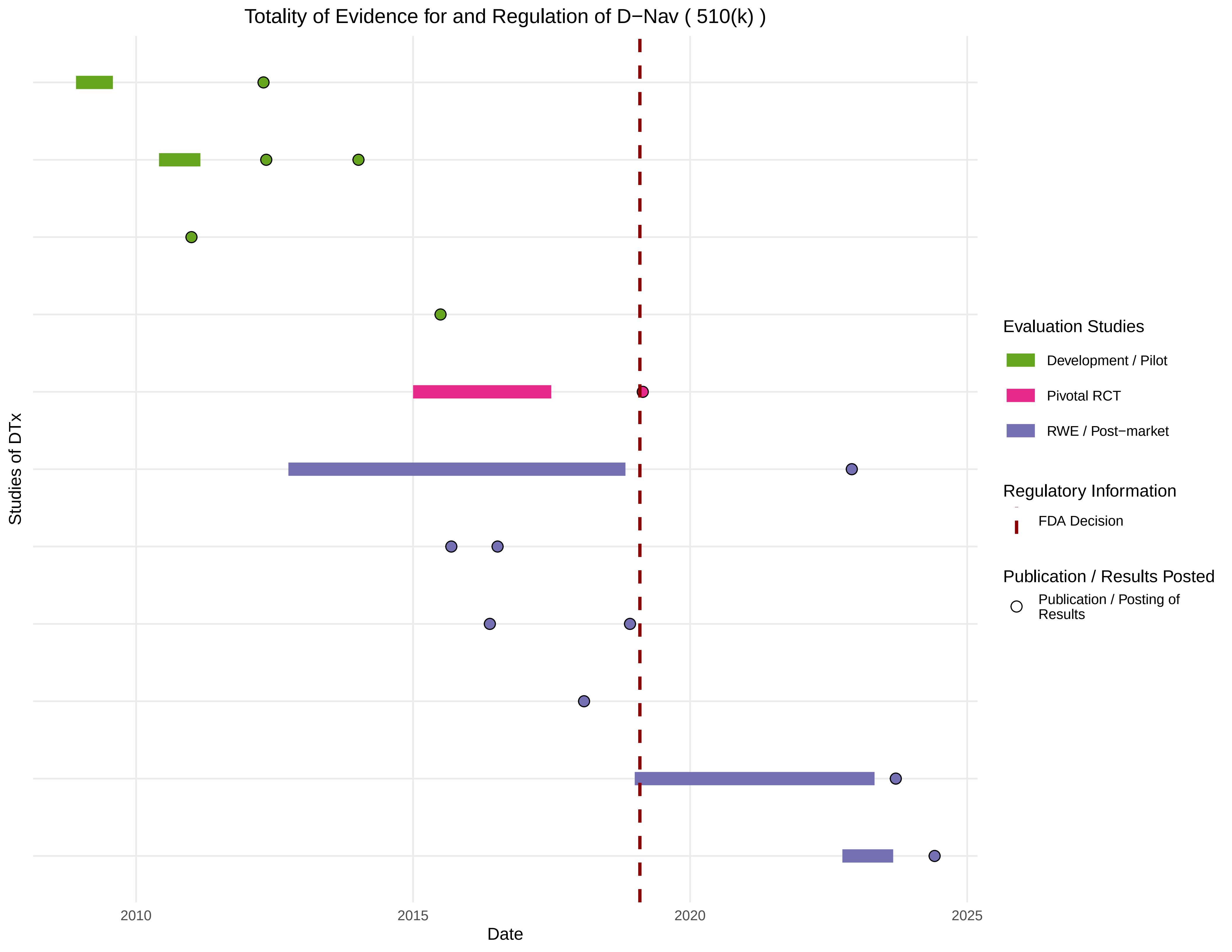

Totality of Evidence for and Regulation of D−Nav ( 510(k) )
Studies of DTx
Date
2010
2015
2020
2025
Evaluation Studies
Development / Pilot
Pivotal RCT
RWE / Post−market
Regulatory Information
FDA Decision
Publication / Results Posted
Publication / Posting of Results

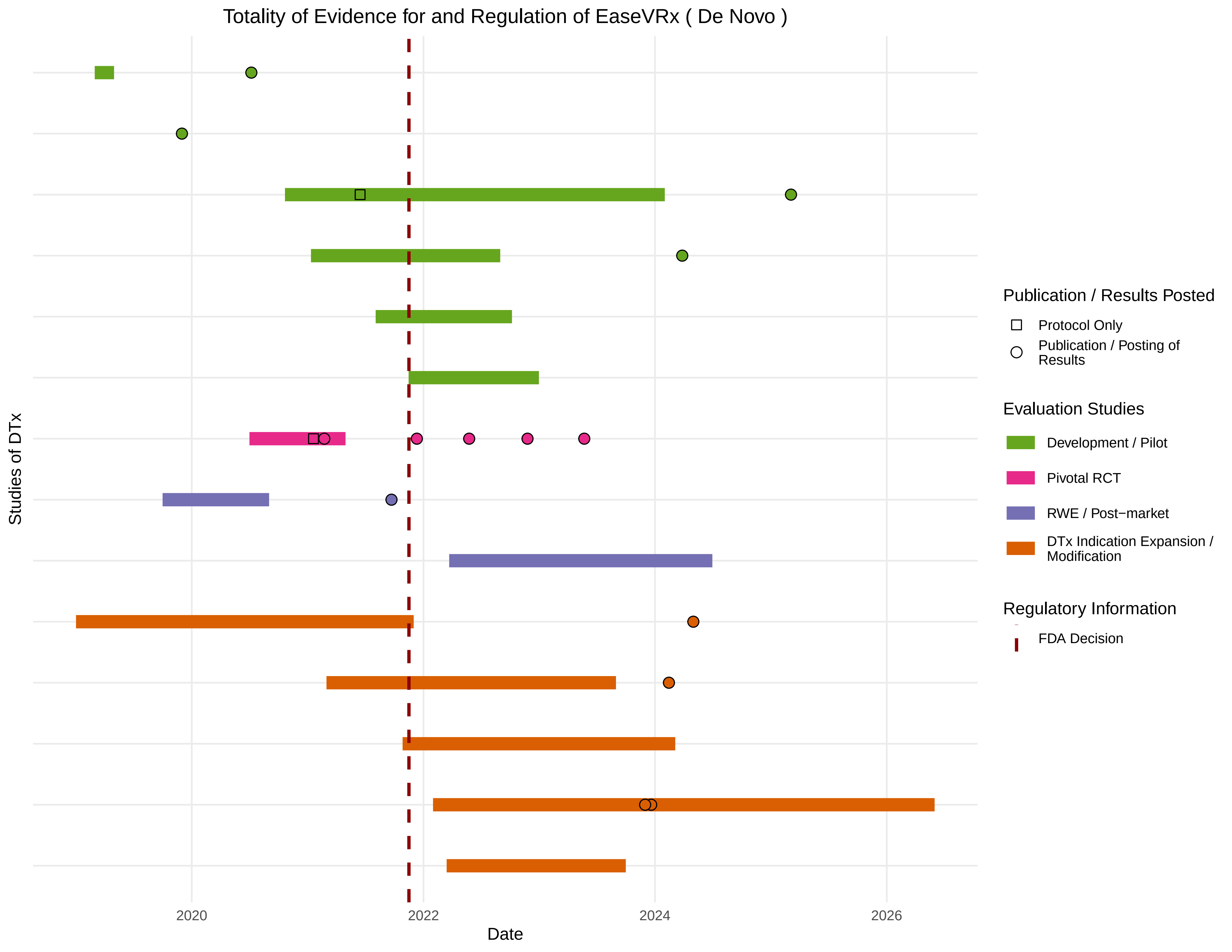

Totality of Evidence for and Regulation of EaseVRx ( De Novo )
Studies of DTx
Date
2020
2022
2024
2026
Publication / Results Posted
Protocol Only
Publication / Posting of Results
Evaluation Studies
Development / Pilot
Pivotal RCT
RWE / Post−market
DTx Indication Expansion / Modification
Regulatory Information
FDA Decision

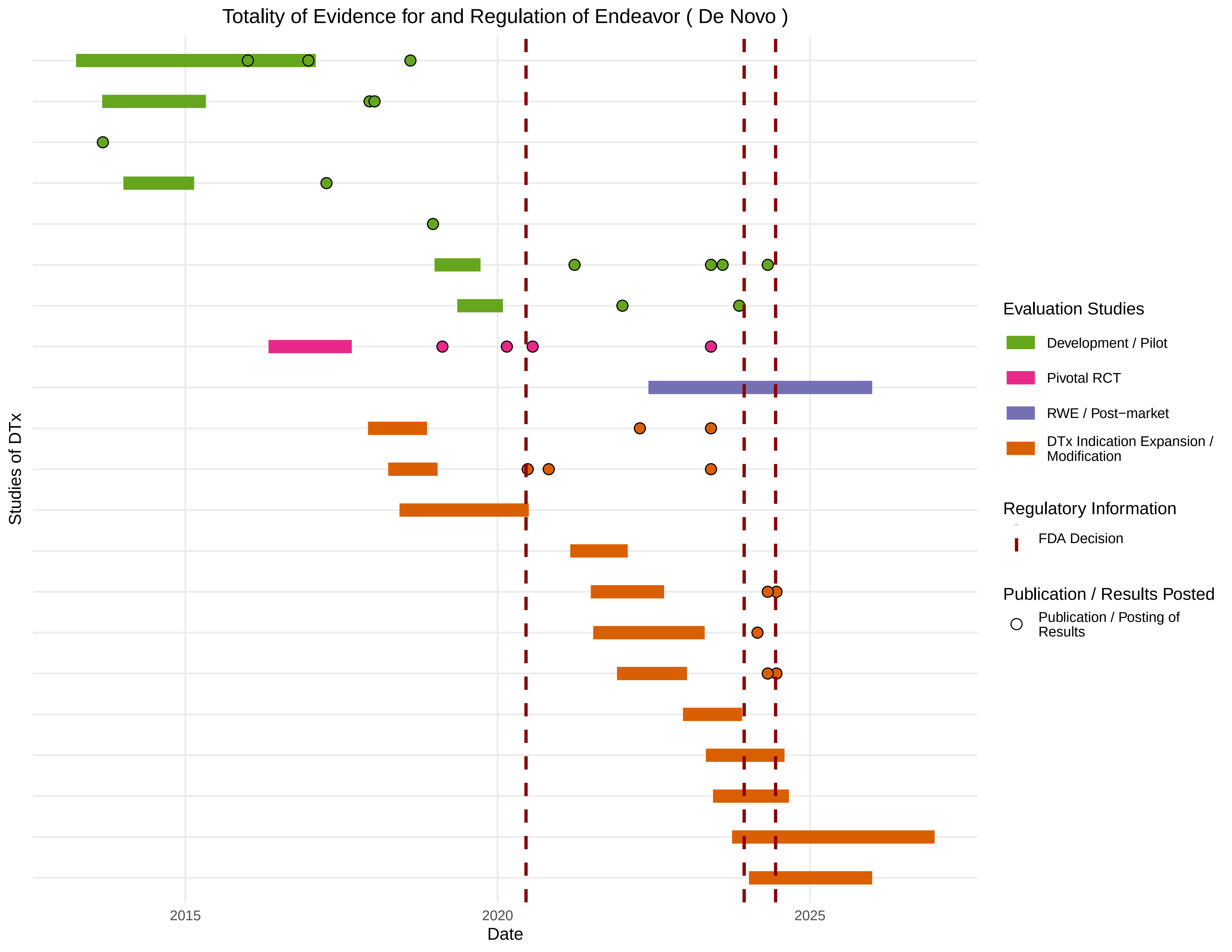
Totality of Evidence for and Regulation of Endeavor ( De Novo )
Studies of DTx
2015
2020
2025
Date
Evaluation Studies
Development / Pilot
Pivotal RCT
RWE / Post−market
DTx Indication Expansion / Modification
Regulatory Information
FDA Decision
Publication / Results Posted
Publication / Posting of Results

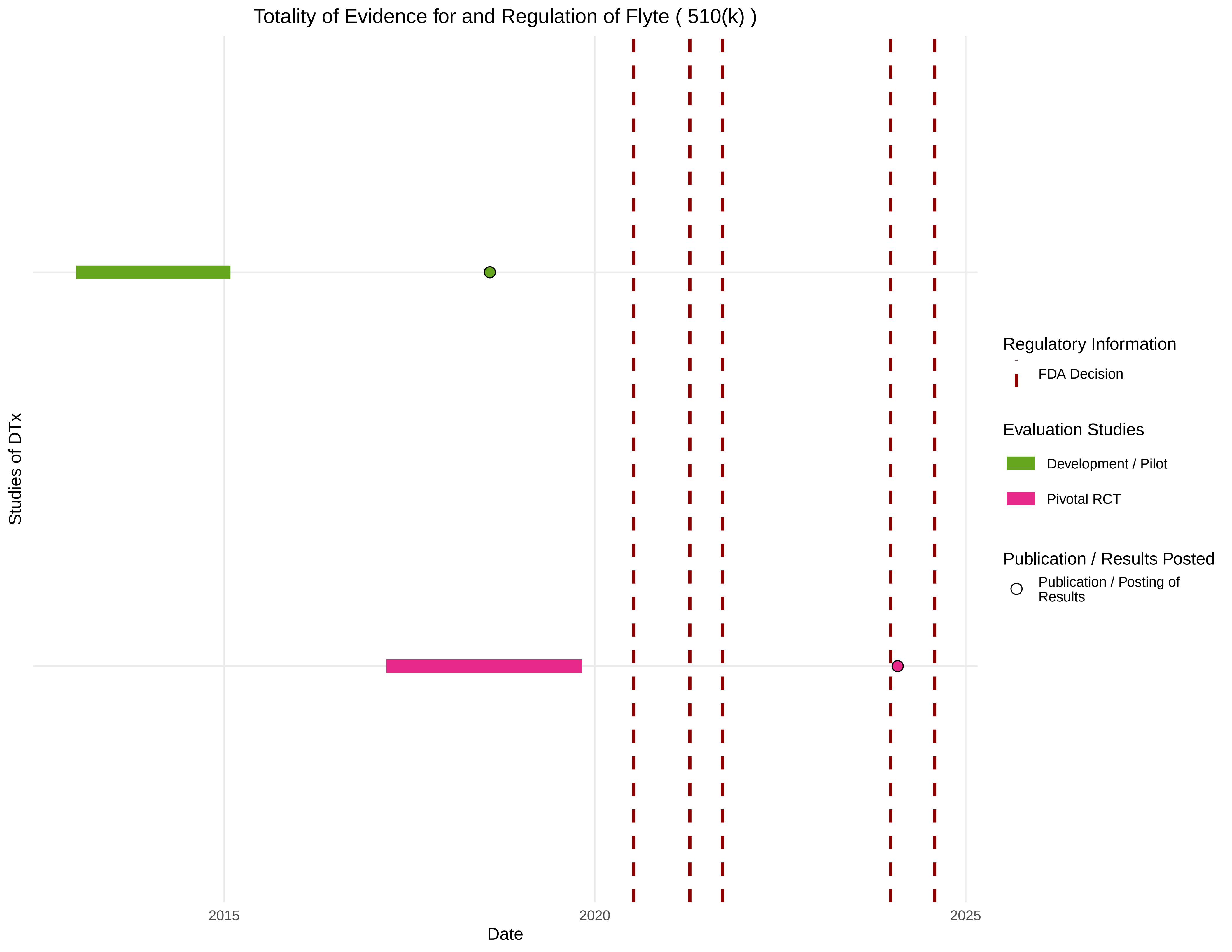

Totality of Evidence for and Regulation of Flyte ( 510(k) )
Studies of DTx
2015
2020
2025
Date
Regulatory Information
FDA Decision
Evaluation Studies
Development / Pilot
Pivotal RCT
Publication / Results Posted
Publication / Posting of Results

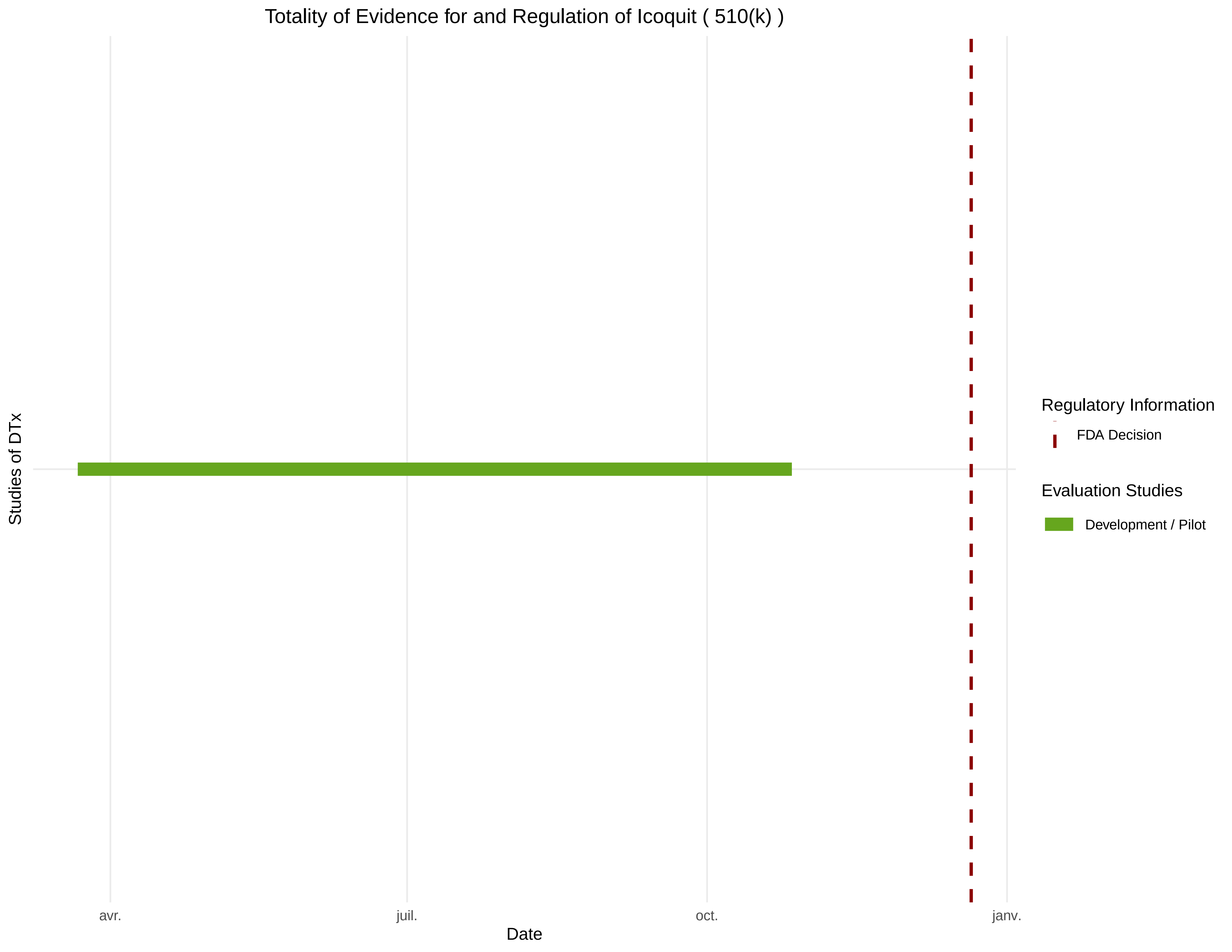
Totality of Evidence for and Regulation of Icoquit ( 510(k) )
Studies of DTx
avr.
juil.
oct.
janv.
Date
Regulatory Information
FDA Decision
Evaluation Studies
Development / Pilot

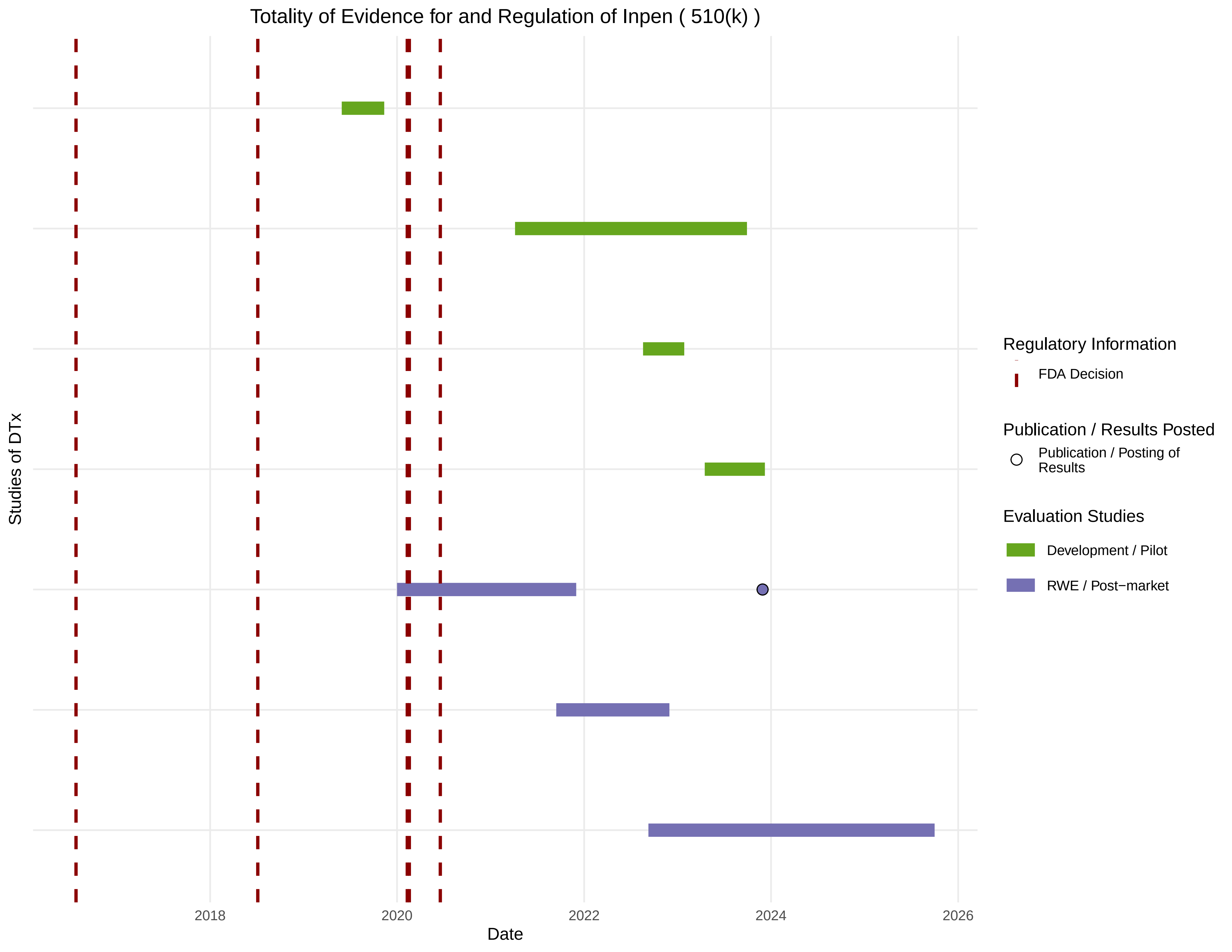
Totality of Evidence for and Regulation of Inpen ( 510(k) )
Studies of DTx
2018
2020
2022
2024
2026
Date
Regulatory Information
FDA Decision
Publication / Results Posted
Publication / Posting of Results
Evaluation Studies
Development / Pilot
RWE / Post−market

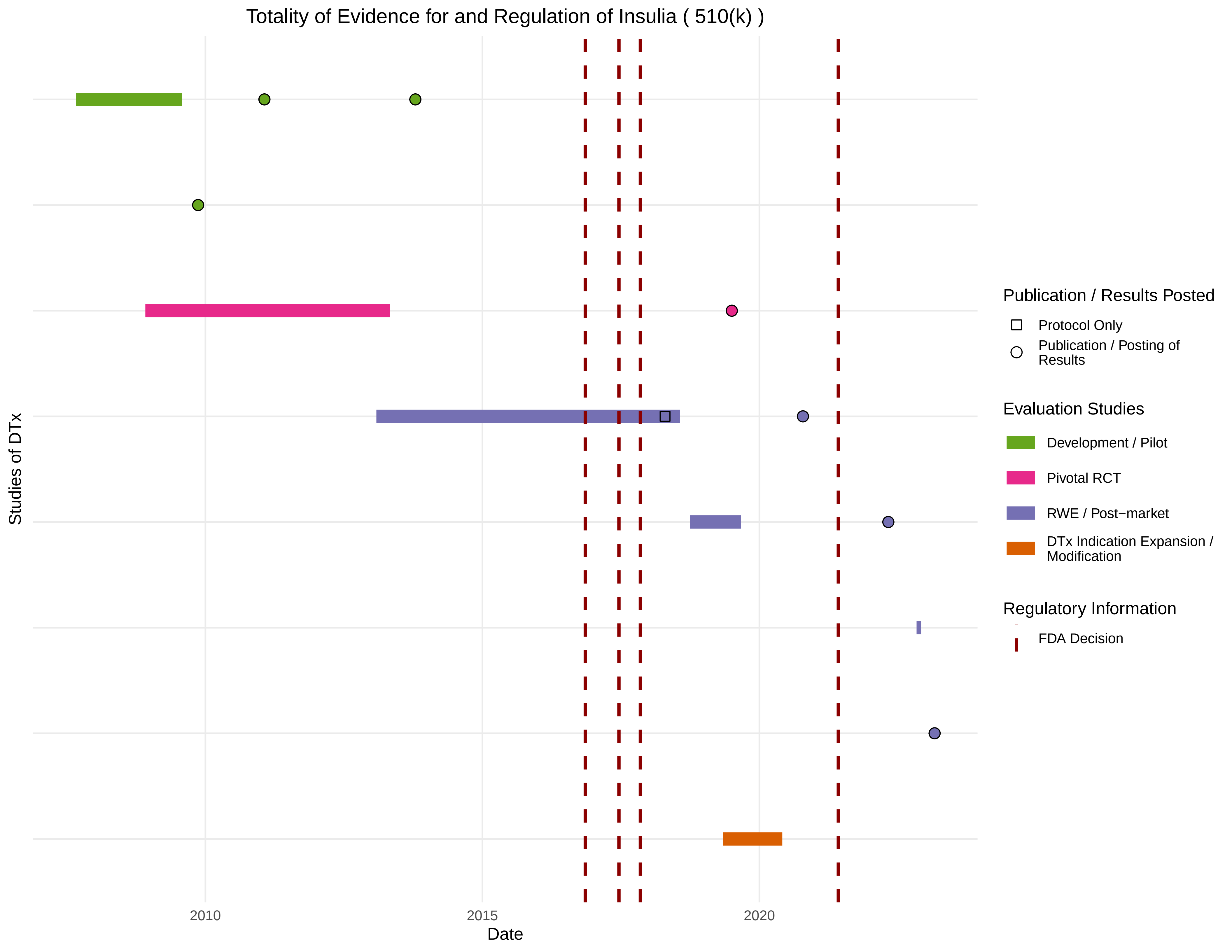
Totality of Evidence for and Regulation of Insulia ( 510(k) )
Studies of DTx
2010
2015
2020
Date
Publication / Results Posted
Protocol Only
Publication / Posting of Results
Evaluation Studies
Development / Pilot
Pivotal RCT
RWE / Post−market
DTx Indication Expansion / Modification
Regulatory Information
FDA Decision

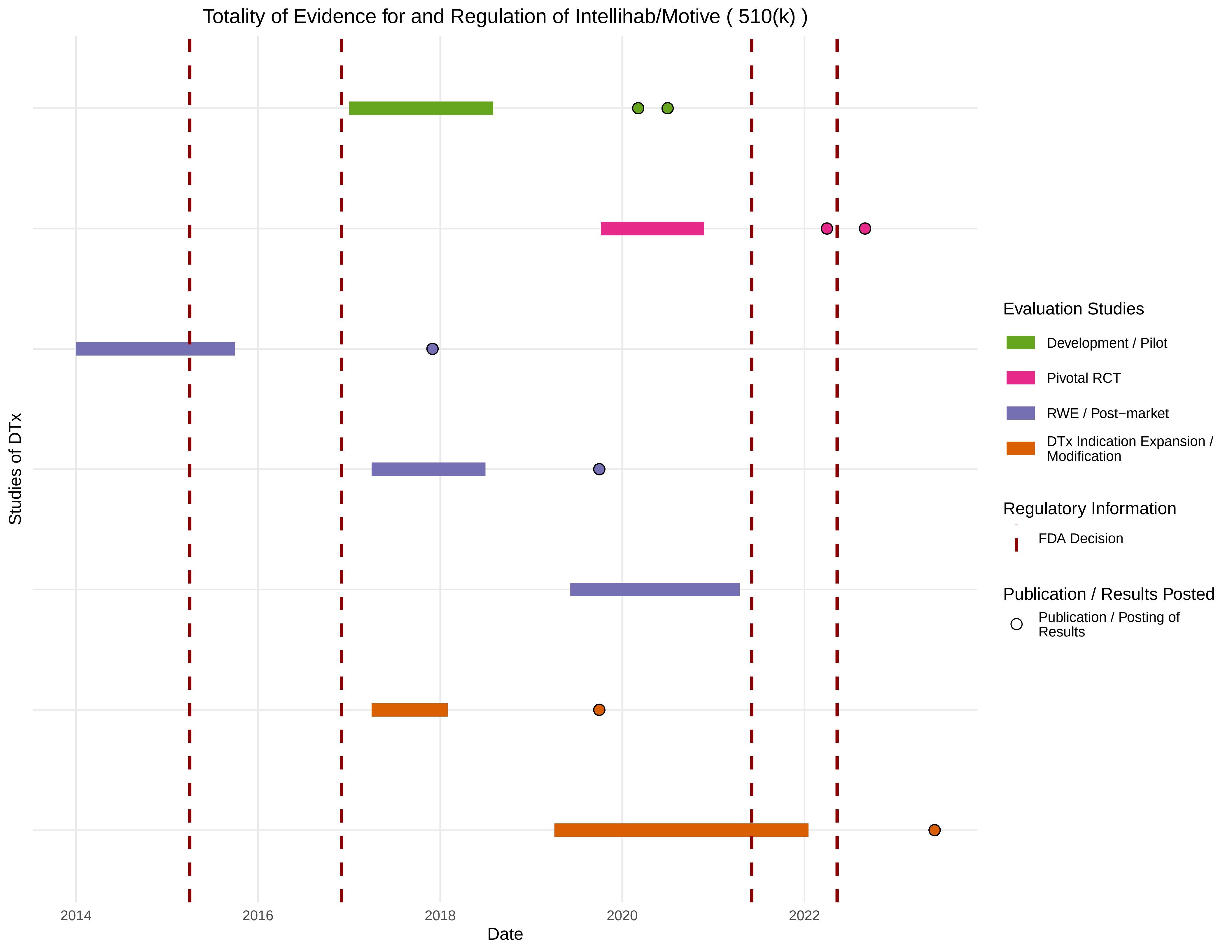
Totality of Evidence for and Regulation of Intellihab/Motive ( 510(k) )
Studies of DTx
Date
2014
2016
2018
2020
2022
Evaluation Studies
Development / Pilot
Pivotal RCT
RWE / Post−market
DTx Indication Expansion / Modification
Regulatory Information
FDA Decision
Publication / Results Posted
Publication / Posting of Results

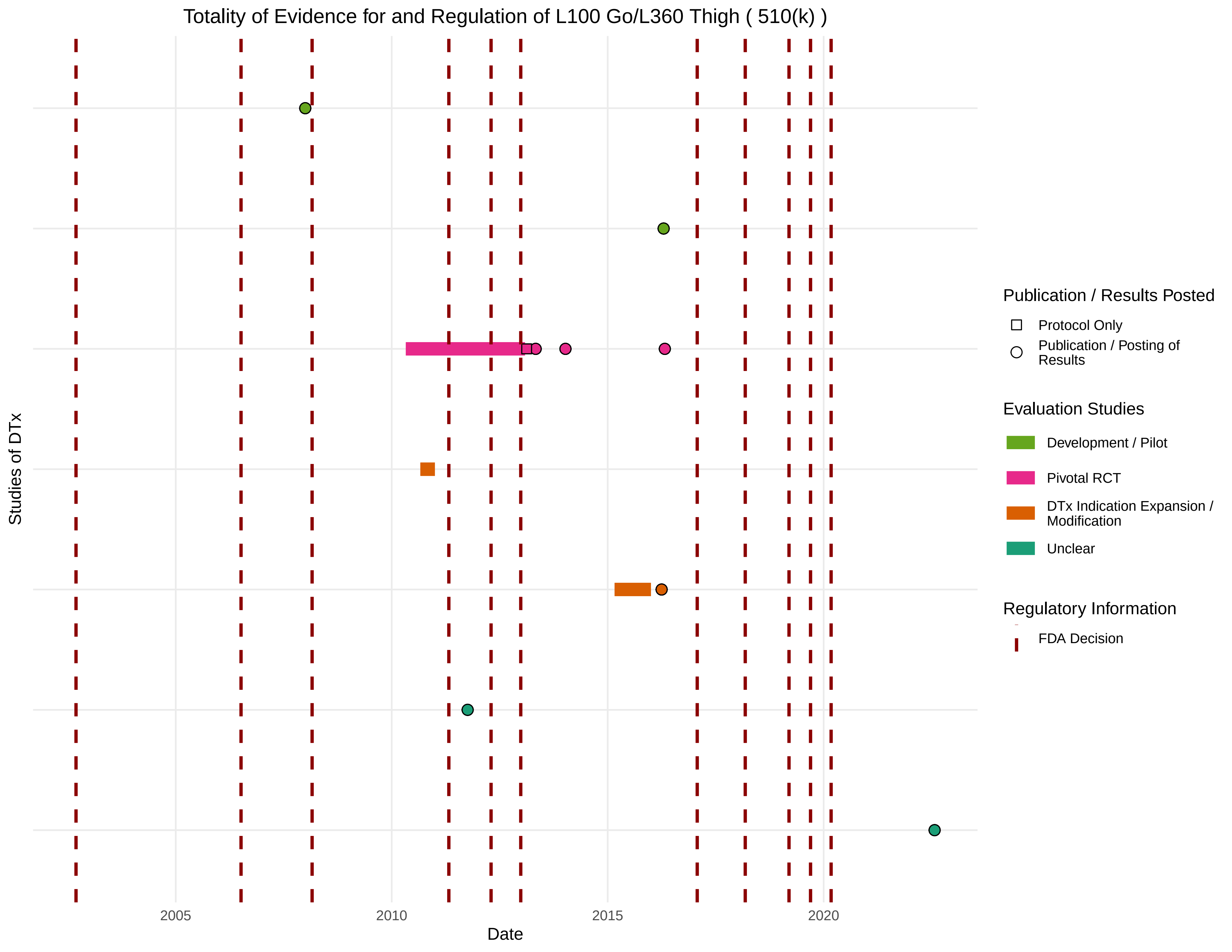
Totality of Evidence for and Regulation of L100 Go/L360 Thigh ( 510(k) )
Studies of DTx
Date
2005
2010
2015
2020
Publication / Results Posted
Protocol Only
Publication / Posting of Results
Evaluation Studies
Development / Pilot
Pivotal RCT
DTx Indication Expansion / Modification
Unclear
Regulatory Information
FDA Decision

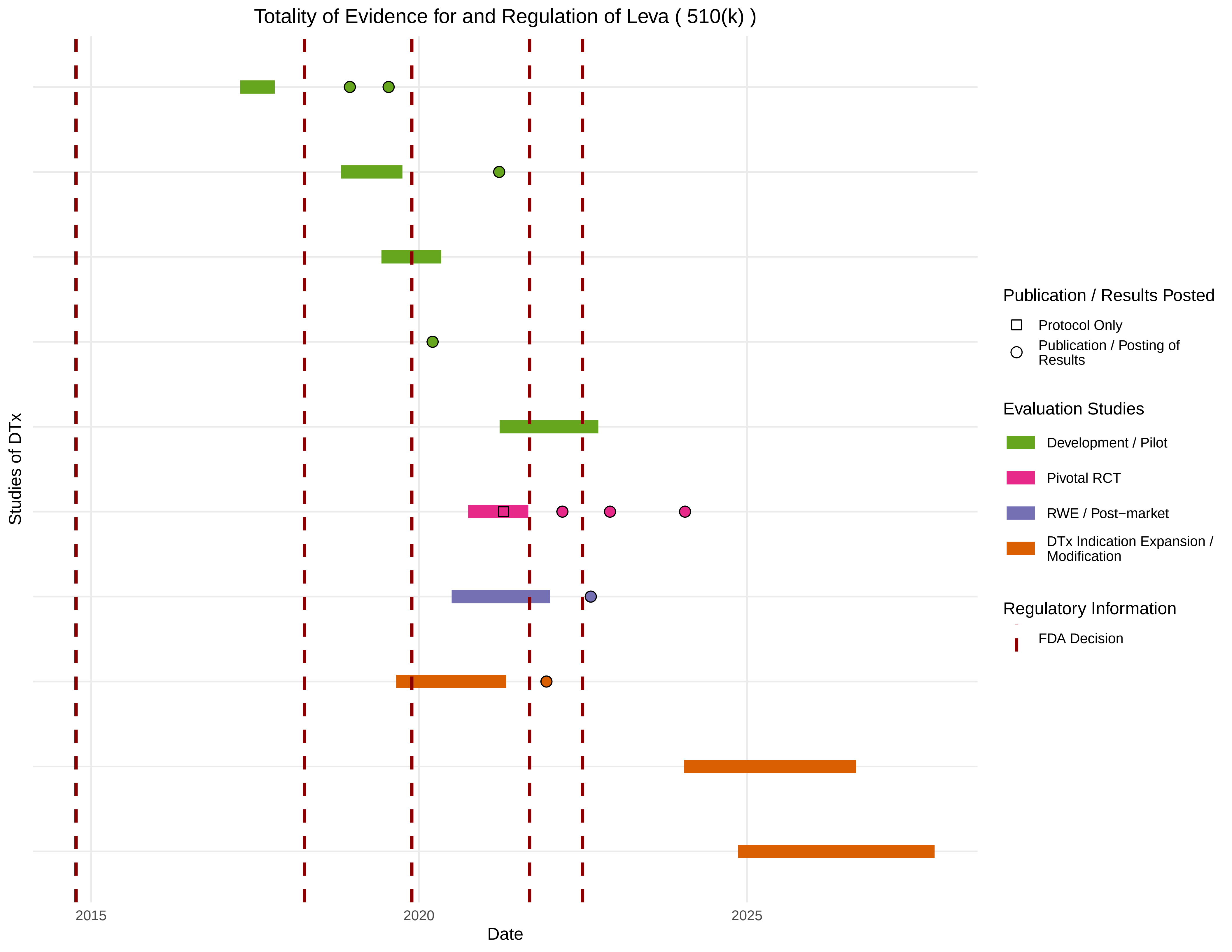

Totality of Evidence for and Regulation of Leva ( 510(k) )
Studies of DTx
Date
2015
2020
2025
Publication / Results Posted
Protocol Only
Publication / Posting of Results
Evaluation Studies
Development / Pilot
Pivotal RCT
RWE / Post−market
DTx Indication Expansion / Modification
Regulatory Information
FDA Decision

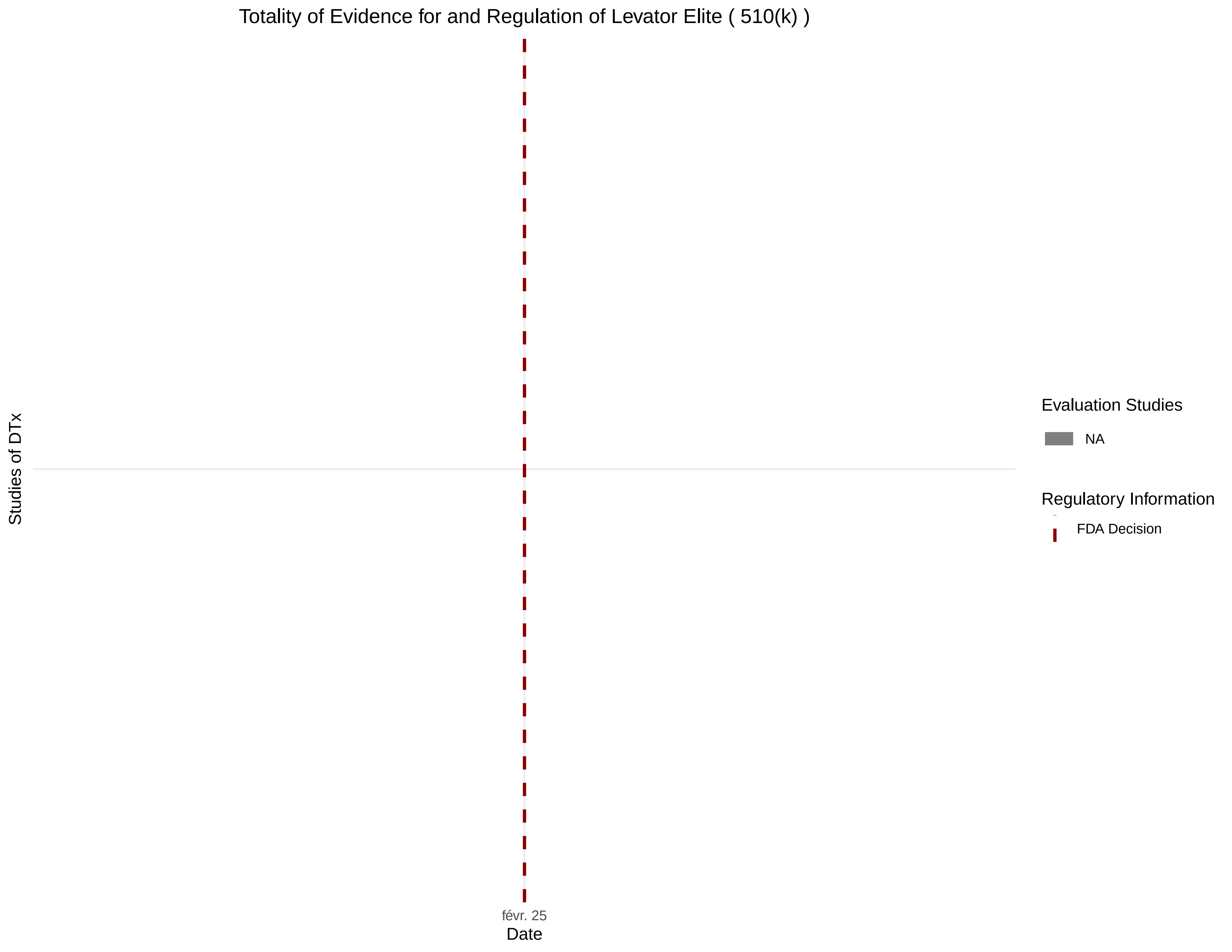
Totality of Evidence for and Regulation of Levator Elite ( 510(k) )
Studies of DTx
févr. 25
Date
Evaluation Studies
NA
Regulatory Information
FDA Decision

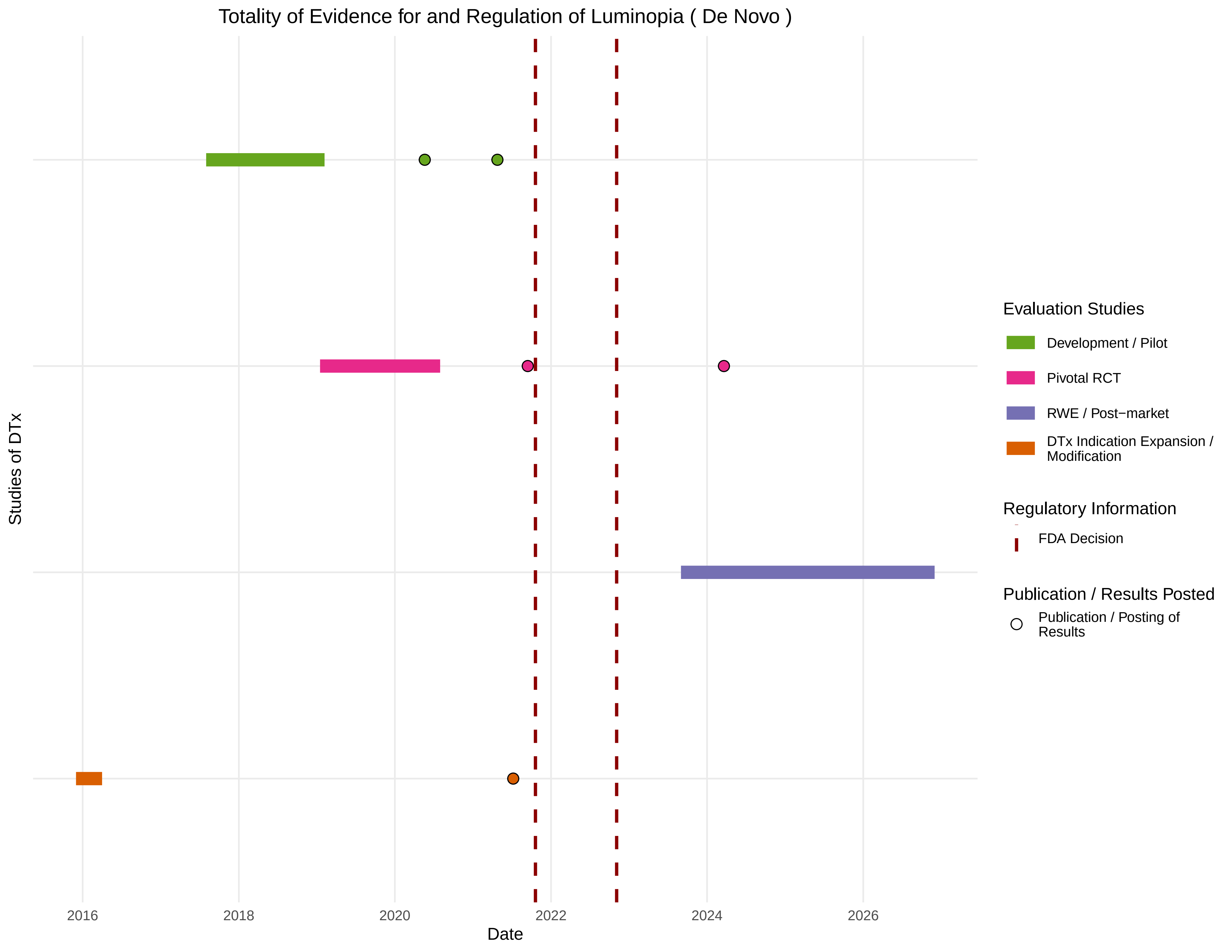

Totality of Evidence for and Regulation of Luminopia ( De Novo )
Studies of DTx
Date
2016
2018
2020
2022
2024
2026
Evaluation Studies
Development / Pilot
Pivotal RCT
RWE / Post−market
DTx Indication Expansion / Modification
Regulatory Information
FDA Decision
Publication / Results Posted
Publication / Posting of Results

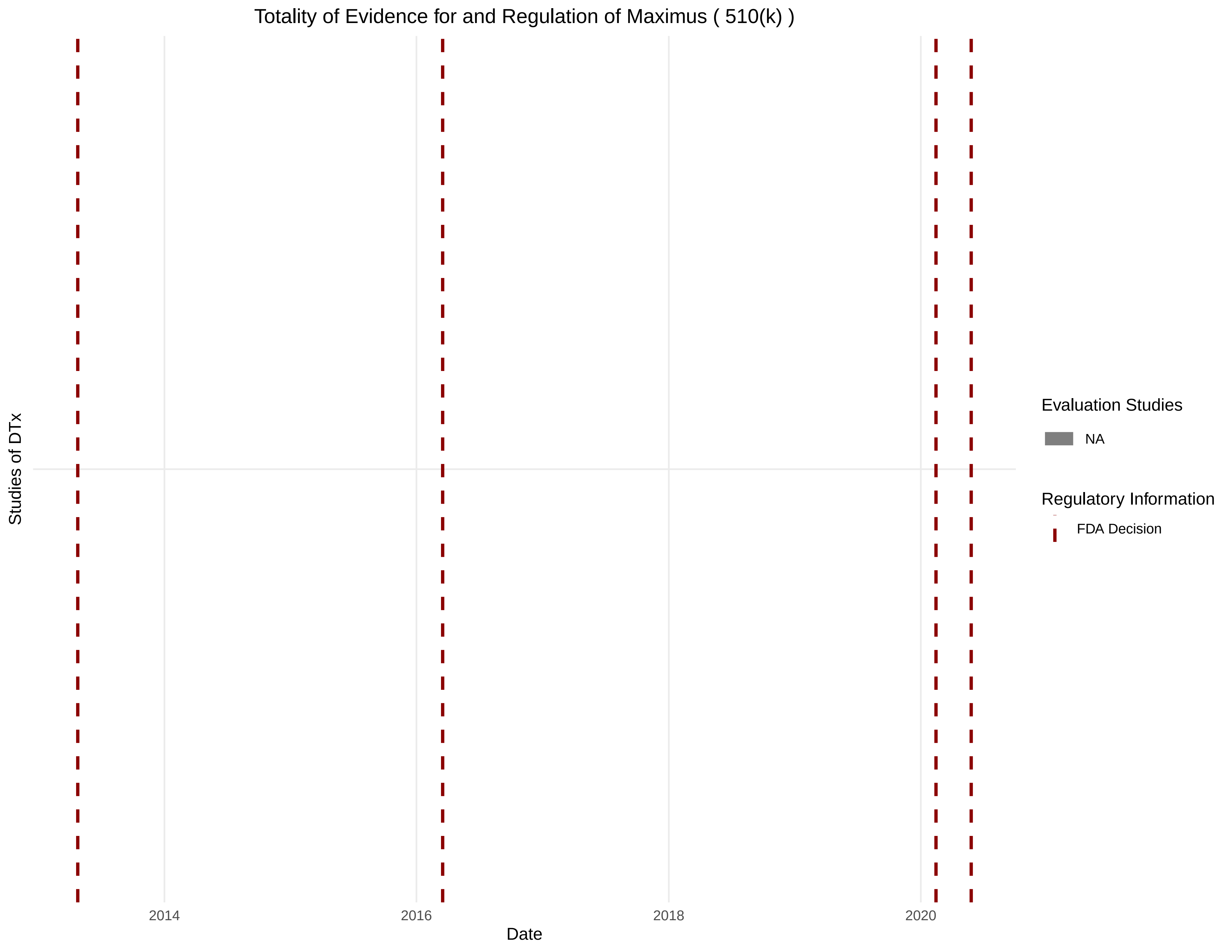
Totality of Evidence for and Regulation of Maximus ( 510(k) )
Studies of DTx
2014
2016
2018
2020
Date
Evaluation Studies
NA
Regulatory Information
FDA Decision

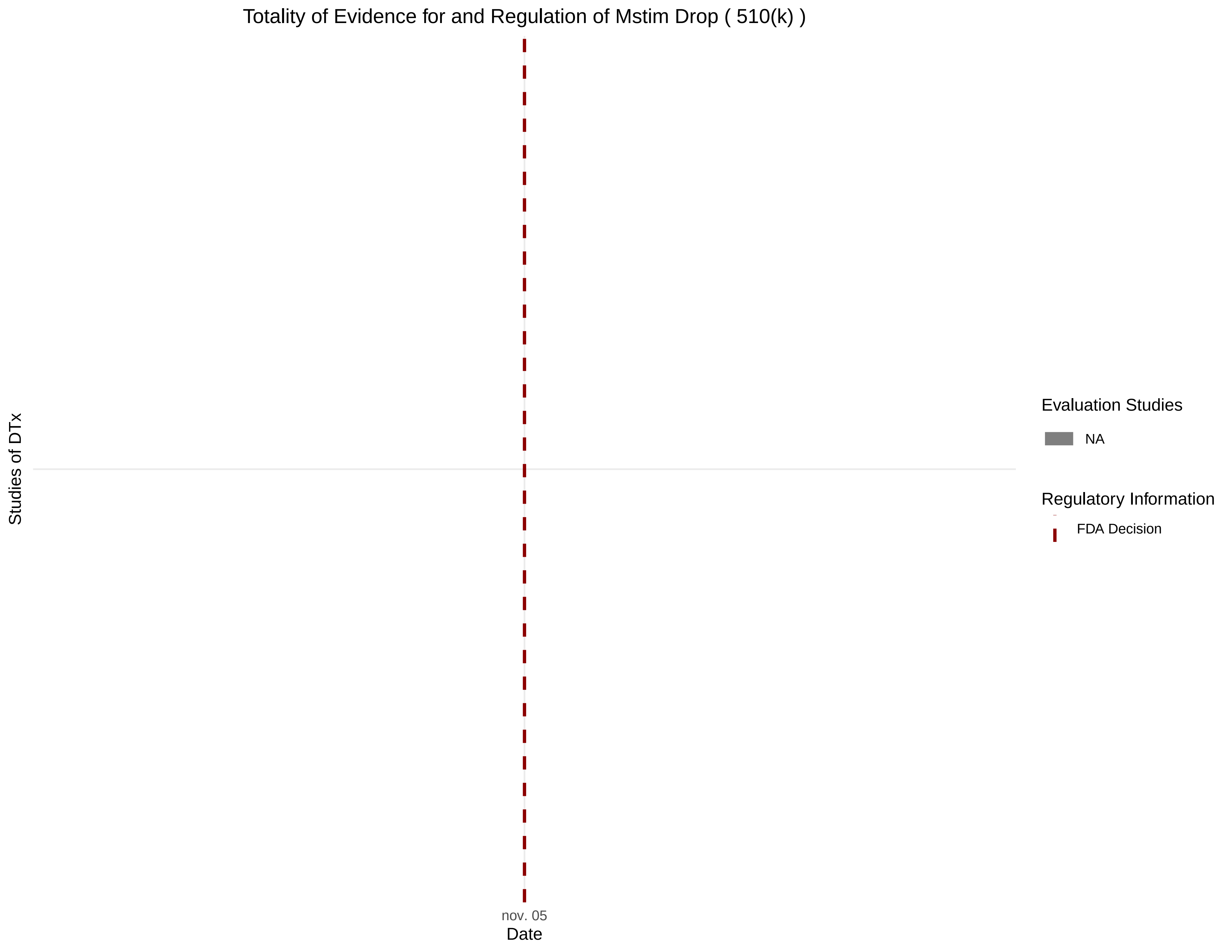
Totality of Evidence for and Regulation of Mstim Drop ( 510(k) )
Studies of DTx
nov. 05
Date
Evaluation Studies
NA
Regulatory Information
FDA Decision

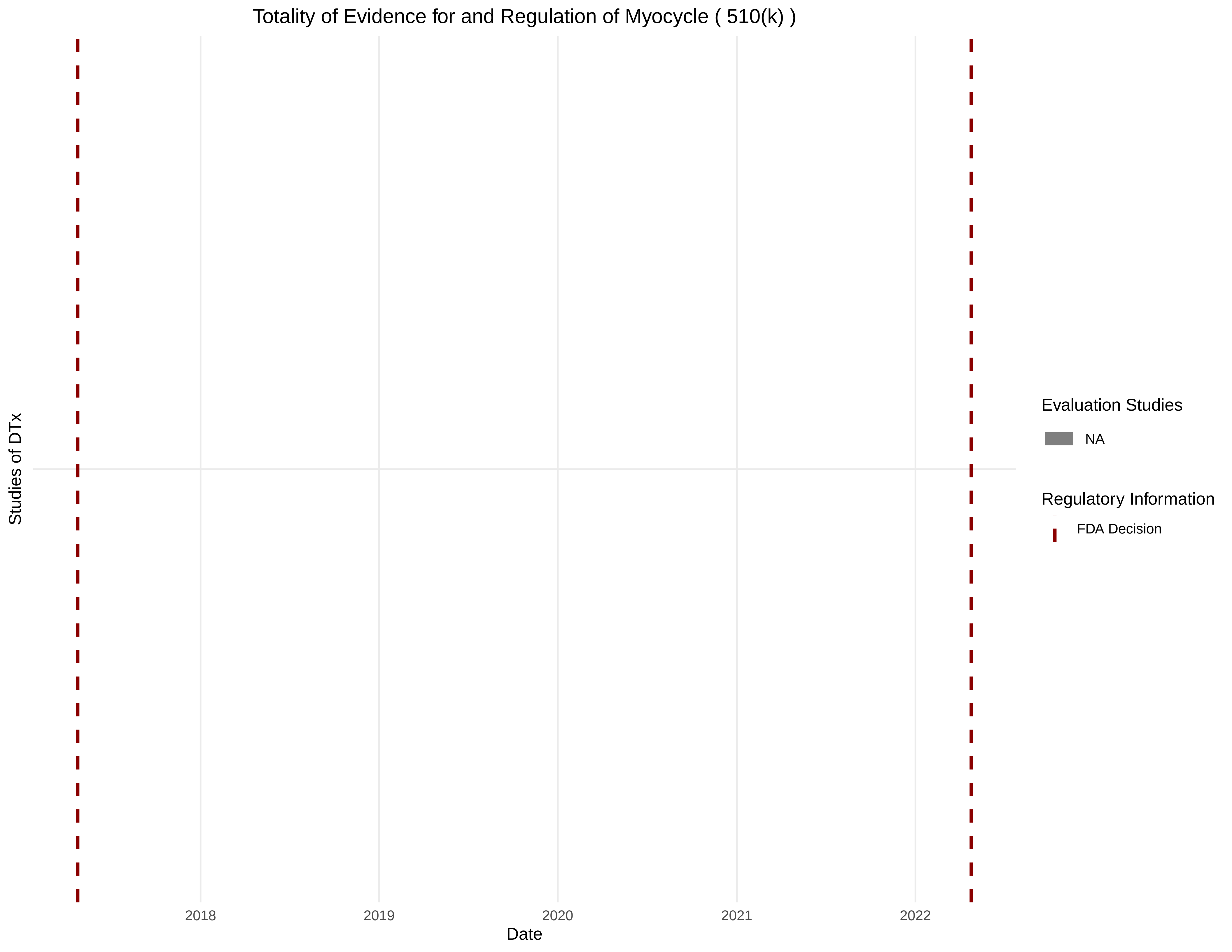

Totality of Evidence for and Regulation of Myocycle ( 510(k) )
Studies of DTx
2018
2019
2020
2021
2022
Date
Evaluation Studies
NA
Regulatory Information
FDA Decision

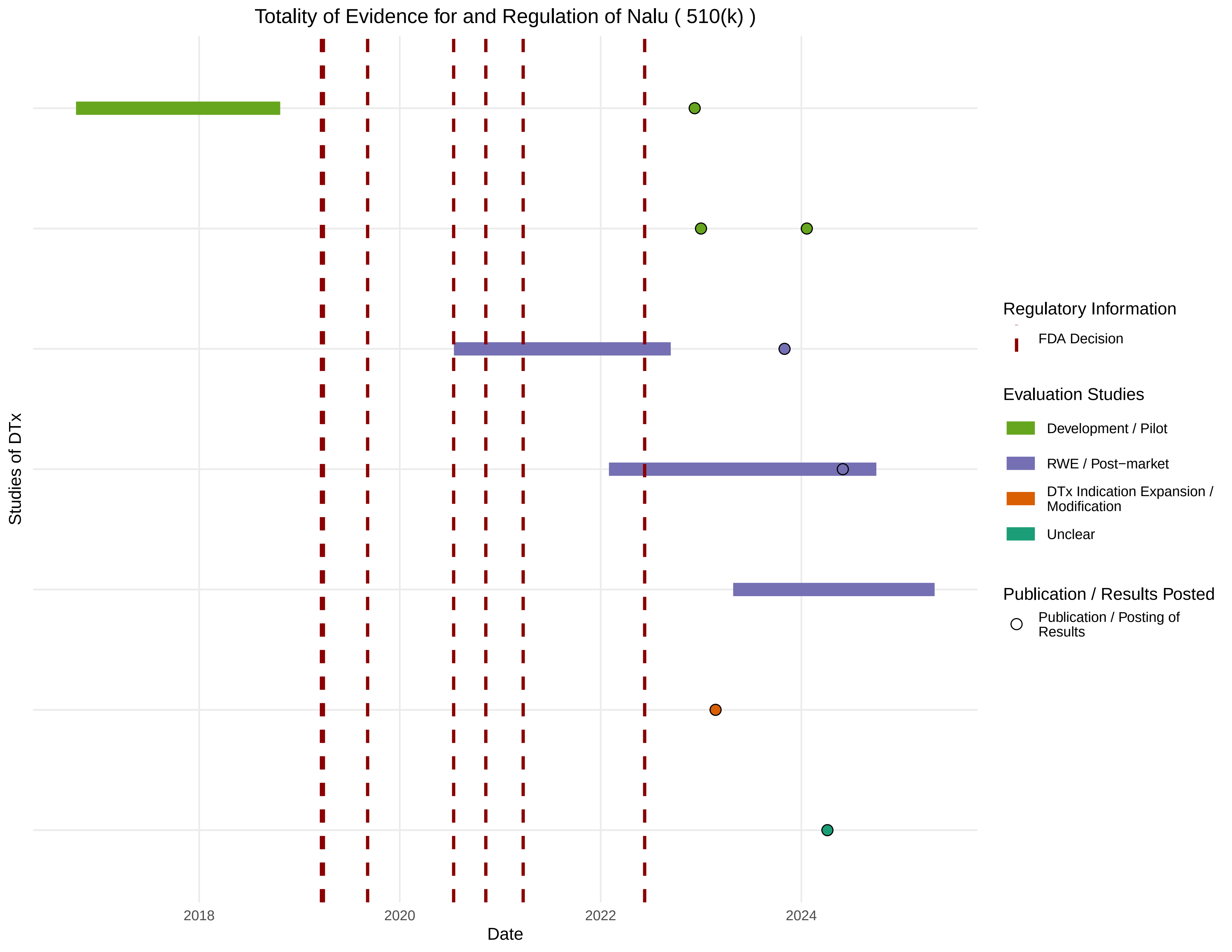

Totality of Evidence for and Regulation of Nalu ( 510(k) )
Studies of DTx
2018
2020
2022
2024
Date
Regulatory Information
FDA Decision
Evaluation Studies
Development / Pilot
RWE / Post−market
DTx Indication Expansion / Modification
Unclear
Publication / Results Posted
Publication / Posting of Results

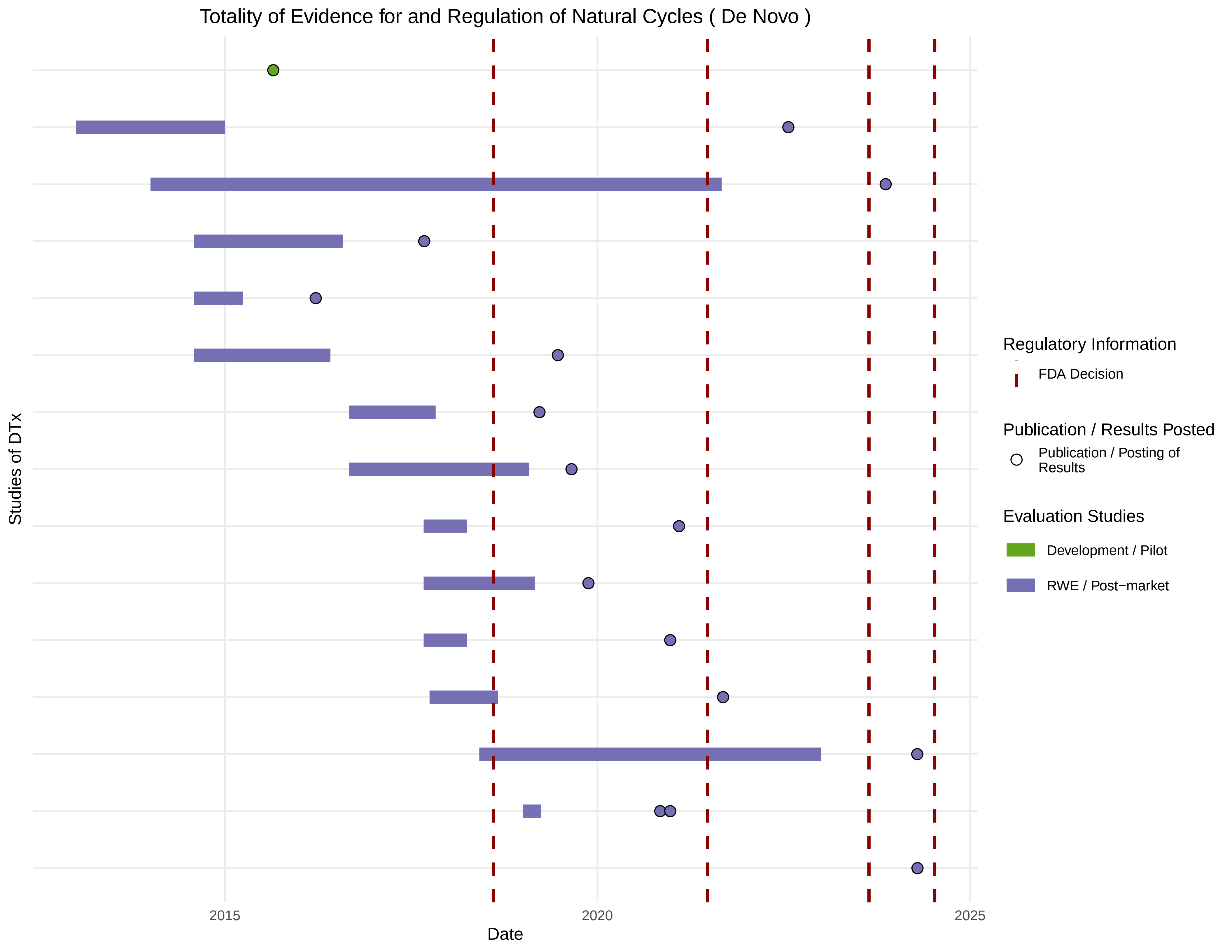

Totality of Evidence for and Regulation of Natural Cycles ( De Novo )
Studies of DTx
Date
2015
2020
2025
Regulatory Information
FDA Decision
Publication / Results Posted
Publication / Posting of Results
Evaluation Studies
Development / Pilot
RWE / Post−market

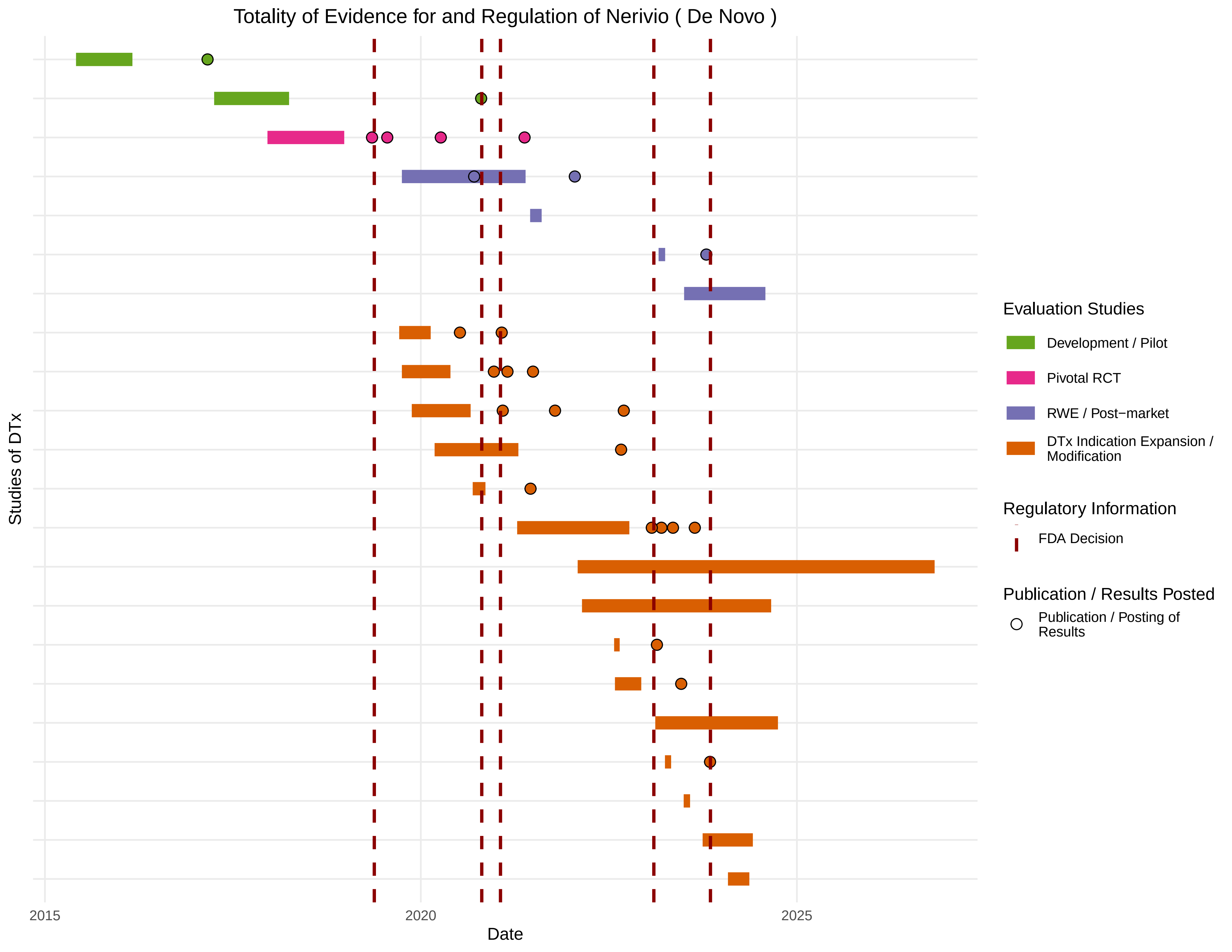

Totality of Evidence for and Regulation of Nerivio ( De Novo )
Studies of DTx
Date
2015
2020
2025
Evaluation Studies
Development / Pilot
Pivotal RCT
RWE / Post−market
DTx Indication Expansion / Modification
Regulatory Information
FDA Decision
Publication / Results Posted
Publication / Posting of Results

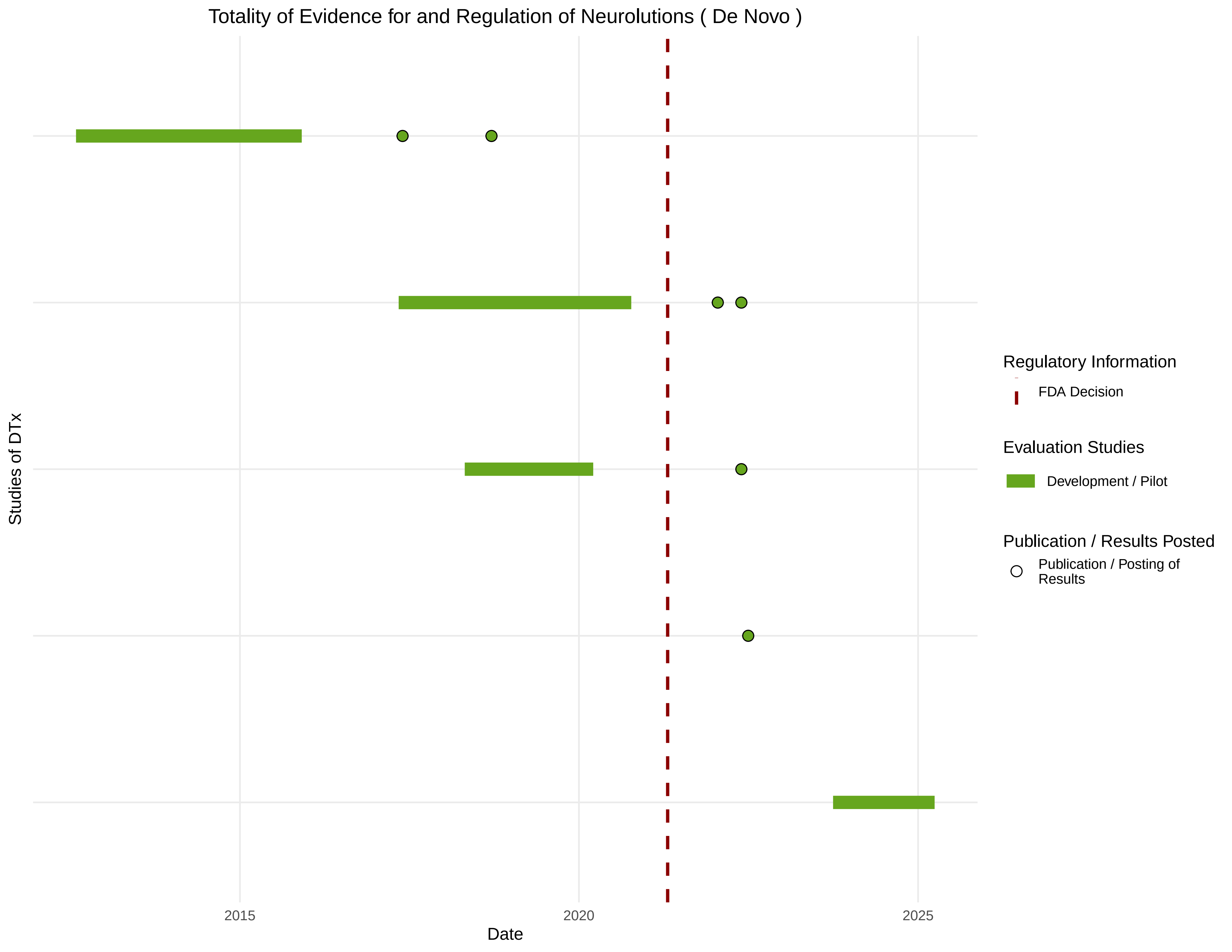

Totality of Evidence for and Regulation of Neurolutions ( De Novo )
Studies of DTx
Date
2015
2020
2025
Regulatory Information
FDA Decision
Evaluation Studies
Development / Pilot
Publication / Results Posted
Publication / Posting of Results

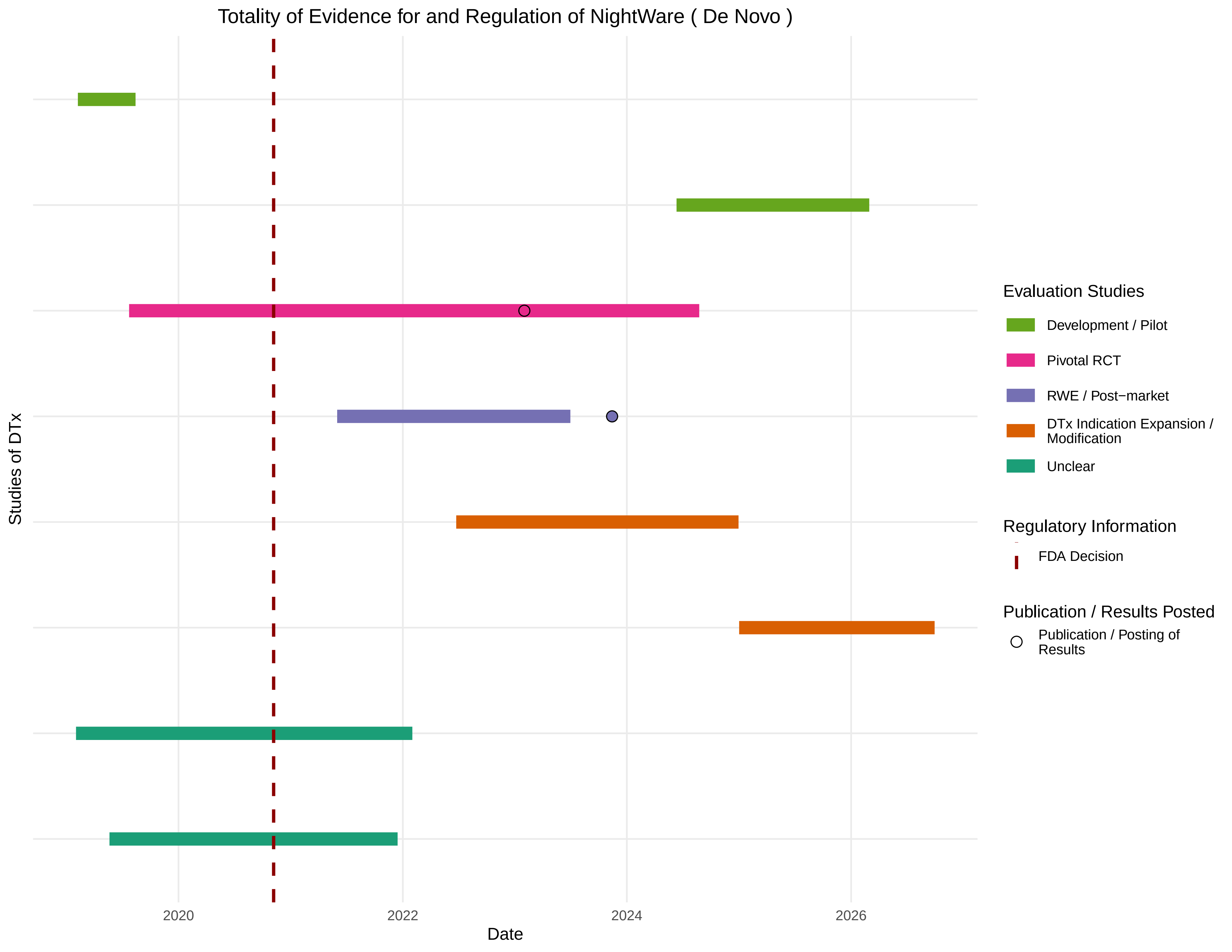

Totality of Evidence for and Regulation of NightWare ( De Novo )
Studies of DTx
2020
2022
2024
2026
Date
Evaluation Studies
Development / Pilot
Pivotal RCT
RWE / Post−market
DTx Indication Expansion / Modification
Unclear
Regulatory Information
FDA Decision
Publication / Results Posted
Publication / Posting of Results

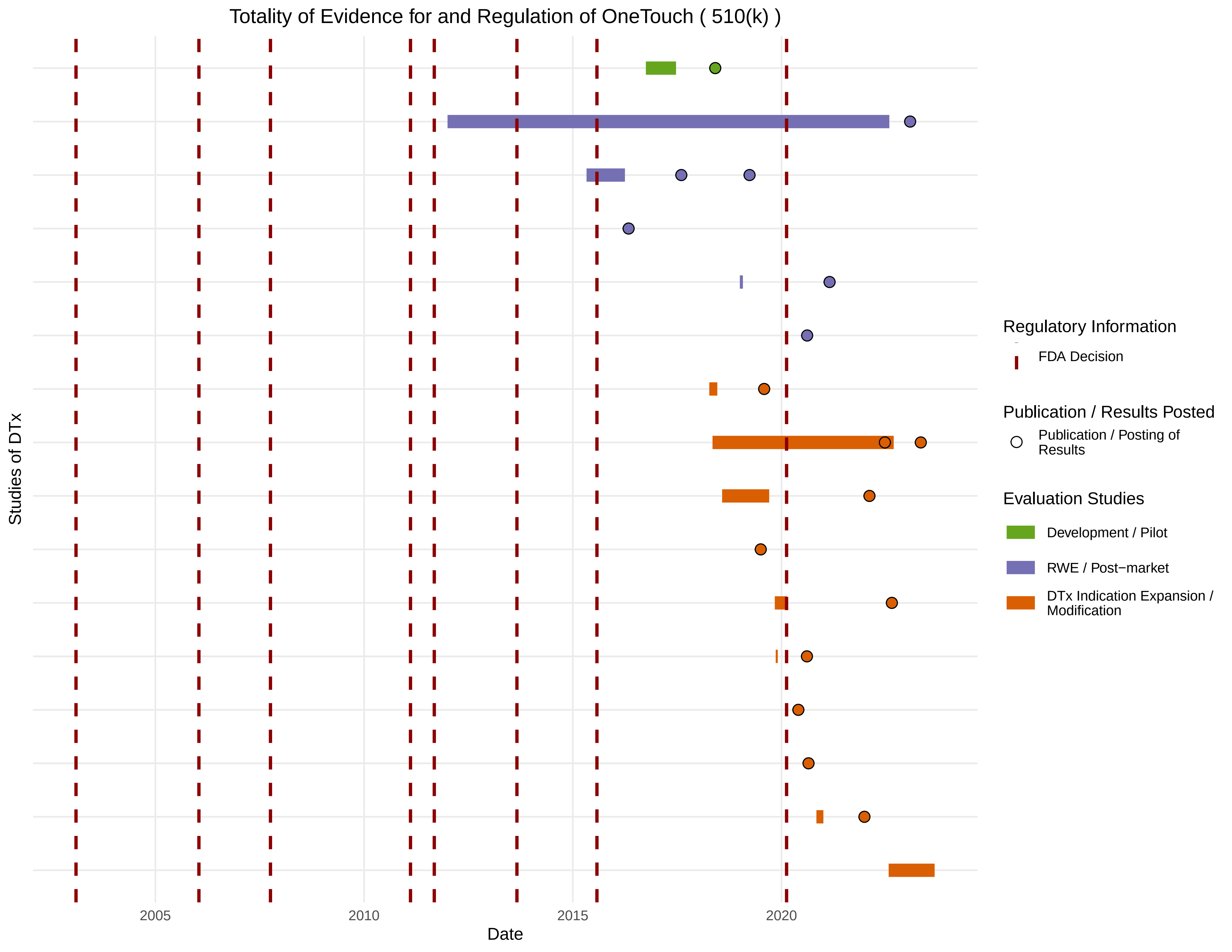
Totality of Evidence for and Regulation of OneTouch ( 510(k) )
Studies of DTx
Date
2005
2010
2015
2020
Regulatory Information
FDA Decision
Publication / Results Posted
Publication / Posting of Results
Evaluation Studies
Development / Pilot
RWE / Post−market
DTx Indication Expansion / Modification

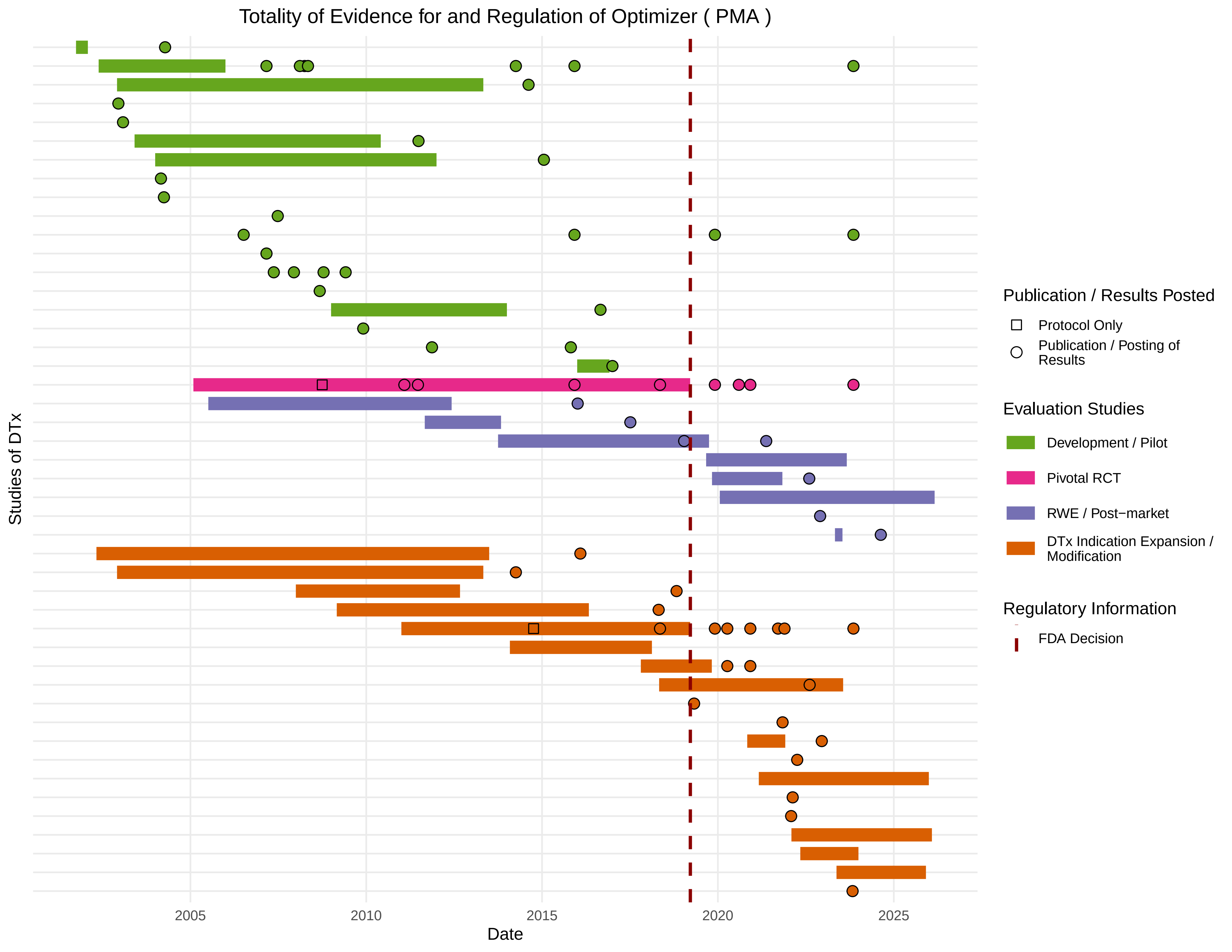

Totality of Evidence for and Regulation of Optimizer ( PMA )
Studies of DTx
Date
2005
2010
2015
2020
2025
Publication / Results Posted
Protocol Only
Publication / Posting of Results
Evaluation Studies
Development / Pilot
Pivotal RCT
RWE / Post−market
DTx Indication Expansion / Modification
Regulatory Information
FDA Decision

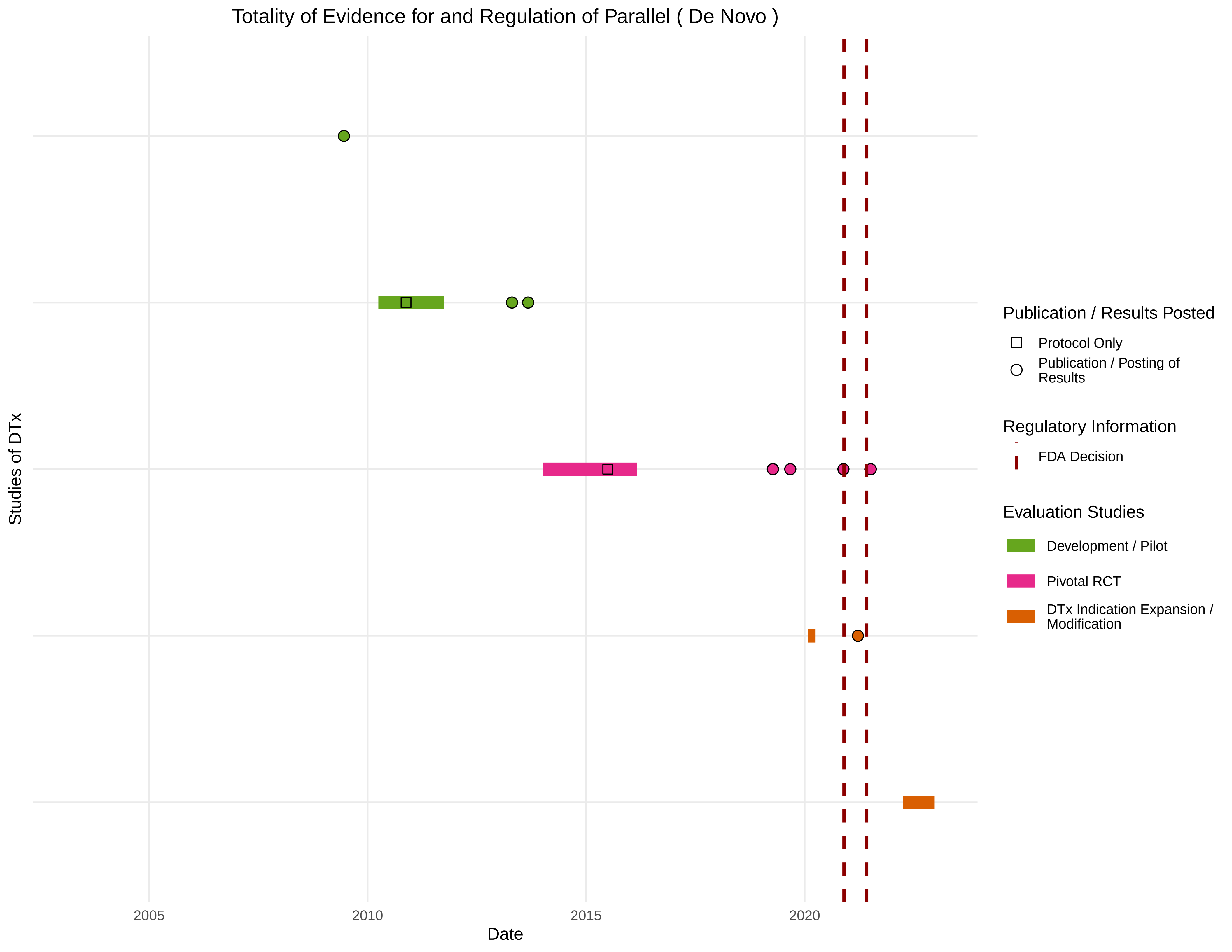

Totality of Evidence for and Regulation of Parallel ( De Novo )
Studies of DTx
2005
2010
2015
2020
Date
Publication / Results Posted
Protocol Only
Publication / Posting of Results
Regulatory Information
FDA Decision
Evaluation Studies
Development / Pilot
Pivotal RCT
DTx Indication Expansion / Modification

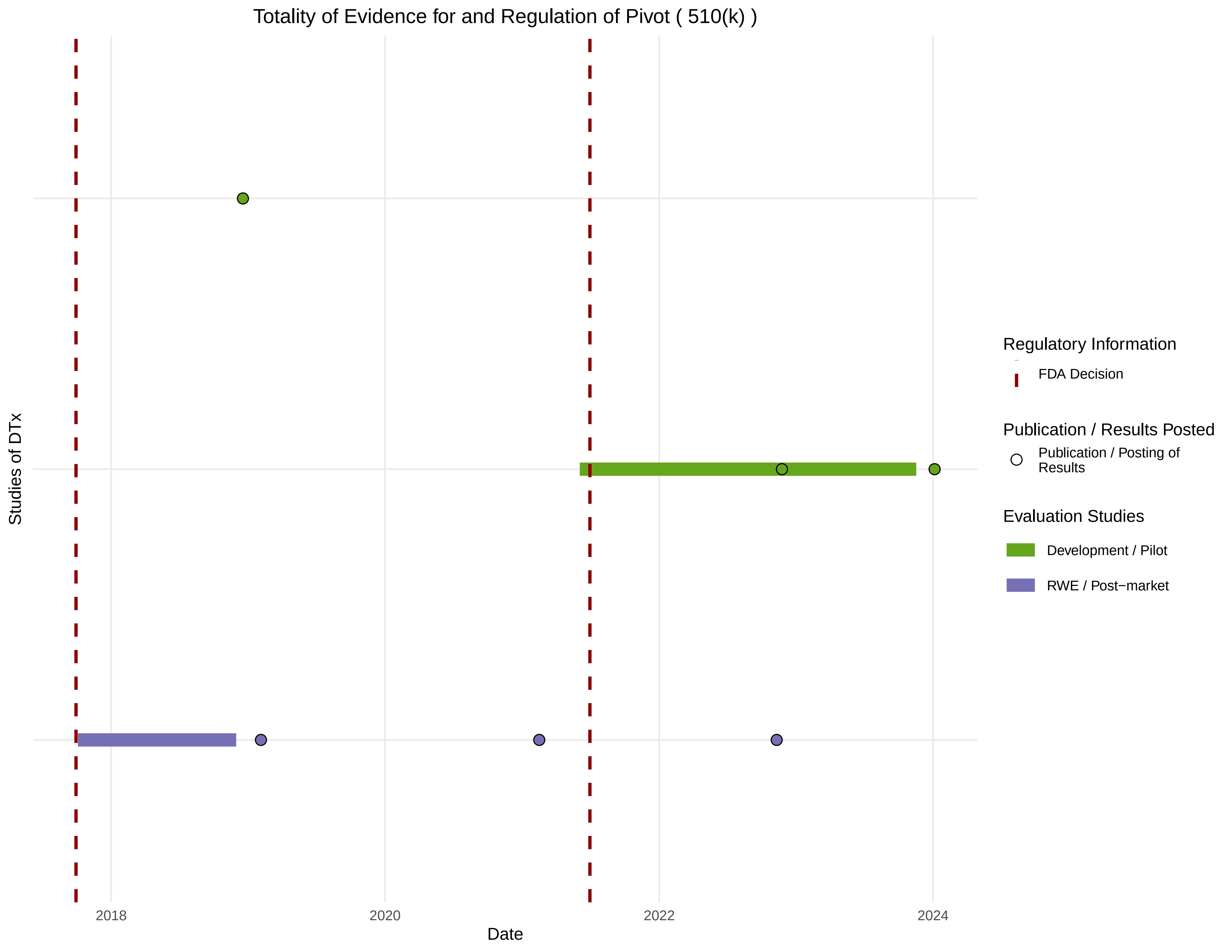
Totality of Evidence for and Regulation of Pivot ( 510(k) )
Studies of DTx
Date
2018
2020
2022
2024
Regulatory Information
FDA Decision
Publication / Results Posted
Publication / Posting of Results
Evaluation Studies
Development / Pilot
RWE / Post−market

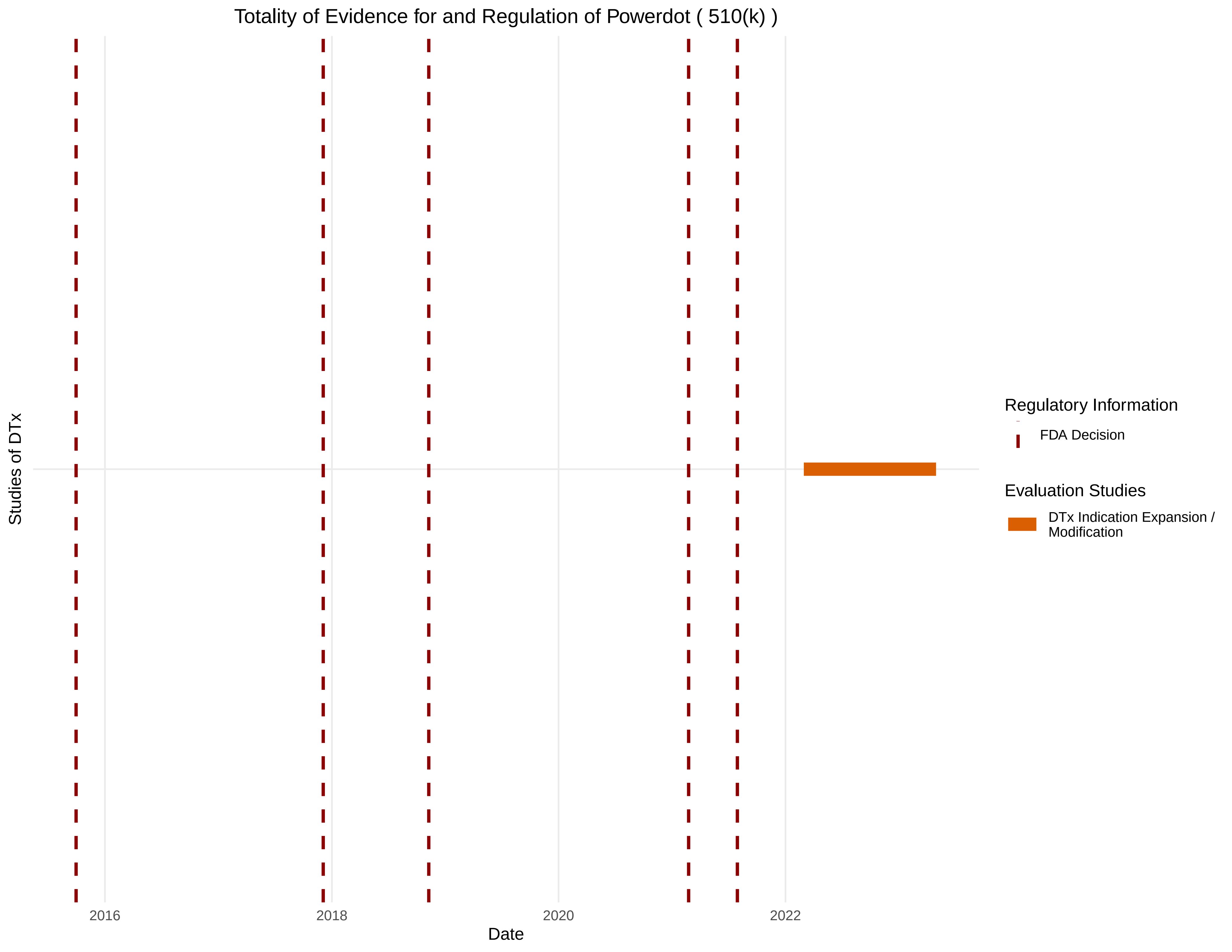
Totality of Evidence for and Regulation of Powerdot ( 510(k) )
Studies of DTx
2016
2018
2020
2022
Date
Regulatory Information
FDA Decision
Evaluation Studies
DTx Indication Expansion / Modification

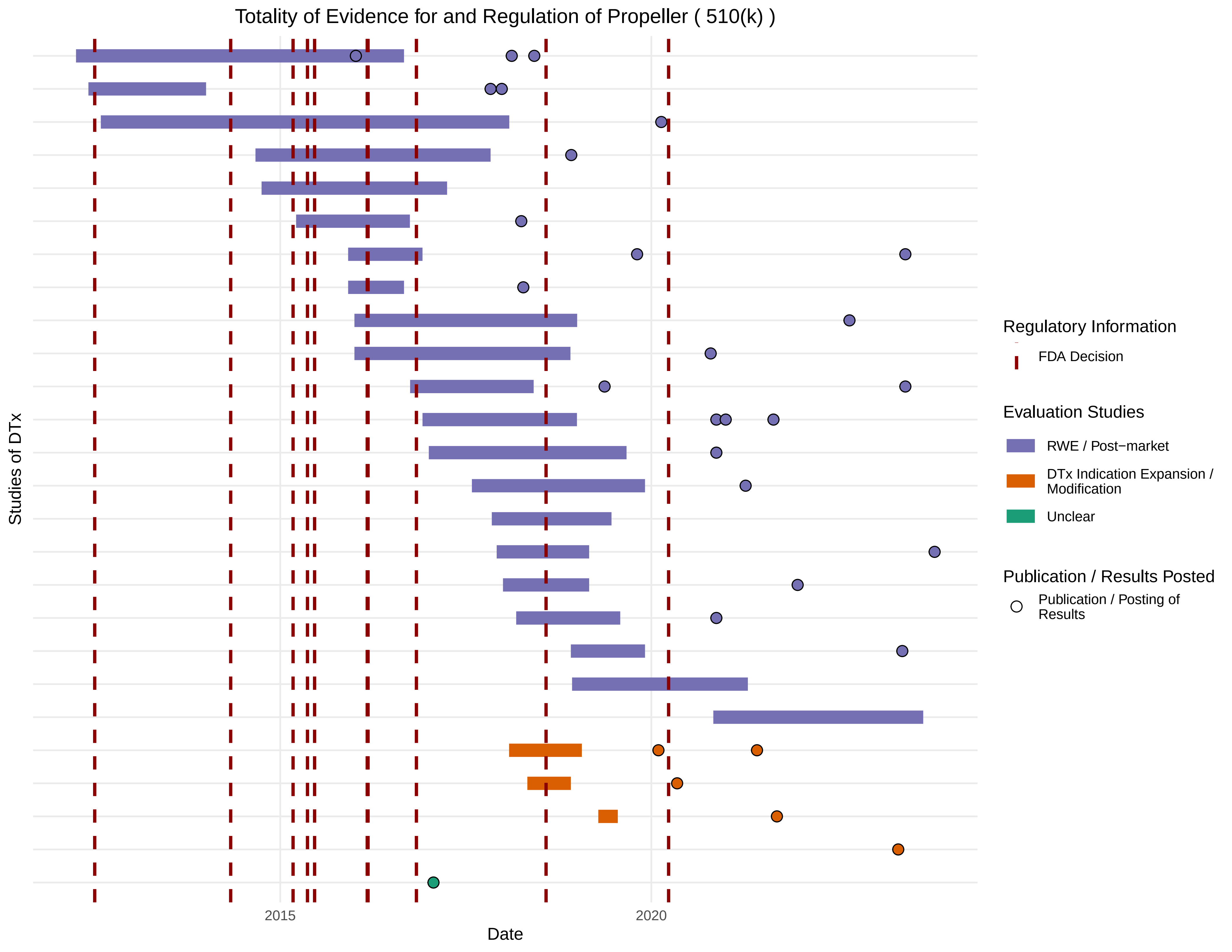

Totality of Evidence for and Regulation of Propeller ( 510(k) )
Studies of DTx
2015
2020
Date
Regulatory Information
FDA Decision
Evaluation Studies
RWE / Post−market
DTx Indication Expansion / Modification
Unclear
Publication / Results Posted
Publication / Posting of Results

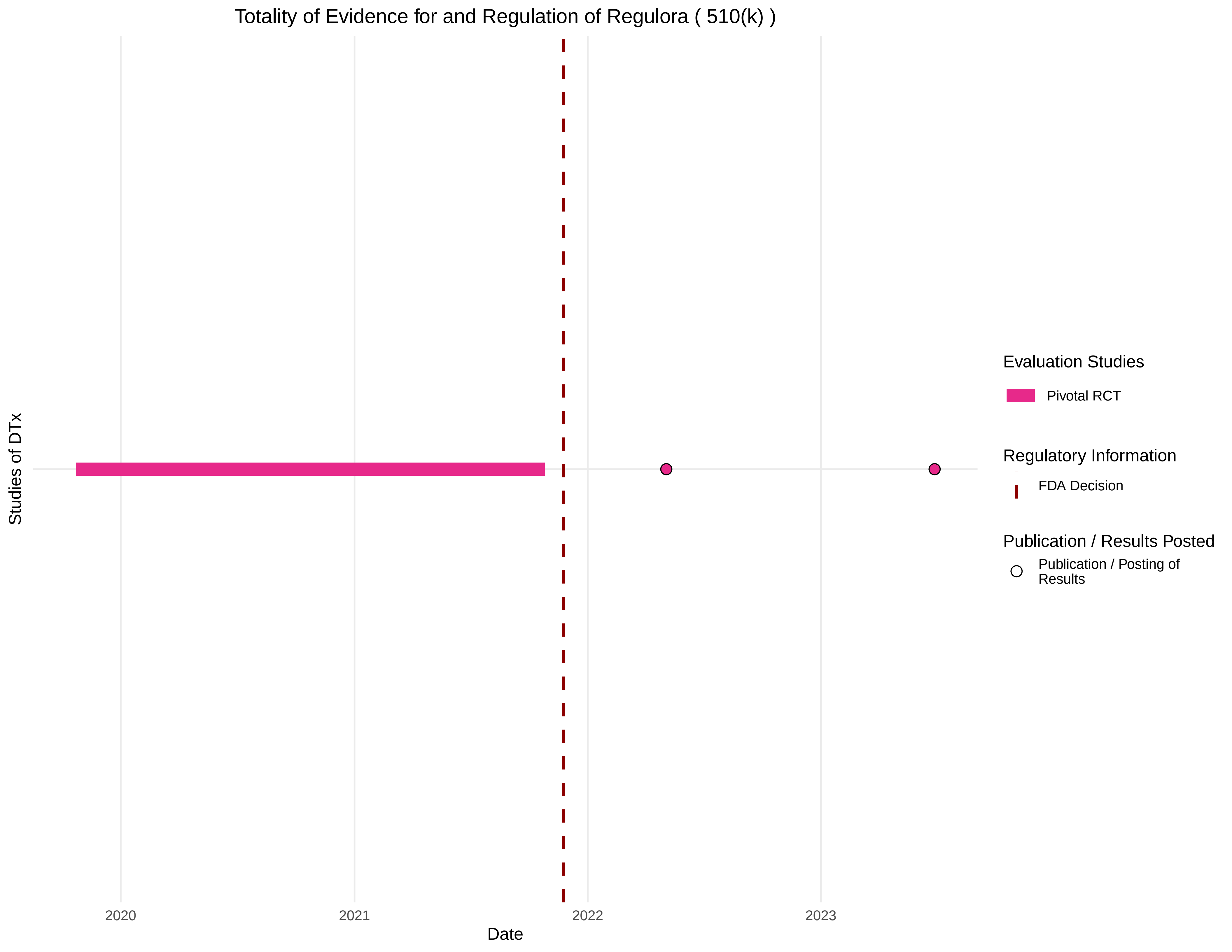
Totality of Evidence for and Regulation of Regulora ( 510(k) )
Studies of DTx
Date
2020
2021
2022
2023
Evaluation Studies
Pivotal RCT
Regulatory Information
FDA Decision
Publication / Results Posted
Publication / Posting of Results

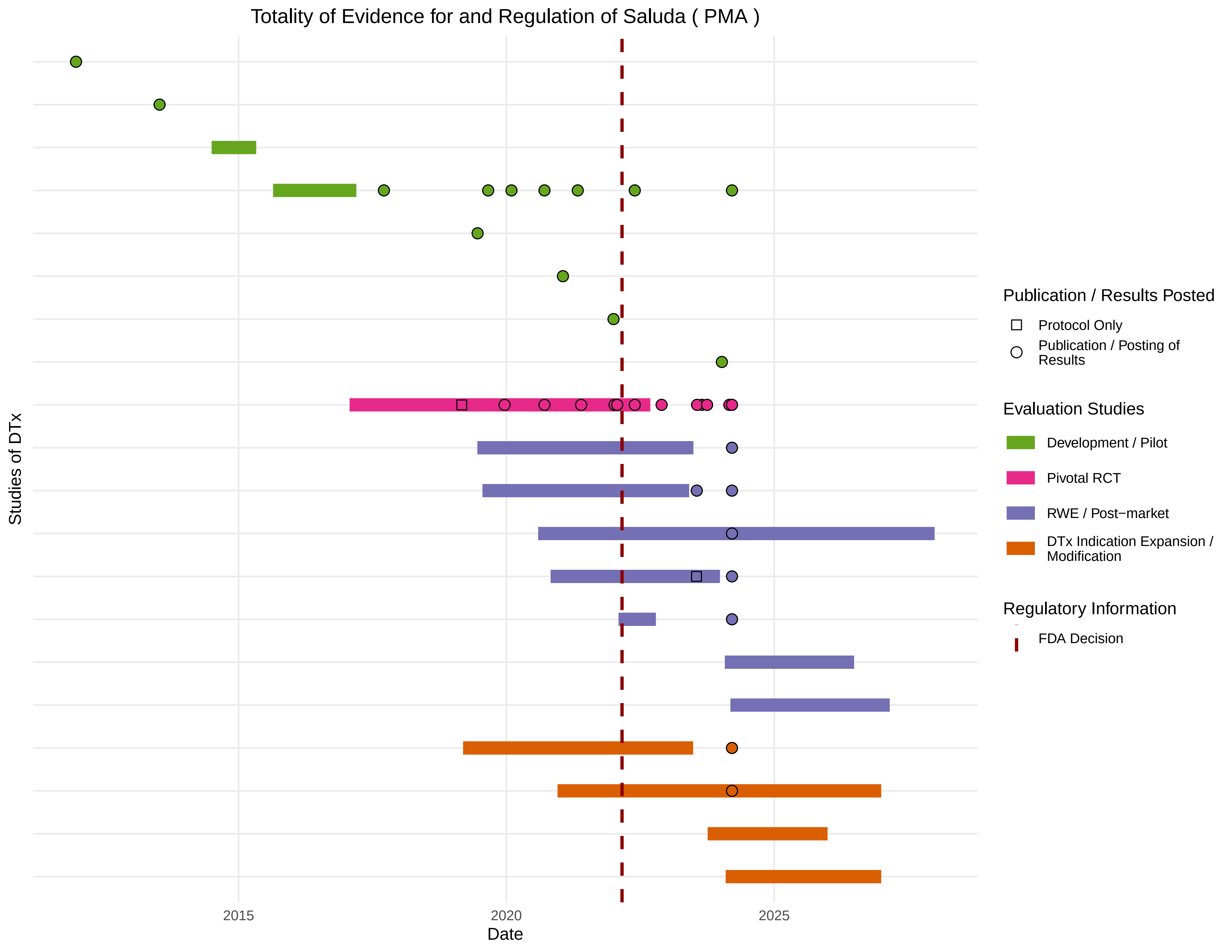

Totality of Evidence for and Regulation of Saluda ( PMA )
Studies of DTx
Date
2015
2020
2025
Publication / Results Posted
Protocol Only
Publication / Posting of Results
Evaluation Studies
Development / Pilot
Pivotal RCT
RWE / Post−market
DTx Indication Expansion / Modification
Regulatory Information
FDA Decision

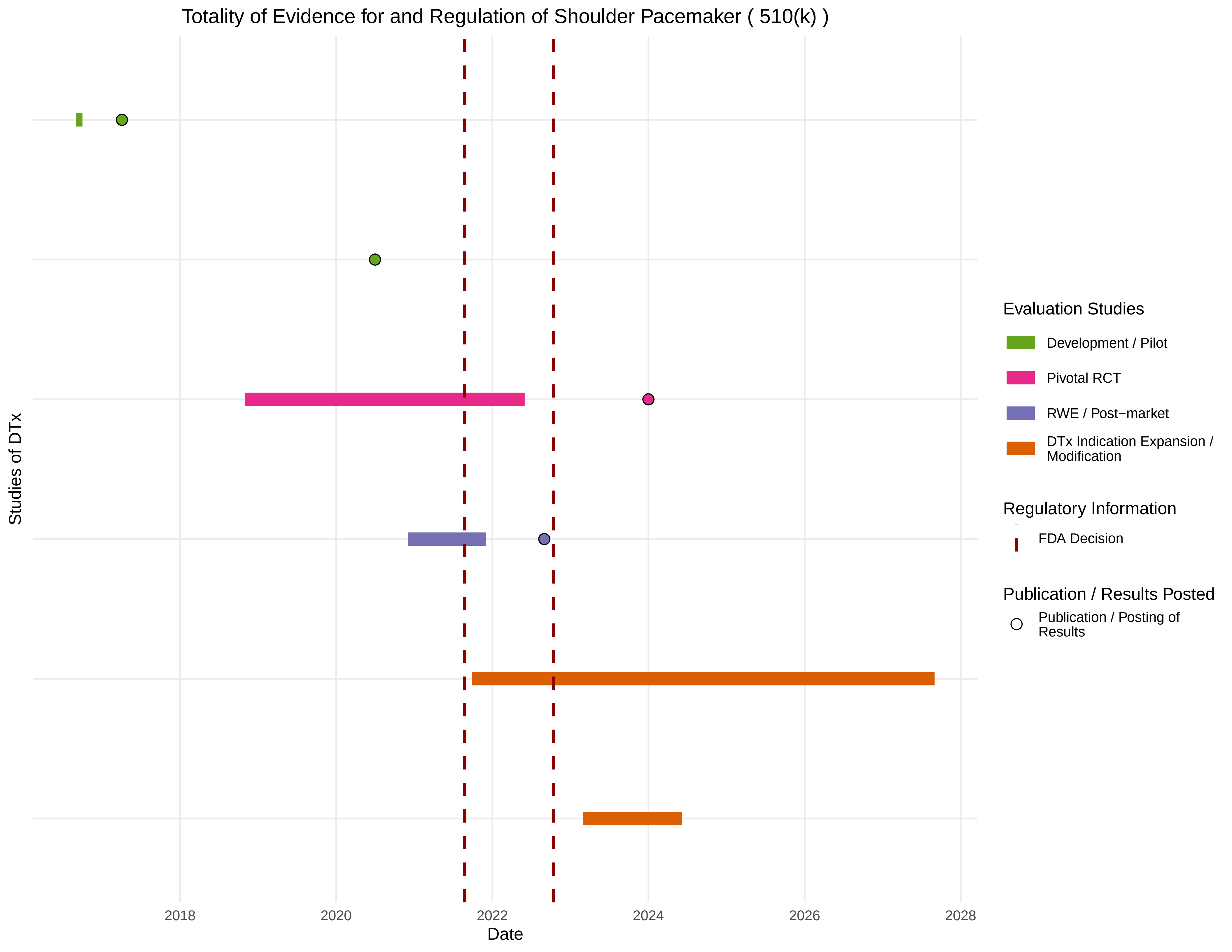
Totality of Evidence for and Regulation of Shoulder Pacemaker ( 510(k) )
Studies of DTx
Date
2018
2020
2022
2024
2026
2028
Evaluation Studies
Development / Pilot
Pivotal RCT
RWE / Post−market
DTx Indication Expansion / Modification
Regulatory Information
FDA Decision
Publication / Results Posted
Publication / Posting of Results

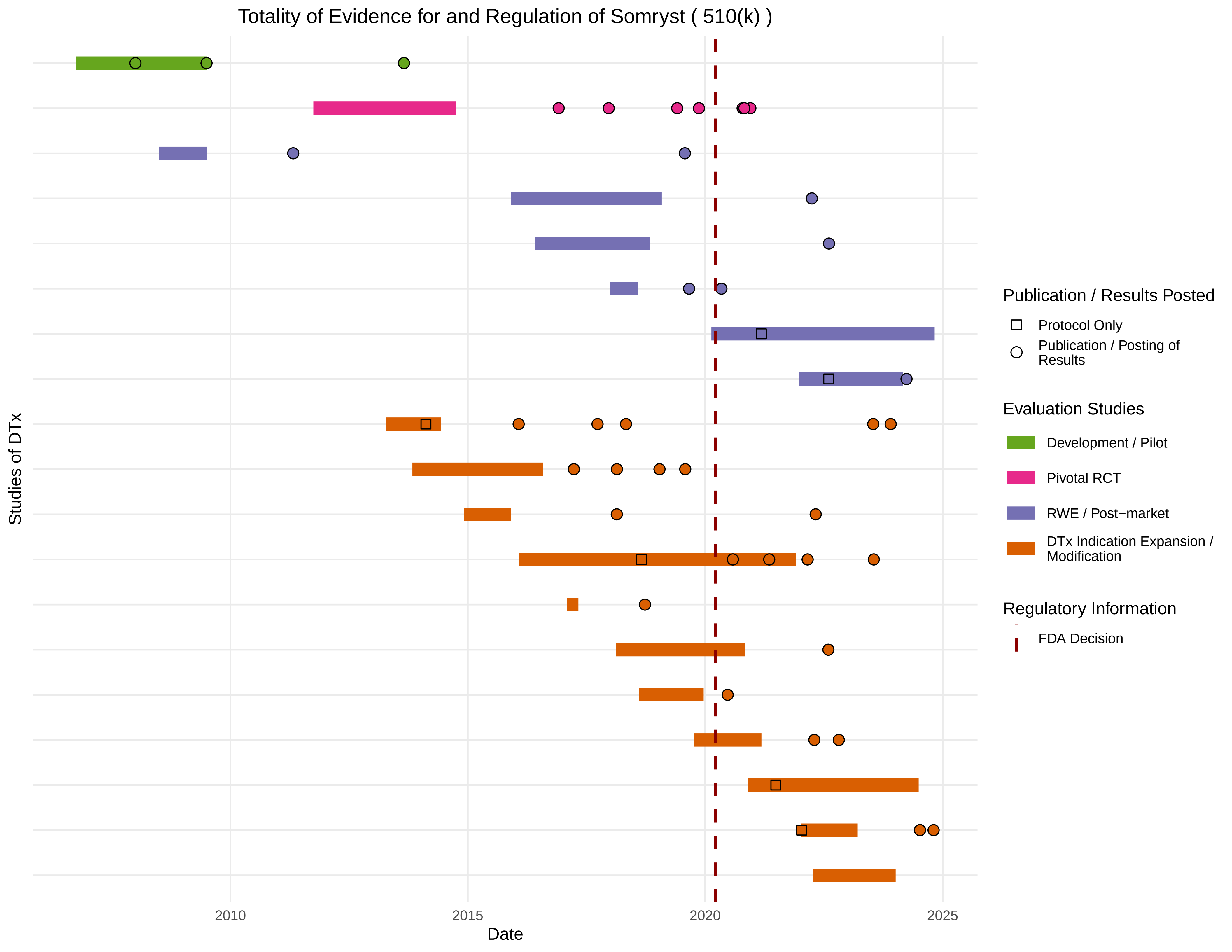

Totality of Evidence for and Regulation of Somryst ( 510(k) )
Studies of DTx
Date
2010
2015
2020
2025
Publication / Results Posted
Protocol Only
Publication / Posting of Results
Evaluation Studies
Development / Pilot
Pivotal RCT
RWE / Post−market
DTx Indication Expansion / Modification
Regulatory Information
FDA Decision

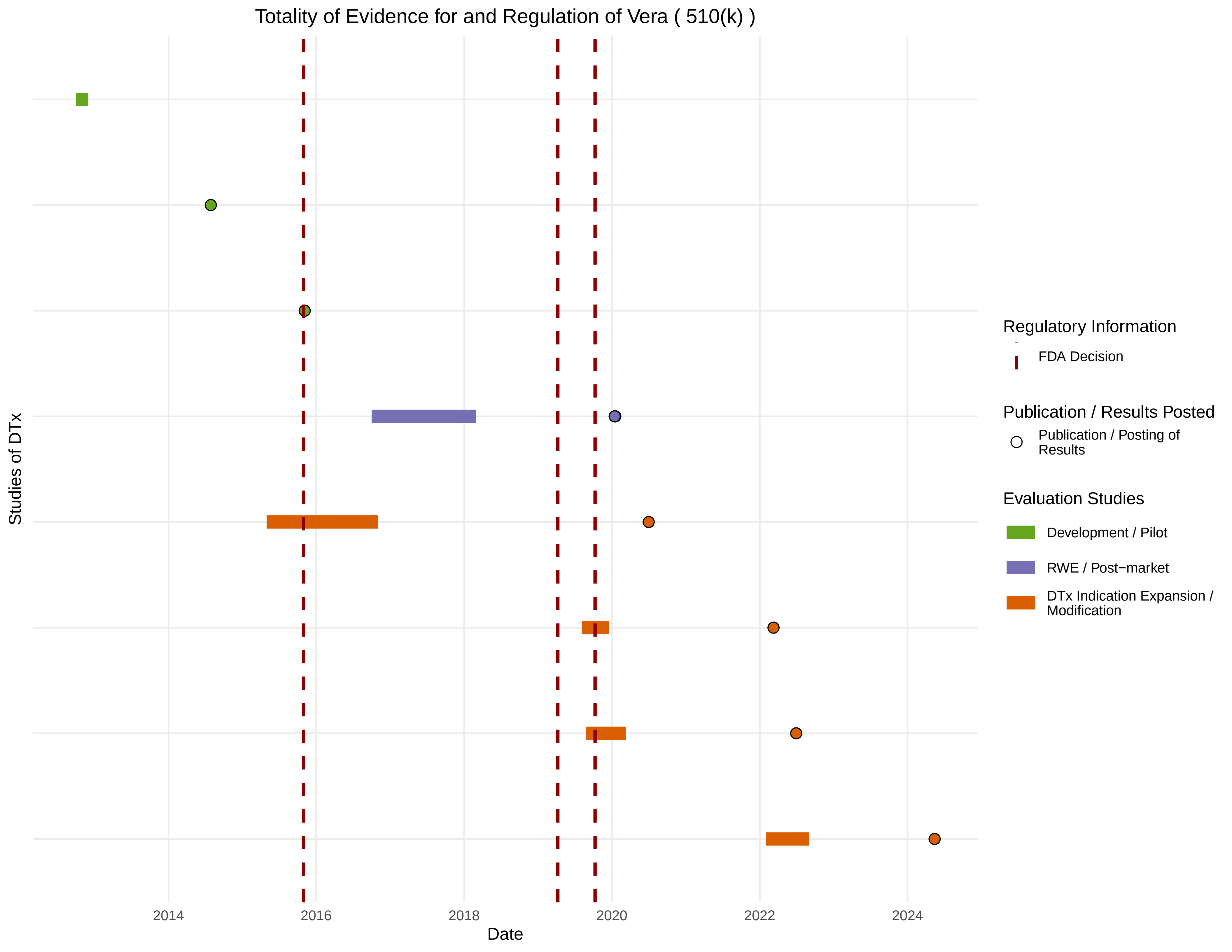
Totality of Evidence for and Regulation of Vera ( 510(k) )
Studies of DTx
Date
2014
2016
2018
2020
2022
2024
Regulatory Information
FDA Decision
Publication / Results Posted
Publication / Posting of Results
Evaluation Studies
Development / Pilot
RWE / Post−market
DTx Indication Expansion / Modification
